# A systematic review of evidence that environmental contaminant exposure impedes weight loss and glycemic control during calorie‐restricted diets in humans

**DOI:** 10.1111/obr.13886

**Published:** 2024-12-23

**Authors:** Kimberley Ann Bennett, Calum Sutherland, Anne Louise Savage

**Affiliations:** ^1^ Department of Built Environment and Life Sciences, Faculty of Social and Applied Science, Kydd Building Abertay University Dundee UK; ^2^ School of Medicine Dundee University UK

**Keywords:** diet‐induced weight‐loss, environmental chemical exposure, obesity management, type 2 diabetes remission

## Abstract

Calorie‐restricted diets cause weight loss and can drive type 2 diabetes remission. However, many patients struggle to achieve clinically relevant weight loss, and the reasons are not well understood. Chemical exposure is associated with obesity and type 2 diabetes development, and some evidence from preclinical experiments suggests it can limit the clinical benefits of calorie restriction. We systematically reviewed the evidence for the effects of environmental chemical exposure on mass loss and glycemic control during diet‐induced weight management in humans (PROSPERO: CRD42022339993). Of 222 unique citations, only six papers directly examined this question. Only one targeted people with type 2 diabetes. One linked phthalates and parabens, but not bisphenols, with slower fat loss. Two showed per‐ and polyfluoroalkyl substances were not associated with mass loss, but with faster subsequent mass regain. One linked impaired adiposity improvements with air pollutants. Two papers reported weight loss‐induced elevation in plasma organochlorines associated with altered glycemic control. The risk of bias largely arose from the potential for deviation from the intended diet, and statistics and reporting. The role of chemical exposure in impeding the effectiveness of weight management programs needs to be better understood to provide suitable support to people living with obesity and type 2 diabetes.

**Abbreviations**
AhR: aryl hydrocarbon receptor
BF%: percentage body fat
BMI: body mass index
BPA: bisphenol A
CI: 95% confidence interval
DDE: dichlorodiphenyldichloroethylene (a break down product of DDT)
DDT: dichlorodiphenyl trichloroethane
DINCH: 1,2‐cyclohexane dicarboxylic acid diisononyl ester
EDCs: endocrine‐disrupting chemicals
EFSA: European Food Safety Agency
GC: gas chromatograph
GC‐ECD: GC with electron capture detection
GC–MS: GC with mass spectrometer
GLP‐1: glucagon‐like peptide
GTTs: glucose tolerance tests
HbA1c: glycated hemoglobin A1c
HOMA‐IR: homeostatic model of assessment for insulin resistance
HOMA‐β: HOMA for β cell function
LC–MS/MS: liquid chromatograph coupled to a tandem mass spectrometer
NHANES: National Health and Nutrition Examination Survey
NOAEL: no observed adverse effect level
OCP: organochlorine pesticide
PCBs: polychlorinated biphenyls
PECO: population, exposure, context, outcome
PFAS: per‐ and polyfluoroalkyl substances
PFDA: perfluorodecanoic acid
PFHxS: perfluorohexanesulfonic acid
PFNA: perfluorononanoic acid
PFOA: perfluorooctanoic acid
PFOS: perfluorooctanesulfonic acid
PM_10_
: particulate matter with diameter of 10 μm
PM_2.5_
: particulate matter with diameter of 2.5 μm
POPs: persistent organic pollutants
PRISMA: preferred reporting items for systematic reviews and meta‐analysis
RMR: resting metabolic rate
RoB: risk of bias
T2D: type 2 diabetes
TASH: toxin‐associated steatohepatitis
TWI: tolerable weekly intake
WC: waist circumference

## 1 INTRODUCTION

The most effective way to reduce and reverse type 2 diabetes (T2D) without surgery is through substantial, intentional weight loss, and to maintain that lower weight.^1^, ^2^, ^3^ In the first 12 months of the DiRECT study, an intensive program for weight loss and maintenance, 46% of participants achieved T2D remission compared to 4% receiving usual care. Remission was maintained in a third of patients over 2 years and was > 85% in those who achieved 10 kg weight loss.^2^ Non‐obese people with T2D also benefit from weight loss, likely because it reduces ectopic lipid storage.^4^, ^5^ Weight loss lowers the risk of developing T2D in overweight individuals without T2D more effectively than T2D medications.^6^ One kg of weight loss can reduce T2D risk by 20%.^7^

However, even within successful programs many participants fail to lose weight or achieve glycemic control once weight loss has been accomplished.^3^ Sex, initial glycated hemoglobin (HbA1c; a marker of glycemic control), initial body mass index (BMI), ethnic background, use of insulin, hypertension, and pre‐existing cardiovascular disease influence the trajectory of weight loss during calorie restriction.^8^, ^9^, ^10^, ^11^, ^12^ Nevertheless, these parameters cannot predict response to diet.^9^, ^13^ Multi‐omic blood or adipose biomarkers can help to differentiate weight and glycemic responders from non‐responders but are inconsistent between studies,^14^ improving prediction accuracy to only 75% [67–83% CI].^9^, ^13^ Gut microbiome gene signatures^15^ and gene variants associated with efficacy of weight loss^16^ may modify responses to dietary interventions. Genotype‐diet interactions do not fully explain poor weight loss in calorie‐restricted diets.^17^ Understanding and accounting for the factors that modify the response to calorie restriction remains an ongoing challenge.

Increasingly, a wide range of chemicals that have previously been identified as carcinogenic or impairing fertility, through endocrine and metabolic disrupting effects, are implicated in T2D and obesity epidemics^18^, ^19^ and may exacerbate obesogenic and diabetogenic environments, especially in those with underlying genetic susceptibility.^20^ Humans are exposed to a vast array of anthropogenic environmental contaminants in air, water, food, and through contact with everyday items.^21^ These include legacy contaminants, such as heavy metals, organophosphate pesticides, and persistent organic pollutants (POPs) that are now banned or restricted in many countries but remain in the environment because they are stable, often lipophilic, and bioaccumulative. Emerging pollutants that have been more recently identified as health risks are also present in everyday items. They include phthalates and bisphenols, such as BPA, that are used as plasticizers; the next‐generation persistent compounds, perfluoroalkyl and polyfluoroalkyl substances (PFAS), termed ‘forever chemicals’; and more recently developed pesticides, such as neonicotinoids. Systematic reviews of the epidemiological literature indicate that many chemical classes can impact the development of T2D or obesity (Table S1), although such effects are often small. Most literature in this field typically focuses on a limited number of chemical classes, making it challenging to know which should be the highest priority for research, regulation, and screening.

Animal and in vitro studies support the human study findings and provide mechanistic insight.^22^, ^23^, ^24^, ^25^ Mechanisms of action are often shared between chemical classes. Adipocyte gene regulation by several chemical classes affects cultured adipocyte cell fate and function, while in vivo studies show alterations to adipose development, fat accumulation, and impaired adipocyte function. Chemical impairment of mitochondrial function lowers metabolic rate and alters fuel preference, processes linked to dysfunction of pancreatic beta cells, leading to impaired insulin responses. Endocrine‐disrupting chemicals (EDCs) have well‐established effects on thyroid hormone and estrogen production, transport, action, and elimination that can alter energy balance, promoting mass gain. Impacts on insulin and glucagon‐like peptide (GLP‐1) signaling have also been identified. Parabens have been linked to altered appetite‐regulating neurotransmitters in the hypothalamus^26^ in in vivo and human studies. Initiating gut dysbiosis through alterations in microbiome composition towards species that enhance absorption and/or gastrointestinal inflammation is a further, indirect route through which chemical exposure may influence energy balance. During weight loss, lipophilic contaminants sequestered in adipose tissue are mobilized leading to a spike in circulating levels that may dampen the health benefits of weight loss.^27^, ^28^ Chemicals could thus impact weight and glycemic control in humans through multiple avenues, including 1) disconnecting weight loss and subsequent improvement in glucose homeostasis; 2) direct actions on glucose homeostasis; 3) altering adipose biology to influence lipid storage and adipokine release with subsequent impact on appetite/satiety as well as lipid homeostasis; and 4) endocrine disruption of metabolic hormones. The range of mechanisms and chemical classes implicated in metabolic disruption (reviewed in^19^ are diverse and all deserve attention.

Our recent review highlighted the paucity of preclinical research on the role of chemical exposure in impeding weight loss and glycemic control during calorie restriction.^29^ In mice, polychlorinated biphenyls (PCBs) do not impact mass loss but are consistently associated with worse glycemic control after a calorie‐restricted diet, while the impacts may be sex‐specific and dependent on intact aryl hydrocarbon receptor (AhR).^22^, ^23^, ^25^ However, these data come from only one research group working on mice of only one genetic background and have not yet been replicated in other species. Similarly, a single study shows that PFAS can impair the beneficial effects of weight loss in mice.^24^ In rats, the organochlorine pesticide (OCP) dichloro‐diphenyl‐trichloroethane (DDT) increases weight loss.^30^ The impacts of other chemical classes have not been reported.^29^ Such limited information from animal studies makes it hard to identify which chemical groups are likely to be most problematic in humans. Indeed, rodent studies should probably focus on chemical classes with the largest impact on metabolism in humans.

To investigate whether that information can be uncovered from current literature we systematically examined studies investigating the effect of a wide range of environmental pollutant exposure on intentional, diet‐induced weight loss in humans to meet the following objectives: 1. map out the scope and nature of evidence that environmental pollutants impede weight management and subsequent glycemic control; 2. determine which chemicals are most commonly researched in a weight loss context, 3. determine the size of the effect of those chemicals on weight loss trajectory or resolution of glycemic control to establish which are most strongly associated with poor response to diet‐induced weight loss interventions; 4. appraise the available evidence; and 5. identify knowledge gaps that are important to address in future human intervention studies. This information will help determine whether chemical exposure history should be considered when delivering care for obesity and T2D.

## 2 METHODS

Study selection, screening, and data extraction followed preferred reporting items for systematic reviews and meta‐analysis (PRISMA) guidelines.^31^ Minor modifications to the registered protocol (PROSPERO CRD42022339993) are detailed below.

### 2.1 Search

We searched PubMed, Web of Knowledge, and Scopus for keywords in the title and/or abstract (Table S2). We used the population, exposure, context, and outcome (PECO) framework.^32^ The population included all human participants undergoing intentional, diet‐induced weight loss, comprising healthy individuals and those for whom intentional weight loss was used to manage obesity and/or T2D. We excluded papers in which weight loss occurred due to bariatric surgery, or unintentionally, and those that involved diets that were not weight loss interventions, such as replacement of calories with other macronutrients. For example, one study used either nuts or fatty fish versus a normal diet with these components depleted to establish whether fatty fish consumption increases POP exposure.^33^

We considered all studies that included any exposure (background, occupational or intentional) to a chemical of environmental concern that has been identified in at least one epidemiological study as a potential risk factor in the development of T2D or obesity, metabolic syndrome, insulin insensitivity, hepatic steatosis, or toxin‐associated steatohepatitis (TASH).^34^ We amended the registered protocol to include air pollutants (such as particulate matter, ozone, nitrogen oxide, and dioxide). We excluded pharmaceuticals to which people may be exposed in food and water but are normally taken as medication. There were no limits on study context or on diet duration or the nature of the intervention, so long as it was intended to cause weight loss.

The main outcome was the rate or magnitude of weight loss or fat reduction, including changes in body mass, percentage fat (BF%), BMI, waist circumference (WC), and other metrics associated with reduced adipose reserves. Secondary outcomes included measures of glycemic control (fasting glucose, fasting insulin, HbA1c, homeostatic model of assessment (HOMA) for insulin resistance (HOMA ‐IR), HOMA for β cell function (HOMA‐β), glucose tolerance tests (GTTs)) and liver fat content.

No language or date limits were applied. An initial, limited search to refine the terms was performed. New terms were only included if they expanded the number of relevant papers. The full search was performed on 4.4.2022, updated on 17.4.2023, 11.5.2023, 28.06.2023, 20.10.2023 and the most recent search was performed on 28.10.2024. Search results were imported into Excel and Nested Knowledge and duplicates were removed. The ‘nest’ synthesis is available at: https://nested-knowledge.com/nest/5901?key=f37c3d5aadfc4808319250b456f3911d

### 2.2 Screening

Two evaluators independently screened titles and abstracts. Primary research was included if it 1. explored the effect of a known environmental contaminant, listed in the search terms (Table S2); 2. if it examined intentional and diet‐induced weight loss; 3. if it reported weight loss as an outcome. Evaluators were unable to see each other's decisions until adjudication. Cohen's kappa was calculated for initial screening. When the evaluators disagreed on inclusion or exclusion, the disagreement was resolved by discussion.

References and citing publications of included papers were searched. Connected Papers (https://www.connectedpapers.com) was used to identify other eligible papers from each included paper. The registration reference of clinical trials from included papers was used to search for additional relevant publications. We subsequently excluded papers that examined contaminant redistribution or elimination during weight loss or assessed other toxicological markers since they did not set out to determine whether contaminants impacted weight loss rate or glycemic control

#### 2.2.1 Data synthesis

Study characteristics were extracted in Nested Knowledge and included: bibliographic information; study design, country, chemical(s) examined; matrix used to assess exposure and exposure assessment method; sex, age, outcomes measured, and sample size. Patient or volunteer population characteristics, dietary intervention type, duration, follow‐up number, interval, duration, and outcomes measured at follow‐up were extracted. We also recorded whether studies reported other factors that can modify mass loss and/or contaminant exposure, such as sex, age, ethnicity, and duration of T2D, and whether these factors were also included in the analysis. We attempted to extract the absolute or relative reduction in mass (kg), BMI, WC (cm), % BF, change in glycemic control, or liver steatosis to compare these between the highest and lowest exposure groups or to extract the slope of the line where regression was performed exploring the relationship between these changes and initial exposure.

#### 2.2.2 Risk of bias

We used the modified ROBINS‐I tool,^35^ designed for assessing risk of bias (RoB) in non‐randomized studies on the effect of unintentional exposure. We developed bespoke signal questions to ensure clarity and consistency between evaluators since there was both a deliberate intervention (the diet) and an unintentional exposure (chemical levels). Questions were cross‐checked by all three authors before use. We classified the risk of bias as low moderate or high^36^ (Table S3). The overall study RoB was allocated based on the highest rating across domains. Independent responses from two evaluators were collected in Office 365 forms. Disagreements between evaluators were resolved by discussion. RoB was summarized using the RoBvis tool (https://www.riskofbias.info/welcome/robvis-visualization-tool).^37^

#### 2.2.3 Evidence synthesis

We summarized the study design, sample size, location, chemical, and outcomes measured and tabulated study characteristics.

## 3 RESULTS

### 3.1 PRISMA

We retrieved 188 unique records after deduplication from databases, and identified a further 34 from searches of citing/cited papers and Connected papers (Figure S4; S5). Full texts were not available for four older papers. Title and abstract screening excluded 167 papers. Full text screening of remaining papers excluded case studies, animal studies, papers that involved bariatric surgery, papers in which the diet was not intended to cause weight loss (e.g.^33^), papers that assessed redistribution or elimination of chemicals during weight loss but not their effect on outcomes of interest. Agreement between reviewers after initial independent full text screening was 86.7%. Cohen's kappa was 0.66 [0.54,0.77 95% CI].

We were left with six eligible papers (Table 1). Screening/searching details are available (Open Science Framework: https://osf.io/ckjpt/?view_only=af2a04807edb4cfdaba759a2e135e584). Study characteristics are provided in Table S6.

**TABLE 1:** Summary characteristics of papers included in a systematic review of evidence that contaminant exposure impairs weight loss and impedes resolution of glycemic control during diet‐induced weight loss in humans. # indicates where one paper investigated several chemical groups. $ indicates where one study covers several continents. Shading represents number of studies in that category from 0 = lightest to 6 = darkest.

		Study design					Initial total sample size					Location				Chemicals of interest								Outcomes			
		Post hoc analysis of RCT	RCT	Case control	Retrospective cohort	Prospective cohort	15–30	30–50	50–100	100–200	200+	Europe	North America	Africa	Asia	Chlorinated POPs	Brominated POPs	PFAS	Plasticisers	Parabens	Heavy/toxic metals	Air pollutants (PM_2.5_;PM _10_)	Organophosphate	Mass	BMI	Other adiposity metrics	Glycaemia control metrics
*N* (papers)	6	4	0	0	1	1	0	1	1	0	4	4 ^$^	3^$^	1^$^	1^$^	2	0	2	1^#^	1^#^	0	1	0	4	5	4	3

### 3.2 Overview

POPs were most frequently examined. Two papers investigated chlorinated compounds^38^, ^39^ and two investigated fluorinated compounds.^40^, ^41^ Plasticizers, including phthalates and BPA, and personal care products, including parabens^42^ were examined in one paper. Retrospective analysis of air pollutants from five years previously was reported by Ustulin et al.^43^ Other chemical groups that have been linked to obesity or T2D development, such as heavy metals and organophosphate pesticides, brominated POPs, phthalate and BPA replacements, have not yet been investigated in this context. Recruitment of participants spanned from 1991 to 2014 (Figure S7). Five of the six papers used retrospective analysis of samples from older trials.

All papers examined background exposure rather than populations that have experienced accidental or occupational exposure to high chemical levels, or those at higher risk for other reasons, such as residing close to chemical plants or from their normal diet. Only three papers^40^, ^41^, ^42^ provided information on how chemical levels at the start of the study affected the outcomes of interest in this review. Figure S8 summarizes information flow in the current evidence base.

### 3.3 Chlorinated POPs

Both papers investigating chlorinated POPs (OCPs and PCBs) explored the impact of exposure on glycemic control.^38^, ^39^ Kahleova, et al^39^ reported post hoc analysis from a European clinical trial (*n* = 74) in which participants were pre‐selected by their endocrinologists,^44^ whereas Imbeault, et al^38^ was a small North American prospective cohort study (*n* = 37) in which participants were recruited through general adverts.

Imbeault, et al^38^ reported that the greater the increase in plasma OCPs during the diet intervention, the greater the reduction in fasting plasma insulin (Table S9). This is suggestive of improved diabetes risk. The association occurred in men but not women and was significant for hexachorbenzene and Aroclor 1260 but not for *p–p’‐* dichlorodiphenyldichloroethylene (DDE), lindane, or CB153 when adjusted for body fat loss. Men experienced an overall greater reduction in fasting insulin than women and increased levels of several OCs during the diet whereas women did not. In contrast, fasting glucose or glucose response during GTT did not change during weight loss and was not associated with a change in levels of the OCs measured, but these data and details of the analyses are not reported (Table S9).

Kahleova, et al^39^ did not report fasting insulin levels, preventing comparison with Imbeault, et al.^38^ They found that the greater the increase in POPs during the weight loss phase, the greater the drop in both fasting glucose and HbA1c, but the more muted the increase in β‐cell function (Table S9). No sex differences were investigated. The POPs with predictive power in the analysis included a range of PCBs, but not *p–p’‐*DDE or CB153. Absolute values for metabolic parameters and their changes were not reported making it difficult to determine the effect size. A lack of association between POP release during weight loss and whole‐body insulin sensitivity was reported. No details were given about POP associations with mass, BMI, and visceral fat volume despite the availability of those measurements (Table S10).

There were key technical differences and very different chemical profiling between these studies preventing their comparison, including the matrix, chemicals examined (Table S11), clean‐up and defatting processes, detection method (gas chromatography (GC) followed by either electron capture (GC‐ECD) or mass spectrometry (GC–MS)) that provide differences in resolution of the equipment, and correction for lipid weight (Table S12; S13). It is not always clear what internal standards were used or whether procedural blanks were used. In addition, the change in POPs during the intervention is reported and analyzed, rather than the baseline values in both papers.^38^, ^39^

The diets lasted 12–15 weeks and consisted of a 500‐kcal^39^ or a 700‐kcal deficit.^38^ Kahleova, et al^39^ reported HbA1c and fasting plasma glucose and undertook isoglycemic hyperinsulinaemic clamps to measure insulin sensitivity. Insulin levels and response to GTT were also reported in Imbeault, et al.^38^

### 3.4 PFAS

As post‐hoc analyses of large clinical trials in the northern hemisphere both papers that examined PFAS explored the impact on weight loss trajectory^40^, ^41^ and Liu et al^40^ additionally explored the impact on glycemic control. Liu, et al^40^ reported on indices of adiposity (mass, BMI, BF%, and waist circumference), and glycemic control (Hb1Ac, fasting glucose, insulin, and HOMA ‐IR and HOMA‐β). Glycaemia control and adiposity metrics were also measured as part of the DioGenes study,^45^ but were not analyzed.^41^ PFASs were not associated with weight loss but were associated with faster subsequent weight regain,^40^, ^41^ and a greater reduction in metabolic rate during weight loss^40^ (Table S9). Grandjean, et al^41^ does not report the effect size of PFAS on weight loss because the paper focuses on the weight maintenance period after the low‐calorie diet phase. They report a weak association between PFAS and relative weight loss during the low‐calorie phase but provide no details of these analyses. Initial median plasma levels of 5 PFAS are reported in three groups of low (5.6–9.2 kg), medium (9.2–11.8 kg), and high (11.8–28,3) weight loss categories. The *p*‐values presented from ANOVA suggest that the group exhibiting the greatest weight loss had higher initial levels of perfluorohexanesulfonic acid (PFHxS) and perfluorooctanoic acid (PFOA) than the group that experienced the lowest weight loss, but these data are heavily confounded by initial body mass and sex. A doubling of PFOA resulted in a 1.5 kg greater weight regain during the 26‐week weight maintenance phase after low‐calorie intervention and 1 kg greater weight gain for a doubling of each of the remaining four PFAS^41^ (Table S9). G‐computation, which can account for non‐linear relationships in multivariate statistics,^46^ suggested that a doubling of all PFAS resulted in 1.66–1.71 kg greater weight gain across 26 weeks and of these PFHxS, PFOA and perfluorodecanoic acid (PFDA) were responsible for the overall positive effect, while perfluorooctanesulfonic acid (PFOS) and perfluorononanoic acid (PFNA) had weaker negative effects. A more than 2 kg difference was thus predicted between those in the lowest and highest PFAS exposure tertiles at 26 weeks post‐diet.

Liu, et al^40^ reported a 2.5‐to‐4‐fold decrease in resting metabolic rate (RMR) from lowest to highest PFDA tertile (Table S9), after adjusting for confounds (Table S10). Similarly, baseline PFOS and PFNA were associated with 8–9‐fold and 8–16‐fold reductions in RMR, respectively, across tertiles of each chemical during the weight loss diet. Several of the PFAS measured were associated with greater mass regain over 6–24 months post diet after adjustment for confounds (PFOS = 1.7–1.8 kg; PFNA = 1.4–1.7 kg; and PFDA = 1.2 kg difference in mass regain between lowest and highest tertiles), consistent with Grandjean, et al.^41^ The highest tertile baseline PFAS was associated with persistently lower RMR compared to the lowest at the 24‐month post diet follow up. Liu, et al^40^ reported no significant association between baseline PFAS and changes in waist circumference, total fat mass, fasting glucose, or insulin, but a significant negative association between the fall in visceral fat mass during the diet and each of PFOS, PFNA, and PFDA.

Both papers performed the measurements in the same laboratory, using solid phase extraction followed by liquid chromatography coupled to a triple quadripole mass spectrometer (LC‐MS/MS), which minimizes technical differences and provides high sensitivity and specificity, and baseline measurements prior to the diet were taken. Participants in the POUNDS Lost study were recruited through the mass mailing of the general population^40^ whereas the DioGenes study used a combination of referrals and mass mailing to recruit participants.^41^ Diet interventions were very different and thus difficult to compare: 8 weeks on a complete diet replacement of 800–880 kcal/day^41^ versus a 6‐month 750 kcal deficit compared to estimated requirements.^40^ However, the similarity in PFAS effects on weight regain with different interventions is noteworthy.

### 3.5 Plasticizers and personal care products

Urinary phthalates, bisphenols, and parabens were measured by van der Meer, et al^42^ and their impact on BMI, % fat, and waist circumference was examined as post‐hoc analysis of a large clinical trial in Europe (the LOWER study) consisting of a 3 month restricted calorie intake to 33% energy requirements. For every 1 μg/ml increase in methyl or propyl paraben, there was a predicted 0.1–0.11 increase in BMI, which amounts to a difference in BMI of 13.62 and 3.43 across the reported interquartile range of methyl and ethyl paraben respectively (Table S9). For every 1 μg/ml increase in mono benzyl‐phthalate there was a predicted increase of 0.68 cm in waist circumference and an increase in body fat content by 0.62%, which predicts a difference in waist circumference of 17.5 cm and a difference of 16% body fat across the range of values in the study. These associations remained even when people with T2D were removed from the analysis. There were no associations between BPA and reported mass loss characteristics.^42^

Patients with a BMI of over 27 were referred from a weight loss clinic.^47^ Chemical analysis was performed using LC–MS/MS and baseline measurements prior to the diet were taken. For bisphenols, it is unclear whether this was only the parent compound or included gluconated or sulfonated forms, which represent the metabolites and are present at higher concentrations.^48^ Timing and means of sampling may influence exposures recorded because these chemicals tend to be quickly eliminated and do not bioaccumulate.^48^ Typically, 24 h sampling for urine is more representative than a spot sample for rapidly metabolized compounds, and this approach was used by van der Meer, et al.^42^ However, no precautions to avoid contamination during sampling, storage, or processing, particularly for parabens and bisphenols, were reported, which can be an issue for ubiquitous plastic associated compounds.^48^, ^49^

### 3.6 Air pollutants

Ustulin, et al^43^ reported on a large retrospective cohort study of urban dwelling Noom Coach app users in 2012–2014, initially in cities across Europe, North America, Asia, and Australasia (study 1: *n* = 2608), and then focused on the USA (study 2: *n* = 995). Air pollution exposure was explored in relation to weight loss trajectory and was estimated based on daily location and weather data using information from calibrated reference sampling stations and modeling approaches making the estimates of exposure very imprecise. Values were an average over an extended period spanning the diet intervention of > = 12 months on an unspecified diet provided by the Noom coach app. Only BMI was reported but mass data was also available. Impaired weight loss was associated with the air pollutants PM_2.5_ and PM_10._ ^43^ For every unit increase in PM_2.5_ BMI was increased by 0.085, which predicts a 1.45 difference in BMI between those who experience the lowest and highest reported average values for PM_2.5_ (Table S9). For every unit increase in PM_10_ BMI was increased by 0.043, giving a predicted difference in BMI of 1.42 across the range of reported average values. The significant pollution index by time interaction in the mixed model for US Noom users had a very small co‐efficient (1.5 x 10^−^5) which is orders of magnitude lower than impacts of other variables including mean calorie intake, activity, sex, and frequency of data input. There was no cut‐off BMI for selection, such that large numbers fell in a ‘normal weight’ category.

### 3.7 Accounting for other modifying factors

Known modifying factors for weight loss that were reported and/or included in statistical analysis are provided in Table S10. Ethnicity was not reported in four studies,^39^, ^41^, ^42^ or in previous studies that provide details on participant characteristics.^44^, ^47^, ^50^, ^51^ The ethnicity of participants in Liu, et al^40^ was predominantly white (78%), with some Black (16%), Hispanic (4%), Asian (1%) or Other (1%) ethnic groups.^52^ All participants were reported as ‘Caucasian’ in Imbeault, et al.^38^

Sex balance was reported in all papers and included in the analysis in five. There was a greater proportion of women (62–70%) in the large clinical trials and retrospective study investigating effects of chemical exposure on weight loss.^40^, ^41^, ^42^, ^43^ In the two smaller studies that investigated glycemic control the sex ratio was closer to 50:50.^38^, ^39^

Adults were investigated in all six papers. The upper age limit was 50 in Imbeault, et al^38^ and the average age and range were much lower in Ustulin, et al,^43^ compared to upper ages of 60–70 and broader age ranges in the other four.

Five of the six papers focused on participants with obesity or overweight. DM status of the participants was 19% in the LOWER study,^42^ recorded using a questionnaire. T1D was excluded, suggesting that participants with diabetes therefore had T2D. Diabetes was an exclusion criterion for participation in the POUNDS lost^40^ and DioGenes studies.^45^ Although Type 1 or 2 was not specified in Liu, et al,^40^ previous papers from the same study showed no evidence of high fasting blood glucose or insulin at baseline on average.^52^ DM status was not reported in Ustulin, et al.^43^ Kahleova, et al^39^ was the only paper to deliberately target patients with T2D, who were taking hypoglycemic agents and not insulin.

Assumptions made in the implementation of the interventions and monitoring of compliance can influence observed mass loss trajectory, including energy intake calculations to inform daily intake and multipliers to adjust for physical activity. No adjustments for sex, age, activity, or other metabolic requirements were made to assign the calorie restriction in the DioGenes study.^41^ Far less detail on diet, its caloric content and composition, setting, and compliance monitoring was given in Imbeault, et al^38^ and Ustulin, et al^43^ compared with the other four studies (Table S10; S12). While both Grandjean, et al^41^ and Kahleova, et al^39^ reported how adherence was monitored, neither explained how this information was used in analysis or decisions about ongoing inclusion. Ustulin, et al^43^ used log‐ins to the Noom app as a controlling variable in the analysis, but this is a loose proxy for adherence. The other three papers did not report on compliance.

### 3.8 Risk of bias

**FIGURE 1:**
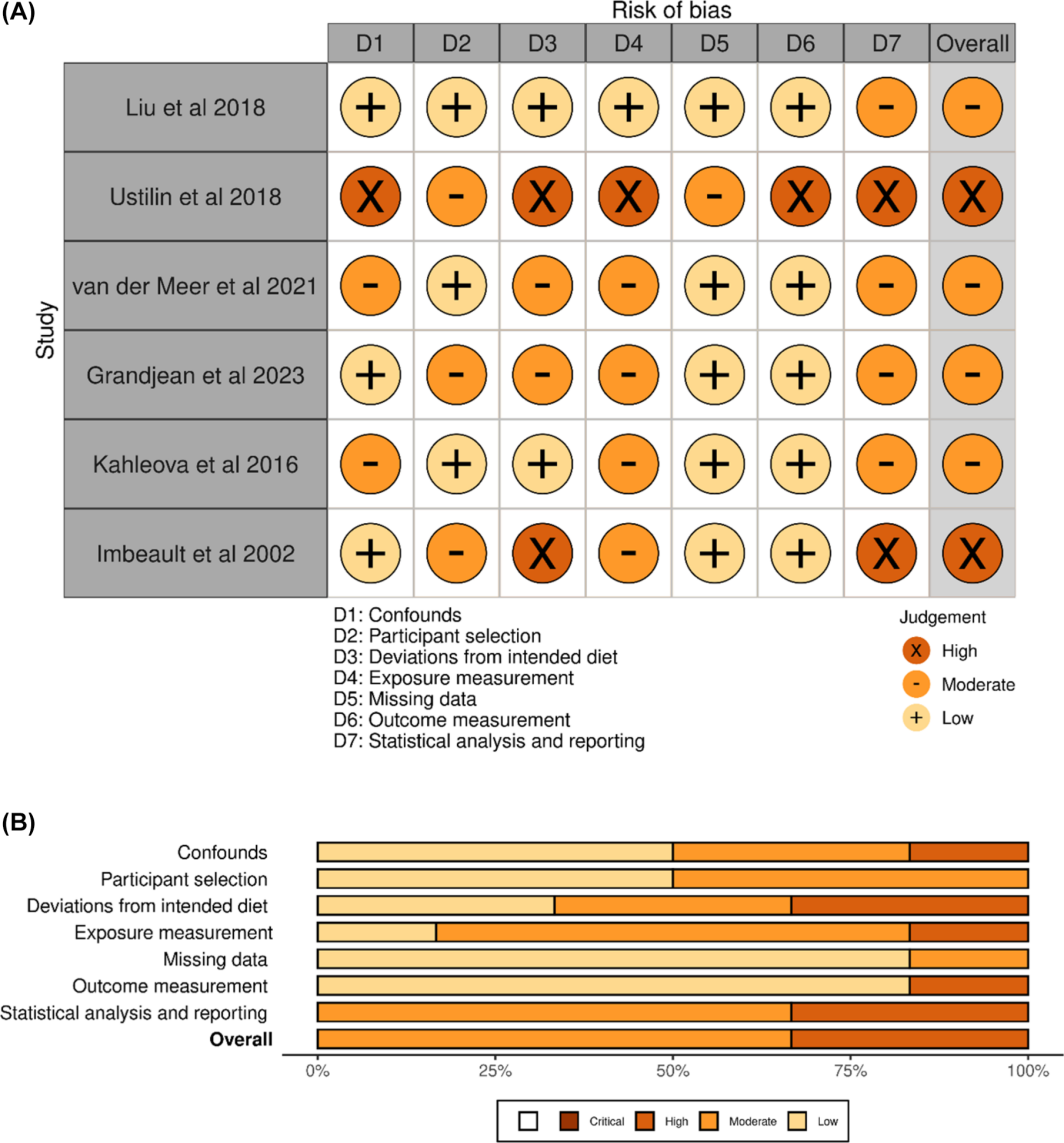
Traffic light (a) and unweighted summary (b) plots for risk of bias in seven domains for the six studies that addressed the hypothesis that contaminant exposure may affect success of weight loss or glycemic control resulting from dietary calorie restriction intervention. Overall risk is the highest rating from any domain: D1 = bias due to confounds; D2 = bias due to participant selection; D3 = bias due to deviations from intended diet; D4 = bias due to exposure measurement; D5 = bias due to missing data; D6 = bias due to outcome measurement; D7 = bias due to reporting and statistical analysis. Dark orange with x = high risk; mid orange with – = moderate risk; pale orange with + = low risk. Summary plot indicates what proportion of studies fall into each risk category for each domain. Produced using RobVis.^37^

RoB is summarized in Figure 1 (details in Table S12; individual evaluation Table S13). The potential for lack of diet adherence, and lack of clarity and completeness in analysis and reporting had the greatest impact across studies. Both Ustulin, et al^43^ and Imbeault, et al^38^ were at high RoB and provided the weakest evidence for the reported associations. Potential issues with snapshot measurement of exposure typical of cross‐sectional studies were highlighted, particularly for chemicals that are rapidly metabolized.^42^ Issues caused by the measurement of chemicals in samples that were not originally intended for their analysis resulted in a moderate risk of bias for most studies. Missing data and outcome measurement were the domains of least concern.

### 3.9 Excluded papers

Despite measuring mass and at least one other aspect of adiposity or glycemic control and relevant chemical exposure, 17 papers did not investigate associations between the chemical of interest and those outcomes (Table S14). Instead, they investigated chemical redistribution during mass loss or other outcomes. Although relevant data exist that may be comparable for up to 10 of these papers on PCBs or OCPs, they cannot be extracted for post hoc analysis because they are typically presented as group averages and do not pair the contaminant values with mass per participant. In addition, in two of the papers, participants who lost weight due to bariatric surgery were combined with those that underwent a dietary intervention,^53^, ^54^ making the effects of contaminants on diet‐induced weight loss impossible to disentangle.

## 4 DISCUSSION

This review highlights the paucity of studies that examine whether contaminants impair diet‐induced weight loss or impact the improvement in glycemic control during weight‐loss. Only six studies explicitly examined the hypothesis that contaminant exposure could impair weight loss or improvement of glycemic control during diet‐induced weight loss. They show that both emerging and legacy contaminants may be associated with detrimental alterations in diet‐induced weight loss or glycemic control. However, participants were recruited at least 12 years ago and, in some cases, almost 20 years ago, such that the information is already substantially outdated in relation to current exposures.

The chemical groups associated with impaired diet‐induced weight loss or altered metabolic rate that may influence weight regain after a diet, were all emerging contaminants of concern that are less lipophilic and typically more easily metabolized and eliminated than legacy POPs. However, PCBs, polybrominated flame retardants, OCPs, glyphosate, or heavy metals were not examined in terms of impacts on diet‐induced weight loss, despite several papers (17) investigating chemical redistribution during weight loss and/or their association with other aspects of physiology.

The lack of consistency in chemicals and outcomes measured made it impossible to undertake a meta‐analysis. Our findings highlight the need for dedicated human studies. Importantly, focusing on people with or at risk of T2D would determine whether knowledge of contaminant exposure status would justify the refinement of weight loss strategies in T2D management. We recommend several steps to improve the evidence base (Table 2).

**TABLE 2:** Recommendations for future work exploring effect of contaminants on weight loss trajectory or glycemic control during weight‐loss diet interventions.

Aspect	Recommendation
Study design	Compare baseline contaminants between those that successfully lose weight during diet intervention with those who do not Compare baseline contaminants in those who have achieved weight loss and either have or have not undergone T2DM remission, controlling for disease duration, age, and renal function Compare weight loss and glycemia control success in matched people from high versus low exposures where exposure is known beforehand Include longer‐term follow‐up periods in both new studies and in post hoc analyses to allow exploration of duration of effects
Participants	Ensure participants are weight stable at outset to avoid any possibility of pre mobilization of chemicals from adipose, where lipophilic contaminants are those of interest Recruit T2DM patients including those with a range of BMI, disease duration, and renal function/degree of polypharmacy, ensuring these aspects are meticulously recorded and paired with weight loss/glycemia control metrics Recruit from populations with ancestry other than historically northern European, including those with higher rates of T2DM and its onset at lower BMI, and with different high‐risk polymorphisms for drug metabolism and for T2DM risk Recruit people with a range of background exposures Consider inclusion of children or adolescents where appropriate
Diet	Ensure target of minimum 5% mass loss and aim for 10% or 10 kg sustained weight loss to observe biologically meaningful change and to ensure glycemia improvement in ‘positive control’ patients. In participants with clinically meaningful weight loss (which can be stratified by extent of weight loss), assess diet by contaminant exposure interaction Have clear diet adherence and completion criteria Consistency in diet assignment Individual ‐based multipliers calculated for metabolic rate adjustments in calorie allowances
Chemicals of interest	Where the study is retrospective and involves plastic‐associated chemicals, ensure appropriate controls and sample blanks were taken at the time of collection to avoid potential artifacts from collection and storage Where possible, design prospective studies to ensure sample collection at baseline is appropriate for chemicals of interest and or future‐proof Standardize chemical measurement method, matrix, and standards to allow comparability between studies. Use baseline values of chemicals not their change during weight loss as independent variable. New studies that reflect current exposures of a range of candidate contaminants Consider non‐targeted analysis to account for widest range of chemicals Account for total body burden for POPs Consider adipose as well as circulating POP levels
Outcomes	Include a wider range of more sensitive and biologically meaningful outcomes than just mass and BMI, which are problematic and poor predictors of total or visceral body fat content. Absolute body fat and visceral fat content as well as RMR, liver fat content, cholesterol, or inflammation markers may be informative. Include glycemia control metrics including HOMA‐IR/β, GTT, and/or Hb1Ac.
Analysis and reporting	Ensure per treatment reporting, including completion data and ensuring clear comparison of contaminant levels at baseline between completers and non‐completers Report diet adherence method and data clearly to allow comparison of contaminant levels in those who did and did not adhere to the diet and any exercise requirements. Include such data in downstream analysis with a clear explanation of how it was incorporated Present anonymized data to allow extraction, such as in data repository in addition to summaries in papers Where possible report correlations between different contaminants, use data reduction methods to perform analyses, and consider effects of chemical group combinations and non‐linear effects, such as g‐computation Clear reporting of statistics including covariate and sensitivity analysis Avoid quantile analysis and instead report dose response to allow meta‐regression across studies with different background exposures Clear reporting of dose‐responses to allow effect sizes to be extracted

### 4.1 Chlorinated POPs

Increased circulating POPs, particularly PCBs, during weight loss was associated with some less favorable glycemic control outcomes, but the changes were not always consistent within and between studies and, by some measures, T2D risk may have improved.^38^, ^39^ Improvement in insulin and/or glucose levels but lack of change in whole‐body insulin sensitivity suggest a complex metabolic and endocrine response to fat loss, elevated circulating POPs, and perhaps increased POP clearance. Preclinical data show impaired glucose tolerance, particularly in males and dependent on AhR, in response to CB77 during calorie restriction in mice but no impact on mass change.^22^, ^23^, ^25^, ^29^ The current tolerable weekly intake (TWI) for PCBs in humans set by the European Food Safety Agency (EFSA) is 2 pg/kg^55^ whereas the mice were gavaged with 50 mg/kg of CB77 weekly. The impacts on glucose regulation were thus seen at dose rates far in excess (10^10^) of recommended human intakes.

CB153 levels, a typical marker for PCB exposure, in Imbeault et al ^38^ were higher than the population geometric mean from NHANES data, a nationwide health survey for people living in the USA, from 2001 to 2002 (mean = 27.2 [24.7–30.1 CI) ng/g lw and 2003–2004 (19.8 [18.8–20.9 CI) ng/g lw around the time of recruitment to the study (https://www.cdc.gov/exposurereport/data_tables.html), which may relate to higher %BF than in the background population. The CB153 values were more comparable to the 50th and 75th percentile values from those years, suggesting they were representative of relatively higher exposures in North America in the early 2000s. Serum levels of CB153 in the participants recruited 8–10 years later in the Czech Republic were considerably lower at baseline and after the diet (1.14–1.29 ng/g at baseline; 1.14–1.43 ng/g)^39^. Indeed, globally, exposure to PCB in the general population has been declining since the 1980s, with the highest levels consistently in North America, particularly in the Canadian Arctic, and North East Europe.^56^ Some populations, such as those living close to former PCB manufacturing sites may continue to have very high exposures (0.1‐1 μg/g lw).^57^ An impact of current PCBs exposures on glycemic control resolution during weight loss cannot be inferred from the available data.

### 4.2 PFAS

PFAS, which are particularly bioactive and persistent, were associated with alterations to RMR, primarily in women,^40^ and with increased weight regain after the weight loss period^40^, ^41^ of a magnitude associated with increased T2D or IGT risk.^7^ These data are consistent with preclinical experiments in mice in which PFOS exposure impaired the beneficial effects of weight loss in improving hepatic lipid accumulation and glycemia.^24^ Nevertheless, the dose rate in the animal study was 100 μg/kg/day, which, although comparable with the no observed adverse effect level (NOAEL) in mice, is over 150,000 times higher than the TWI of 4.4 ng/kg/bw for PFAS in humans.^58^ In the same study, in vitro exposure of HepG2 and 3T3‐L1 adipocytes to PFOS used concentrations of 2.5–25 μM, which are 40,000 to 400,000 times higher than blood levels that produce severe health outcomes in humans. The preclinical data, while offering potential mechanistic insights, is thus of limited relevance to human exposures.

The average exposures in the European participants from 2006 to 2008^41^ were generally about half that seen in the USA study that recruited from 2004 to 2007.^40^ The exposures reported in both papers were generally typical of the background population in North America at the time, based on geometric mean PFOS levels in the NHANES study of 20.7 [19.2–22.3 CI] μg/ L in 2003/ 2004 and 17.1 [16–18.2 CI] μg/ L in 2004/2005 for the whole population. The exposures reported^40^, ^41^ represent all the EFSA risk categories for these four PFAS from low (0·7–9·5 ng/ml), through moderate (>9·5–17·5 ng/ml) and high (>17·5–31·9 ng/ml), to severe (>31·9 ng/ml), and some values in each study were as high as the background population 95th percentile. Even in the lower quartile of participants in Grandjean et al^41^ the summed plasma concentrations of PFOA, PFNA, PFHxS, and PFOS exceeded the values that correspond to the TWI.^58^ Recent global statistics suggest that populations in European countries, North America, the Arctic, and Australia have higher serum PFAS than South America, Africa, and mainland Asia, with typical serum levels in most high‐exposure countries falling in the EFSA moderate and high‐risk categories.^59^ The most recent Centers for Disease Control data, based on the NHANES dataset from 2017–2018,^60^ shows the geometric mean of PFOS in the total population in the USA declined to 4.25 [3.9–4.62 CI] μg/ L with a 95th percentile of 14.6 [13.1–16.4 CI] μg/L, suggesting that a much smaller proportion of the current European and North American populations are now likely to experience the high PFAS levels reported in Grandjean and Liu^40^, ^41^, although the apparent decline in exposure does not account for replacement PFAS chemicals. Populations living near contaminated sites from the manufacture or use of firefighting foam and other PFAS production or waste disposal, those who work in the electroplating industry, are still at risk of high PFAS exposures.^61^ In addition, Greenland Inuit people experience the highest non‐occupational exposures of all high‐exposure nations.^59^ Adjustment for PFAS improved precision of the estimates of impacts of diet intervention after other modifying factors had been taken into account but were not major predictors of weight change trajectory. Now that average PFAS levels are comparable with the lowest quartile from a decade ago in North America and Europe, the importance of PFAS in post‐diet weight maintenance needs to be reassessed.

### 4.3 Plasticizers and personal care products

Parabens and phthalates, but not bisphenols, were associated with clinically relevant impaired fat reduction during weight loss, and some of these associations were dependent on T2D status.^42^ There are no comparable animal studies for these chemical groups.^29^ Comparisons in exposures between this study and typical population values are difficult because interconversion between 24‐hour exposures and μg/L requires more information than presented. Exposure to many of the phthalate and paraben metabolites examined has declined in Europe and the USA since the participants were recruited for this study.^62^, ^63^, ^64^ Children in the Netherlands typically have lower urinary phthalate concentrations than other European nations, and exposures may differ 2–9 times between countries.^65^ The phthalate values here were less than half of those reported from the same period for the general Dutch population, such that values from participants in van der Meer et al^42^ may underestimate risks or fail to represent the range of concentrations experienced across Europe and more broadly. Detection of phthalates in urine samples remains high across Europe (65–100% depending on metabolite) and non‐regulated phthalate substitutes are rising rapidly, by as much as 50–60% annually in some regions.^62^ Phthalate and paraben compounds identified by van der Meer et al^42^ may now have less influence on mass loss, though risks may remain for e‐waste workers and others with higher exposures.^64^ The consequences of exposure to higher phthalate or paraben levels than those found in van der Meer et al, ^42^ or of replacement chemicals that are rapidly increasing (eg 1,2‐cyclohexane dicarboxylic acid diisononyl ester [DINCH]) and may have a similar mode of action, have not been explored in a weight loss context.

### 4.4 Air pollutants

Air pollution, particularly from PM_2.5_ and PM_10_, and possibly ozone, was reported to curtail the fall in BMI seen in Noom Coach app users over a 12‐month period,^43^ but the effect size was not biologically meaningful, such that air pollution seems unlikely to be a major modifying factor in weight loss. However, studies that better track individual exposure and identify its chemical constituents would be necessary to explore this possibility. There are no comparable data from animal studies for air pollutants.^29^

### 4.5 Limitations and missed opportunities with available evidence

The reliance on post hoc analysis weakened the conclusions because the study population was not recruited with the analysis of contaminant effects on weight loss in mind. Only one study intended to examine the impact of chemical exposure on glycemia control from the outset and yet was limited in its ability to do so by reporting ΔPOPs rather than baseline levels.^39^ Procedural blanks, could not always be put in place at the time of sampling for unanticipated post hoc analyses,^40^, ^41^ such that degradation, leaching during processing or storage, or adsorption to surfaces during sampling and storage were not accounted for.^49^, ^66^ Lack of directness, precision, and accuracy in air pollution exposure estimates substantially impacted the quality of evidence for the effects of PM _2.5_ and PM_10._ ^43^ For POPs, plasma or serum levels may not reflect concentrations in important pools, such as adipose or liver, where local metabolic disruption may occur. Accounting for initial total body burden as well as circulating concentrations for lipophilic contaminants would allow causal relationships to be better examined. In all cases, other unmeasured correlates of exposure could be responsible for observed associations.

No studies compare exposure between people with differing weight loss trajectories, or between glycaemia responders and non‐responders. Stratification by the degree of weight loss is vital because measurable effects on glycemic control begin with >5% mass loss, and 80–90% of people who lose >10% of fat mass have significant glycemic improvement and high success in T2D remission.^1^, ^2^, ^3^

No studies exist from high occupational exposure settings or in populations with high exposure from industrial accidents, localized food contamination, residence close to contaminated sites or high incidental contaminant intake^59^, ^64^ Only Ustulin, et al^43^ included people from Asia and Australasia. There are no studies in populations with a higher prevalence of obesity or T2D^67^, ^68^, or in populations where T2D onset occurs at much lower BMI and with different high‐risk genes for T2D and detoxification enzyme polymorphisms than in white Europeans^21^, ^69^, ^70^.

Few studies actively recruited patients with T2D and several specifically excluded this group. Co‐existence of renal disease in people with T2D,^71^ may impact absorption, metabolism, and clearance of contaminants and thus lead to higher or more prolonged exposure that could exacerbate metabolic consequences. Drugs taken by people with T2D^72^ may interact with contaminants and alter responses.^24^

With obesity increasing in the under 16 s,^73^ combined with higher susceptibility of young people to damaging effects of contaminants,^74^ and exposures linked to obesity and impaired glucose metabolism^75^ such studies should be considered.

The ability of proxies, such as mass, BMI, and WC, to adequately represent body fat content, visceral fat, or other metabolic health parameters is of concern, especially when self‐reported. Detection of biologically important changes may be limited, despite their common use as surrogate adiposity measures^76^ and use of more sensitive markers is warranted.

All diets produced some improvement in glycemic control, when these indices were measured, although it is not always clear if these were clinically as well as statistically significant. Three of the diets were not extreme weight loss diets,^40^, ^43^, ^44^ and thus if the effects of contaminants on mass loss are subtle, the modest weight loss achieved may have prevented any contaminant effects from being detectable. Grandjean, et al^41^ specifically excluded those who did not lose 8% or more of their initial mass, which may have precluded the detection of an effect of PFAS during the low‐calorie phase of their intervention.

The use of a single assumed multiplier for caloric intake in four studies may have introduced apparent sex differences.^77^ Lack of reliability in compliance reporting and monitoring could have affected inter‐subject variability, and thus the ability to detect an effect when one was present.^78^ People with higher contaminant levels could be less able to comply due to altered appetite^26^.

The absence of study protocols, analysis plans, and the use of reporting checklists in several instances also made it hard to resolve whether the evidence gap we have identified is a result of publication bias. Three post hoc studies used a per protocol analysis and did not consider those for whom the data were not available from the original study. This approach can produce bias since drop out could be in some way associated with the chemical exposure or outcomes of interest. In addition, Kahleova, et al^39^ report the link between the change in POPs and glycemia control as a serendipitous finding that was not originally planned even in the post hoc analysis, an approach that is vulnerable to p‐hacking.

There is no obvious reason the data were grouped in tertiles of exposure^40^ or weight loss.^41^ Quantile analysis, particularly when data are placed into few groups,^79^ can be arbitrary, oversimplify the data and lose information, fail to detect non‐linear trends, and depends on the range of values experienced by the population in question, making between study comparisons a challenge, and can mask outliers that have large effects on the results. A dose–response estimate would eliminate such issues and identify non‐linear effects. Grandjean, et al^41^ attempted to overcome this issue by using g‐computation, which can overcome linearity assumptions.^46^

Modifying effects of established clinical parameters that can influence weight loss trajectory and/or contaminant burden were not always well controlled for, making it challenging to determine the relative importance of the pollutants assessed in altering weight loss trajectory or glycemia resolution.

### 4.6 Study strengths and weaknesses

Our broad scope allowed us to capture a range of study designs and identify papers from a wide range of chemicals. We may have unintentionally omitted some chemical groups that have important impacts because they have not been investigated in the context of T2D or obesity, or because we did not search gray literature or reports not written in English. However, it is unlikely that a chemical that has not been previously linked with metabolic disturbance in humans would be targeted in weight loss studies.

The search strategy did not specifically target studies on weight regain after diet‐induced weight loss although some were identified. Knowing the impact of chemical exposure on longer‐term weight maintenance would have additional clinical benefits. We focused only on weight loss metrics and on measures of glycemic control. Other parameters such as cholesterol, thyroid hormones, adipokines, inflammatory markers, and metabolic rate measured in several of the studies may be more impacted than the outcomes explored here. In addition, we did not include other important comorbidities that can be resolved during weight loss, such as cardiovascular risk.

## 5 CONCLUSIONS

Despite burgeoning literature linking chemical exposure to the dual epidemics of T2D and obesity, our review highlights the paucity of information on the impact of diabetogenic and obesogenic chemicals on weight loss in humans, a key tool in the prevention and remission of T2D. There is no clear consensus on the effects of the small number of chemical groups that have been investigated. The impact of POPs on glycemic control is not consistent and suggests a complex relationship with metabolism that is hard to disentangle from fat loss. The suggested role of parabens, phthalates, and air pollutants in slowing fat loss, and for PFAS in suppressing RMR during diet‐induced weight loss need to be replicated across contexts and populations to increase confidence and establish clinical relevance relative to other modifiers of weight loss trajectory and in light of current exposures. The current limited evidence is hampered, particularly by the potential for both lack of adherence to diet, selective reporting, and because many analyses are post hoc. Furthermore, temporality may be a major confound. Reverse causation or the possibility that unmeasured chemical groups or other modifying factors may be responsible for observed effects or mask important impacts cannot be excluded. Our recommendations to explore and strengthen existing evidence (Table 2) include: determining which chemical groups and mixtures are of greatest concern; better recording, reporting, and inclusion in analysis of compliance with diet and exercise regimes; improved reporting of study protocol and statistical analysis plans to remove doubt over publication bias; and a greater focus on people with T2D or pre‐diabetes.

There are exciting opportunities to perform these analyses on real‐world populations as many countries now have weight loss interventions specifically linked to T2D prevention and remission within their health systems. Encouraging health care collection of the key data discussed above with ethics and governance to allow research would enable these studies to be conducted quickly on a large scale but may require contaminant analysis to be built into routine practice. Knowing the precise impact of environmental chemicals on weight loss, a major strategy in T2D prevention and remission, is necessary to determine if chemical screening could improve weight loss management.

## AUTHOR CONTRIBUTIONS

Conceptualization, formal analysis, investigation: KB, ALS; Methodology, validation, resources, original draft, review, and editing: KB, ALS, CS; Software, data curation, visualization, administration: KB.

## ETHICS APPROVAL

This study is a systematic review of data already anonymized and available in the public domain. Expedited approval has been granted for this work by the Abertay University ethics committee (reference EMIS 5911).

## Supporting information


**Table S1:** Search results for systematic reviews of the association between obesity, diabetes, or metabolic syndrome and chemical exposure from within the last 5 years from Pubmed.
**Table S2**: Search strategy for the current systematic review.
**Table S3**: Signal questions and the criteria for overall risk of bias rating for each of seven domains considered.
**Figure S4**: PRISMA 2020 flow diagram showing outcome of searches of databases and other sources, screening, and final inclusion of papers and studies.
**Figure S5**: Venn diagram of sources of total hits and included papers.
**Table S6**: Details of the characteristics of the six identified studies that assessed impact of contaminants on mass loss or glycemic control parameters.
**Figure S7**: Timeline of recruitment to the different studies according to the chemical class examined
**Figure S8**: Sankey chart of information flow in the current evidence base.
**Table S9**: Detailed reporting findings from each of the six studies identified in the systematic review.
**Table S10:** Confounds and covariates reported and accounted for in analysis 2.
**Table S11**: PCB congeners analyzed and methodological considerations.
**Table S12**: Details of consensus RoB assessment.
**Table S13**: Risk of bias assessment by each evaluator and consensus.
**Table S14**: Study characteristics of 17 papers excluded because they did not assess contaminant effects on outcome of interest despite measuring both, or pooled information from bariatric surgery with diet.

## Data Availability

The datasets generated during the current study are available in Nested Knowledge, and in the Open Science Framework, https://osf.io/ckjpt/?view_only=af2a04807edb4cfdaba759a2e135e584 in addition to the supplementary files provided.
