# Supplementary material for: A systematic review of evidence that environmental contaminant exposure impedes weight loss and glycemic control during calorie‐restricted diets in humans
Source: Obes Rev. 2024 Dec 23;26(5):e13886. doi: 10.1111/obr.13886 (PMC11964800; doi:10.1111/obr.13886)
Supplement: Supplementary file 1 — Table S1: Search results for systematic reviews of the association between obesity, diabetes, or metabolic syndrome and chemical exposure from within the last 5 years from Pubmed. Table S2: Search strategy for the current systematic review. Table S3: Signal questions and the criteria for overall risk of bias rating for each of seven domains considered. Figure S4: PRISMA 2020 flow diagram showing outcome of searches of databases and other sources, screening, and final inclusion of papers and studies. Figure S5: Venn diagram of sources of total hits and included papers. Table S6: Details of the characteristics of the six identified studies that assessed impact of contaminants on mass loss or glycemic control parameters. Figure S7: Timeline of recruitment to the different studies according to the chemical class examined Figure S8: Sankey chart of information flow in the current evidence base. Table S9: Detailed reporting findings from each of the six studies identified in the systematic review. Table S10: Confounds and covariates reported and accounted for in analysis 2. Table S11: PCB congeners analyzed and methodological considerations. Table S12: Details of consensus RoB assessment. Table S13: Risk of bias assessment by each evaluator and consensus. Table S14: Study characteristics of 17 papers excluded because they did not assess contaminant effects on outcome of interest despite measuring both, or pooled information from bariatric surgery with diet. [file OBR-26-e13886-s001.pdf]

Supplementary material for:

A systematic review of evidence that environmental contaminant exposure impedes weight loss and glycaemic control during calorie-restricted diets in humans.

Bennett, K.A[1*] ORCID ID: 0000-0003-2168-6112; Sutherland C[2]. ORCID ID: 0000-0003-4398-7434, Savage A.L[1]: ORCID ID: 0000-0002-2022-1893.

This file contains:

Table S1: Search results for systematic reviews of the association between obesity, diabetes or metabolic syndrome and chemical exposure from within the last 5 years from Pubmed

Table S2: Search strategy for the current systematic review

Table S3: Signal questions and the criteria for overall risk of bias rating for each of seven domains considered

Figure S4: PRISMA 2020 flow diagram showing outcome of searches of databases and other sources, screening and final inclusion of papers and studies.

Figure S5: Venn diagram of sources of total hits and included papers

Table S6: Details of the characteristics of the six identified studies that assessed impact of contaminants on mass loss or glycaemic control parameters

Figure S7: Timeline of recruitment to the different studies according to chemical class examined

Figure S8: Sankey chart of information flow in current evidence base

Table S9: Detailed reporting findings from each of the six studies identified in the systematic review

Table S10: Confounds and covariates reported and accounted for in analysis

Table S11: PCB congeners analysed and methodological considerations

Table S12: Details of consensus RoB assessment

Table S13: Risk of bias assessment by each evaluator and consensus

Table S14: Study characteristics of 17 papers excluded because they did not assess contaminant effects on outcome of interest despite measuring both, or pooled information from bariatric surgery with diet

Table S1: Systematic reviews and umbrella reviews from within the last 5 years retrieved from Pubmed (Nov 2024) that examine the evidence for increased risk of obesity or diabetes with chemical exposure

Condition	Chemical class	Reference
Obesity	Phthalates	Wu Q, Li G, Zhao CY, Na XL, Zhang YB. Association between phthalate exposure and obesity risk: A meta-analysis of observational studies. *Environ Toxicol Pharmacol*. 2023. 102:104240. doi: 10.1016/j.etap.2023.104240.<br>Guo J, Liu K, Yang J, Su Y. The association between prenatal exposure to bisphenol A and offspring obesity: a systematic review. *Environ Pollut*. 2024 344: 123391. doi: 10.1016/j.envpol.2024.123391.<br>Lee DW, Lim HM, Lee JY, et al. Prenatal exposure to phthalate and decreased body mass index of children: a systematic review and meta-analysis. *Sci Rep.* 2022. 12: 8961. doi: 10.1038/s41598-022-13154-9.
	BPA	Wu W, Li M, Liu A, et al. Bisphenol A and the risk of obesity a systematic review with meta-analysis of the epidemiological evidence. *Dose Response*. 2020 18:1559325820916949. doi: 10.1177/1559325820916949.<br>Ribeiro CM, Beserra BTS, Silva NG et al. Exposure to endocrine-disrupting chemicals and anthropometric measures of obesity: a systematic review and meta-analysis. *BMJ Open*. 2020. 10:e033509. doi: 10.1136/bmjopen-2019-033509.
	Metals	Zangiabadian M, Jolfayi AG, Nejadghaderi SA, Amirkhosravi L, Sanjari M. The association between heavy metal exposure and obesity: A systematic review and meta-analysis. *J Diabetes Metab Disord.* 2023. 23: 11-26. doi: 10.1007/s40200-023-01307-0.
	Polyaromatic hydrocarbons	Liu C, Liu Q, Song S et al. The association between internal polycyclic aromatic hydrocarbons exposure and risk of obesity-a systematic review with meta-analysis. *Chemosphere*. 2023. 329:138669. doi: 10.1016/j.chemosphere.2023.138669.
	Organochlorines	Stratakis N, Rock S, La Merrill MA et al. Prenatal exposure to persistent organic pollutants and childhood obesity: A systematic review and meta-analysis of human studies. *Obes Rev.* 2022. 23 Suppl 1:e13383. doi: 10.1111/obr.13383.<br>Ribeiro CM, Beserra BTS, Silva NG et al. Exposure to endocrine-disrupting chemicals and anthropometric measures of obesity: a systematic review and meta-analysis. *BMJ Open*. 2020. 10:e033509. doi: 10.1136/bmjopen-2019-033509.
		Tamayo-Ortiz M, Téllez-Rojo MM, Rothenberg SJ et al. Exposure to $PM_{2.5}$ and Obesity prevalence in the Greater

		Mexico City area. *Int J Environ Res Public Health*. 2021. 18:2301. doi: 10.3390/ijerph18052301.<br>Huang S, Zhang X, Huang J, Lu X, Liu F, Gu D. Ambient air pollution and body weight status in adults: A systematic review and meta-analysis. *Environ Pollut*. 2020. 265:114999. doi: 10.1016/j.envpol.2020.114999.<br>Huang C, Li C, Zhao F, Zhu J, Wang S, Sun G. The association between childhood exposure to ambient air pollution and obesity: a systematic review and meta-analysis. *Int J Environ Res Public Health.* 2022. 19:4491. doi: 10.3390/ijerph19084491.
	Particulate and chemical air pollutants	Parasin N, Amnuaylojaroen T, Saokaew S. Effect of air pollution on obesity in children: a systematic review and meta-analysis. *Children* 2021. 8:327. doi: 10.3390/children8050327.<br>Zheng J, Zhang H, Shi J. Association of air pollution exposure with overweight or obesity in children and adolescents: A systematic review and meta-analysis. *Sci Total Environ.* 2024 910: 168589. doi: 10.1016/j.scitotenv.2023.168589.
		Luo C, Wei T, Jiang W et al. The association between air pollution and obesity: an umbrella review of meta-analyses and systematic reviews. *BMC Public Health.* 2024 Jul 11;24(1):1856. doi: 10.1186/s12889-024-19370-4.
		Lin L, Li T, Sun M. Global association between atmospheric particulate matter and obesity: A systematic review and meta-analysis. *Environ Res*. 2022. 209:112785. doi: 10.1016/j.envres.2022.112785.
		Siewert B, Kozajda A, Jaskulak M, Zorena K. Examining the link between air quality (PM, SO_2, NO_2, PAHs) and childhood obesity: a systematic review. *J Clin Med*. 2024. 13:5605. doi: 10.3390/jcm13185605.
	PFAS	Frigerio G, Ferrari CM, Fustinoni S. Prenatal and childhood exposure to per-/polyfluoroalkyl substances (PFASs) and its associations with childhood overweight and/or obesity: a systematic review with meta-analyses. *Environ Health.* 2023. 22: 56. doi: 10.1186/s12940-023-01006-6.<br>Frangione B, Birk S, Benzouak T et al. Exposure to perfluoroalkyl and polyfluoroalkyl substances and pediatric obesity: a systematic review and meta-analysis. *Int J Obes* 2024 48:131-146. doi: 10.1038/s41366-023-01401-6.
	Pesticides	Pinos H, Carrillo B, Merchán A et al. Relationship between prenatal or postnatal exposure to pesticides and obesity: a systematic review. *Int J Environ Res Public Health.* 2021. 18:7170. doi: 10.3390/ijerph18137170.
Metabolic syndrome	phthalates	Perez-Diaz C, Uriz-Martínez M, Ortega-Rico C et al. Phthalate exposure and risk of metabolic syndrome components: A

		systematic review. *Environ Pollut*. 2024 340:122714. doi: 10.1016/j.envpol.2023.122714.
	BPA	Xiao T, Huang Z, Zheng C et al. Associations of bisphenol A exposure with metabolic syndrome and its components: A systematic review and meta-analysis. *Obes Rev*. 2024 25:e13738. doi: 10.1111/obr.13738.
	PCBs and dioxins	Mohd Efendy Goon MD, Zulkifli S, Abdullah Soheimi SS et al. Association between polychlorinated biphenyl (PCB) and dioxin with metabolic syndrome (METS): a systematic review and meta-analysis. *Sci Rep*. 2024 14:17941. doi: 10.1038/s41598-024-68369-9.
Prediabetes and/ or T2DM	Metals	Filippini T, Wise LA, Vinceti M. Cadmium exposure and risk of diabetes and prediabetes: A systematic review and dose-response meta-analysis. *Environ Int*. 2022. 158:106920. doi: 10.1016/j.envint.2021.106920.<br>Rahimi Kakavandi N, Mousavi T, Asadi T et al. An updated systematic review and dose-response meta-analysis on the relation between exposure to arsenic and risk of type 2 diabetes. *Toxicol Lett*. 2023. 384:115-127. doi: 10.1016/j.toxlet.2023.08.001.<br>Ghorbani Nejad B, Raeisi T, Janmohammadi P. Mercury exposure and risk of type 2 diabetes: a systematic review and meta-analysis. *Int J Clin Pract.* 2022:7640227. doi: 10.1155/2022/7640227.<br>Guo Y, Lv Y, Liu X, Wang G. Association between heavy metal mercury in body fluids and tissues and diabetes mellitus: a systematic review and meta-analysis. 2023. *Ann Transl Med.* 11: 114. doi: 10.21037/atm-22-6404.
	Particulate and chemical air pollutants	Yu S, Zhang M, Zhu J et al. The effect of ambient ozone exposure on three types of diabetes: a meta-analysis. *Environ Health*. 2023. 22:32. doi: 10.1186/s12940-023-00981-0.<br>Chen ZH, Zhao Z, Deng CW, Li NS.. Association between air pollution and type 2 diabetes mellitus in developing countries: a systematic review and meta-analysis. *Chin Med Sci J.* 2022 37:218-227. doi: 10.24920/004065.<br>Kutlar Joss M, Boogaard H, Samoli E et al. Long-term exposure to traffic-related air pollution and diabetes: a systematic review and meta-analysis. *Int J Public Health*. 2023 68: 1605718. doi: 10.3389/ijph.2023.1605718.
		Yang BY, Fan S, Thiering E, et al. Ambient air pollution and diabetes: A systematic review and meta-analysis. *Environ Res.* 2020 180:108817. doi: 10.1016/j.envres.2019.108817.<br>Yang M, Cheng H, Shen C et al. Effects of long-term exposure to air pollution on the incidence of type 2 diabetes mellitus: a meta-analysis of cohort studies. 2020. *Environ Sci Pollut Res Int.* 27:798-811. doi: 10.1007/s11356-019-06824-1.

	Pyrethroids and organochlorine pesticides	Chen Y, Deng Y, Wu M et al. Impact of pesticides exposure and type 2 diabetes risk: a systematic review and meta-analysis. *Endocrine.* 2024 doi: 10.1007/s12020-024-04067-w.<br>Hernández-Mariano JÁ, Baltazar-Reyes MC, Salazar-Martínez E, Cupul-Uicab LA. Exposure to the pesticide DDT and risk of diabetes and hypertension: systematic review and meta-analysis of prospective studies. *Int J Hyg Environ Health*. 2022. 239:113865. doi: 10.1016/j.ijheh.2021.113865.<br>Yipei Y, Zhilin L, Yuhong L et al. Assessing the risk of diabetes in participants with DDT DDE exposure- a systematic review and meta-analysis. *Environ Res.* 2022. 210:113018. doi: 10.1016/j.envres.2022.113018.<br>Mendes V, Ribeiro C, Delgado I, et al. The association between environmental exposures to chlordanes, adiposity and diabetes-related features: a systematic review and meta-analysis. *Sci Rep*. 2021 11:14546. doi: 10.1038/s41598-021-93868-4.
	PFAS	Gui SY, Qiao JC, Xu KX, et al. Association between per- and polyfluoroalkyl substances exposure and risk of diabetes: a systematic review and meta-analysis. *J Expo Sci Environ Epidemiol.* 2023. 33:40-55. doi: 10.1038/s41370-022-00464-3.
	Plastic associated chemicals	Symeonides C, Aromataris E, Mulders Y et al. An umbrella review of meta-analyses evaluating associations between human health and exposure to major classes of plastic-associated chemicals. *Ann Glob Health*. 2024. 90:52. doi: 10.5334/aogh.4459.
	Phthalates	Radke EG, Galizia A, Thayer KA, Cooper GS. Phthalate exposure and metabolic effects: a systematic review of the human epidemiological evidence. *Env Int* 2019.132: 104768: doi: https://doi.org/10.1016/j.envint.2019.04.040
		Zhang H, Ben Y, Han Y, Zhang Y, Li Y, Chen X. Phthalate exposure and risk of diabetes mellitus: Implications from a systematic review and meta-analysis. *Environ Res.* 2022. 204:112109. doi: 10.1016/j.envres.2021.112109.
	Polyaromatic hydrocarbons	Wang X, Li A, Xu Q. The association between urinary polycyclic aromatic hydrocarbons metabolites and type 2 diabetes mellitus. *Int J Environ Res Public Health*. 2022. 19:7605. doi: 10.3390/ijerph19137605.
		Khosravipour M, Khosravipour H. The association between urinary metabolites of polycyclic aromatic hydrocarbons and diabetes: A systematic review and meta-analysis study. *Chemosphere*. 2020. 247:125680. doi: 10.1016/j.chemosphere.2019.125680.
Gestational diabetes	EDCs in general	Yan D, Jiao Y, Yan H, Liu T, Yan H, Yuan J. Endocrine-disrupting chemicals and the risk of gestational diabetes

	mellitus: a systematic review and meta-analysis. *Environ Health.* 2022. 21:53. doi: 10.1186/s12940-022-00858-8.<br>Yao X, Geng S, Zhu L, Jiang H, Wen J. Environmental pollutants exposure and gestational diabetes mellitus: evidence from epidemiological and experimental studies. *Chemosphere.* 2023 332:138866. doi: 10.1016/j.chemosphere.2023.138866.
POPs in general	Kouiti M, Castillo-Hermoso MÁ, Youlyouz-Marfak I et al. Persistent organic pollutant exposure as a risk factor of gestational diabetes mellitus: A systematic review and meta-analysis. *BJOG*. 2024 131:579-588. doi: 10.1111/1471-0528.17725.
PCBs and PBDEs	Ma J, Li Y, Qian L, et al. Serum levels of polychlorinated biphenyls and polybrominated diphenyl ethers in early pregnancy and their associations with gestational diabetes mellitus. *Chemosphere.* 2023. 339:139640. doi: 10.1016/j.chemosphere.2023.139640.
PFAS	Wang J, Zhang J, Fan Y, et al. Association between per- and polyfluoroalkyl substances and risk of gestational diabetes mellitus. *Int J Hyg Environ Health.* 2022. 240:113904. doi: 10.1016/j.ijheh.2021.113904.
Metals	Wu R, Duan M, Zong D, Li Z. Effect of arsenic on the risk of gestational diabetes mellitus: a systematic review and meta-analysis. *BMC Public Health*. 2024. 24:1131. doi: 10.1186/s12889-024-18596-6.<br>Salmeri N, Villanacci R, Ottolina J, et al. Maternal arsenic exposure and gestational diabetes: a systematic review and meta-analysis. *Nutrients*. 2020. 12:3094. doi: 10.3390/nu12103094.<br>Zhou M, Peng L, Wang J, Cao R, Ou Z, Fang Y. Cadmium exposure and the risk of GDM: evidence emerging from the systematic review and meta-analysis. *Environ Sci Pollut Res Int.* 2022. 29:77253-77274. doi: 10.1007/s11356-022-21171-4.
	Lin Y, Li T, Xiao J, Xie K, Shi Z. The association between cadmium exposure and gestational diabetes mellitus: a systematic review and meta-analysis. *Front Public Health.* 2022. 9:555539. doi: 10.3389/fpubh.2021.555539.
Air pollutants	Zhou X, Li C, Cheng H, Xie J, Li F, Wang L, Ding R. Association between ambient air pollution exposure during pregnancy and gestational diabetes mellitus: a meta-analysis of cohort studies. *Environ Sci Pollut Res Int.* 2022. 29:68615-68635. doi: 10.1007/s11356-022-20594-3.<br>Liang W, Zhu H, Xu J. Ambient air pollution and gestational diabetes mellitus: An updated systematic review and meta-analysis. *Ecotoxicol Environ Saf* 2023. 255:114802. doi: 10.1016/j.ecoenv.2023.114802.

Ren Z, Yuan J, Luo Y, Wang J, Li Y. Association of air pollution and fine particulate matter (PM2.5) exposure with gestational diabetes: a systematic review and meta-analysis. *Ann Transl Med*. 2023. 11:23. doi: 10.21037/atm-22-6306.
Tang X, Zhou JB, Luo F. Air pollution and gestational diabetes mellitus: evidence from cohort studies. *BMJ Open Diabetes Res Care.* 2020. 8: e000937. doi: 10.1136/bmjdrc-2019-000937.
Alvarado-Jiménez D, Donzelli G, Morales-Suárez-Varela M. A systematic review on the association between exposure to air particulate matter during pregnancy and the development of hypertensive disorders of pregnancy and gestational diabetes mellitus. *Rev Environ Health.* 2023. 4. doi: 10.1515/reveh-2022-0258.
Nazarpour S, Ramezani Tehrani F, Valizadeh R, Amiri M. The relationship between air pollutants and gestational diabetes: an updated systematic review and meta-analysis. *J Endocrinol Invest.* 2023. 46:1317-1332. doi: 10.1007/s40618-023-02037-z.
Zhang H, Wang Q, He S et al. Ambient air pollution and gestational diabetes mellitus: A review of evidence from biological mechanisms to population epidemiology. *Sci Total Environ.* 2020. 719:137349. doi: 10.1016/j.scitotenv.2020.137349.

Table S2: Search strategy and terms for the three databases used in this review

Operator	Concept	Search terms
		Pubmed
	Dietary induced, intentional weight loss in people or animals	("Weight Reduction Programs"[Mesh] OR "Diet, Reducing"[Mesh] OR "Diet, Fat-Restricted" [Mesh] OR "Diet, Carbohydrate-Restricted"[Mesh] OR intentional [tiab] OR "dietary intervention" [tiab] OR "caloric restriction" [tiab] OR "calorie restriction" [tiab] OR "meal replacement" [tiab] OR diet n1 restrict* [tiab])
AND	Chemical exposure	("Hydrocarbons, Chlorinated "[Mesh] OR "Hydrocarbons, Brominated "[Mesh] OR "Hydrocarbons, Fluorinated"[Mesh] OR "Hydrocarbons, Halogenated "[Mesh] OR "Dioxins and Dioxin-like Compounds "[Mesh] OR "Halogenated Diphenyl Ethers "[Mesh] OR "Persistent Organic Pollutants "[Mesh] OR "Pesticides" [Mesh] OR "Dibutyl Phthalate"[Mesh] OR "Diethylhexyl Phthalate "[Mesh] OR "Organotin Compounds "[Mesh] OR "Parabens "[Mesh] OR "Neonicotinoids" [Mesh] OR "polychlorinated biphenyl*" [tiab] OR "persistent organic pollutant*" [tiab] OR Aroclor [tiab] OR dioxin* [tiab] OR TCDD [tiab] OR "polybrominated diphenyl ether*" [tiab] OR "polybrominated diethyl ether*" [tiab] OR PBDE* [tiab] OR organochlorine* [tiab] OR DDT [tiab] OR dichlorodiphenyltrichloroethane [tiab] OR "dichlorodiphenyl trichloroethane" [tiab] OR DDE [tiab] OR dichlorodiphenyldichloroethylene [tiab] OR "dichlorodiphenyl dichloroethylene" [tiab] OR heptachlor [tiab] OR nonachlor [tiab] OR HCB [tiab] OR hexachlorobenzene [tiab] OR pyrethroid [tiab] OR "phenoxybenzoic acid " [tiab] OR aldrin [tiab] OR chlordan* [tiab] OR dichlorvos [tiab] OR mevinphos [tiab] OR trichlorfon [tiab] OR alachlor [tiab] OR cyanazine [tiab] OR dieldrin [tiab] OR endrin [tiab] OR hexachlorocyclohexane [tiab] OR endosulfan [tiab] OR PFAS [tiab] OR PFOS [tiab] OR "perfluorooctane sulfonic acid" [tiab] OR PFOA [tiab] OR "perfluorooctanoic acid" [tiab] OR phthalate* [tiab] OR MEHP [tiab] OR DEHP [tiab] OR bisphenol* [tiab] OR BPA [tiab] OR arsenic [tiab] OR cadmium [tiab] OR mercury [tiab] OR tributyltin [tiab] OR organotin [tiab] OR triphenyltin [tiab] OR triclocarban [tiab] OR triclosan [tiab] OR paraben* [tiab] OR carbamate [tiab] OR aldicarb [tiab] OR carbaryl [tiab] OR furan* [tiab] OR neonicotinoid* [tiab] OR imidacloprid [tiab] OR acetamiprid [tiab] OR dinotefuran [tiab] OR thiamethoxam [tiab] OR clothianidin [tiab] OR fipronil [tiab] OR PM2.5 [tiab] OR PM10 [tiab] OR particulate [tiab] OR ozone [tiab] OR "nitrogen oxide"[tiab] OR "nitrogen dioxide" [tiab] OR "sulphur dioxide" [tiab] OR "sulfur

		dioxide"[tiab] OR "carbon monoxide" [tiab] OR "black carbon" [tiab] OR "polyaromatic hydrocarbon" [tiab] OR "PAH"[tiab])
AND	Weight loss outcomes	("weight loss" [tiab] OR "mass loss" [tiab] OR "weight reduc*"[tiab] OR "mass reduc*"[tiab] OR weight n1 reduc* [tiab] OR "waist circumference" [tiab] OR "waist-hip ratio" [tiab] OR "waist to hip ratio" [tiab] OR BMI [tiab] OR "body mass index" [tiab] OR "body fat" [tiab] OR "adiposity" [tiab] OR "fat reduc*"[tiab] OR "fat loss"[tiab])
NOT	Bariatric surgery	("bariatric surgery"[Mesh] OR "bariatric surgery" [tiab] OR "bariatric procedur*" [tiab] "Roux-en-Y " [tiab] OR "gastric band*" [tiab] OR "sleeve gastrectomy" [tiab] OR "gastric bypass" [tiab] OR "gastroplasty" [tiab] OR "jejunoileal bypass" [tiab] OR "stomach stapl*" [tiab])
		Web of Science
	Dietary induced, intentional weight loss in people or animals	TS= ("Weight Reduction Program*" OR "Reducing Diet" OR "Fat-Restricted Diet" OR "Carbohydrate-Restricted Diet" OR intentional OR "dietary intervention" OR "caloric restriction" OR "calorie restriction" OR "meal replacement" OR diet near/1 restrict*)
AND	Chemical exposure	TS = ("Chlorinated Hydrocarbon*" OR "Brominated Hydrocarbon*" OR "Fluorinated Hydrocarbon*" OR "Halogenated Hydrocarbon*" OR Dioxin OR "Dioxin-like" OR "Halogenated Diphenyl Ethers" OR "Persistent Organic Pollutant*" OR "Pesticide" OR "Dibutyl Phthalate" OR "Diethylhexyl Phthalate" OR "Organotin Compound" OR "Parabens" OR "polychlorinated biphenyl*" OR "polyaromatic hydrocarbon*" OR Aroclor OR TCDD OR "polybrominated diphenyl ether*" OR "polybrominated diethyl ether*" OR PBDE* OR organochlorine* OR DDT OR dichlorodiphenyltrichloroethane OR "dichlorodiphenyl trichloroethane" OR DDE OR dichlorodiphenyldichloroethylene OR "dichlorodiphenyl dichloroethylene" OR heptachlor OR nonachlor OR HCB OR hexachlorobenzene OR pyrethroid OR "phenoxybenzoic acid" OR aldrin OR chlordan* OR dichlorvos OR mevinphos OR trichlorfon OR alachlor OR cyanazine OR dieldrin OR endrin OR hexachlorocyclohexane OR lindane OR endosulfan OR PFAS OR PFOS OR "perfluorooctane sulfonic acid" OR PFOA OR "perfluorooctanoic acid" OR phthalate* OR MEHP OR DEHP OR bisphenol* OR BPA OR arsenic OR cadmium OR mercury OR tributyltin OR organotin OR triphenyltin OR triclocarban OR triclosan OR paraben* OR carbamate OR carbaryl OR furan* OR neonicotinoid* OR imidacloprid OR acetamiprid OR dinotefuran OR thiamethoxam OR clothianidin OR fipronil OR PM2.5 OR PM10 OR particulate OR ozone OR "nitrogen oxide" OR "nitrogen dioxide" OR "sulphur dioxide" OR "sulfur dioxide"

		OR "carbon monoxide" OR "black carbon" OR "polyaromatic hydrocarbon" OR "PAH")
AND	Weight loss outcomes	TS= ("weight loss" OR "mass loss" OR "weight reduc*" OR "mass reduc*" OR weight near/1 reduc* OR "waist circumference" OR "waist-hip ratio" OR "waist to hip ratio" OR BMI OR "body mass index" OR "body fat" OR "adiposity" OR "fat reduc*" OR "fat loss")
NOT	Bariatric surgery	TS = ("bariatric surgery" OR "bariatric procedur*" OR "Roux-en-Y " OR "gastric band*" OR "sleeve gastrectomy" OR "gastric bypass" OR "gastroplasty" OR "jejunoileal bypass" OR "stomach stapl*")
		Scopus
	Dietary induced, intentional weight loss in people or animals	TITLE-ABS-KEY (“Weight Reduction Program*” OR {Reducing Diet} OR {Fat-Restricted Diet} OR {Carbohydrate-Restricted Diet} OR intentional OR {dietary intervention} OR {caloric restriction} OR {calorie restriction} OR {meal replacement} OR (diet w/1 restrict*))
AND	Chemical exposure	TITLE-ABS-KEY (“Chlorinated Hydrocarbon*” OR “Brominated Hydrocarbon*” OR “Fluorinated Hydrocarbon*” OR "Halogenated Hydrocarbon*" OR dioxin* OR {Dioxin-like} OR {Halogenated Diphenyl Ether} OR “Persistent Organic Pollutant*” OR Pesticide OR {Dibutyl Phthalate} OR {Diethylhexyl Phthalate} OR {Organotin Compound} OR Paraben OR “polychlorinated biphenyl*” OR “polyaromatic hydrocarbon*” OR Aroclor OR TCDD OR “polybrominated diphenyl ether*” OR “polybrominated diethyl ether*” OR PBDE* OR organochlorine* OR dichlorodiphenyltrichloroethane OR {dichlorodiphenyl trichloroethane} OR DDE OR dichlorodiphenyldichloroethylene OR {dichlorodiphenyl dichloroethylene} OR DDD OR heptachlor OR nonachlor OR HCB OR hexachlorobenzene OR pyrethroid OR "phenoxybenzoic acid" OR aldrin OR chlordan* OR dichlorvos OR mevinphos OR trichlorfon OR alachlor OR cyanazine OR dieldrin OR endrin OR hexachlorocyclohexane OR endosulfan OR PFAS OR PFOS OR {perfluorooctane sulfonic acid} OR PFOA OR {perfluorooctanoic acid} OR phthalate* OR MEHP OR DEHP OR bisphenol* OR BPA OR arsenic OR cadmium OR mercury OR tributyltin OR organotin or triphenyltin OR triclocarban OR triclosan OR paraben* OR carbamate OR carbaryl OR furan* OR neonicotinoid* OR imidacloprid OR acetamiprid OR dinotefuran OR thiamethoxam OR clothianidin OR fipronil OR PM2.5 OR PM10 OR particulate OR ozone OR {nitrogen oxide} OR {nitrogen dioxide} OR {sulphur dioxide} OR {sulfur dioxide} OR {carbon monoxide} OR {black carbon} OR {polyaromatic hydrocarbon} OR PAH)

AND	Weight loss outcomes	TITLE-ABS-KEY ({weight loss} OR {mass loss} OR “weight reduc*” OR “mass reduc*” OR (weight w/1 reduc*) OR {waist circumference} OR {waist-hip ratio} OR {waist to hip ratio} OR BMI OR {body mass index} OR {body fat} OR adiposity OR "fat reduc*" OR {fat loss})
AND NOT	Bariatric surgery	TITLE-ABS-KEY (bariatric surgery OR bariatric procedur* OR "Roux-en-Y " OR "gastric band*" OR "sleeve gastrectomy" OR "gastric bypass" OR "gastroplasty" OR "jejunoileal bypass" OR "stomach stapl*")

Table S3: Signal questions and the criteria for overall risk of bias rating for each of seven domains considered.

		Overall domain assessment		
Domain	Question	Low	Moderate	High
1: confounds	Were confounding factors identified?<br>Were strategies to deal with confounds stated?<br>What confounds, if any, did not appear to be accounted for?<br>Did the participants receive similar treatment, other than the dietary intervention?<br>What is the predicted direction of bias due to unaccounted for confounds?<br>Reason for your answers	Information on confounding variables for exposure status, mass change or diabetes status collected, reported and accounted for in analysis; participants experienced equivalent treatment other than the diet intervention; differences between participants added variability but were unlikely to produce systematic bias	Confounds partially accounted for and/ or differences between people in dietary support provided could have been confounded with exposure status and thus reduce apparent exposure effects	Confounds not identified, or present but not controlled for in analysis and could have substantially influenced the findings
2. Participant selection	Were inclusion criteria such as sex, age and demographic/ source population clearly stated?<br>What inclusion criteria were missing, if any?<br>Were specific medications excluded?<br>Were specific health conditions excluded?<br>Were specific lifestyle factors excluded?<br>Indicate the exclusions identified	No lack of clarity or some lack of clarity in randomisation or allocation concealment processes, but these	Insufficient information provided for recruitment or study design excluded or obscured some important demographics, such	Clear selection bias that may have strongly influenced the findings

	Are other relevant aspects of the participants' individual characteristics reported that may influence their weight loss trajectory during the diet?<br>List any characteristics that were provided or were missing<br>If overweight or obesity was included/ excluded, are the criteria used to define the condition provided?<br>Were participants recruited from a particular demographic or setting eg weight loss clinic, general population, high exposure population?<br>If yes, what setting or demographic did the study recruit from?<br>Are the demographics of the participants, such as age, sex, socioeconomic group, ethnic background and their relevant clinical characteristics reported?<br>Is the location (city and country) reported?<br>Is the year(s) of recruitment to the study reported or easily available (for example in a clinical trial registration)?<br>If participants were diabetic, is the duration of diabetes reported?<br>Were participants weight stable and in steady state at start of the diet intervention?<br>If diabetes or IGT was excluded/ included, are the criteria for diabetes diagnosis provided?<br>Is complete and consecutive inclusion used?<br>For randomised controlled trials only: was true randomization used for assignment of participants to treatment groups?	did not substantially impact on findings	as those with higher body weight or those who had earlier failed to lose weight.	

	Where a 'control' group used, is the diet clearly specified and matched in some way with the weight reduction diet and or lifestyle changes requested in control group clearly specified?<br>What is the predicted direction of bias due to the information available about participants and their characteristics?<br>Please provide any additional information that shows the reason for your answers			
3. Diet intervention	Is the diet duration provided?<br>Is the nature of the diet described in sufficient detail, such as total calories and composition by macronutrient group/ specific food stuffs and timing of meals?<br>What was the setting in which meals were eaten?<br>How was compliance/ adherence to diet ensured?<br>Is there a possibility that participants did not adhere to the diet?<br>Is the diet duration adequate to allow for sufficient weight loss to occur or glycaemic control be established?<br>Were participants blind to treatment assignment?<br>Were parameters for 'completion' of the diet programme clearly reported?<br>What is the predicted direction of bias due to the identified potential for departures from the intended diet?<br>Reason for your answers	Diet duration long enough and caloric restriction severe enough to observe clinically important differences; efforts made to adjust initial diet proportional to metabolic rate and/ or activity levels in a consistent way; sufficient information on diet composition; assessment of diet adherence performed; inclusion of diet adherence in analysis.	Lack of clarity on diet or where there were possibilities for participants to modify diet without the knowledge of the study co-ordinators, or where diet adherence was not fully accounted for in the analysis	Strong possibility that participants had the opportunity to deviate from the diet and for that to go unrecorded or unaccounted for and/ or when compliance assessment and parameters for completion of the diet programme were undescribed and unaccounted for.
4. conta minan	How is the exposure measured in the individual?<br>Is the exposure measured in an appropriate and validated matrix?	As recommended (Morgan et al 2018b), evaluate the cross-	Issues of temporality when measurement did not precede the	Methods were not validated, or represented a

	Is the exposure measurement from a single spot sample or more than one measurement (eg 24 h urine)?<br>Is it appropriate for exposure to be estimated from the sampling regime use?<br>Is the exposure measured in way that is either the gold standard for the chemical or another well accepted way?<br>Is a single chemical measured or are multiple congeners/ metabolites within a given group measured?<br>Is there a concern that the variation in exposure levels was insufficient to identify associations with mass loss rate or glycaemic control?<br>Was information on exposure status recorded prior to the diet intervention?<br>Is it clear exactly when baseline and endpoint exposure data was collected relative to start and end of the dietary intervention?<br>Was the timing of baseline and endpoint exposure measures relative to the start and end of the diet consistent between participants?<br>Could measurement of exposure status have been affected by knowledge of the outcome (mass change)?<br>What is the predicted direction of bias due to exposure measurement?<br>reason for your answers	sectional nature of the studies in terms of exposure status measurement as low risk of bias if measurement was performed correctly; measurements obtained were unlikely to be biased because efforts were made to perform procedural blanks; range represents a large enough difference between low and high exposures to see true effects where they exist.	diet or if change in exposure levels during the diet rather than 'baseline' was used in analysis; lack of clarity over the efforts made to minimise potential for either adsorption of chemicals of interest into the storage and sampling vessels or, conversely, for exposure to be overestimated by leaching of chemicals from plasticware to the sample matrix, but where such effects on measurement were likely to affect all measurements equally; standards used in exposure determination methods may not have been adequate for accuracy or where the range of values	population average rather than at individual participant level and taken over time spanning the intervention

			obtained may have been too narrow to see true differences where they exist	
5. missing data	Was follow up complete (ie was there missing data)?<br>Were the reasons for loss to follow up described and explored?<br>Was there adequate completion (ie over 80% of participants followed through to completion) to prevent bias?<br>Were strategies used to address incomplete follow up, i.e. considering time in the denominator.<br>Are the proportion of participants and reasons for missing data similar across exposures?<br>What is the predicted direction of bias due to missing data?<br>reason for your answers	Reported 80% or more completion rates; overall attrition rates low; reasons for drop out reported	Differences in frequency of recording between participants that could have contributed bias in data collection, including lack of reporting on those losing less weight for example. Account for apparent 100% completion rates in post hoc analyses where non completers are discarded in domain 7 where required.	Lack of reporting on completion and follow up or very low completion rates an no explanations provided
6. Outcome measurement	How was body mass and/ or the presence of obesity/ overweight assessed?<br>If diabetes or IGT or blood glucose was assessed (our secondary outcome), how was this done?<br>Were other secondary outcomes measured in a standard way?<br>Was allocation to diet concealed from the study co-ordinators/ those assigning the patients?	Measurements performed in a consistent way by a professional and in a medical setting and when the person undertaking the measurement was not	Measurements done in a community setting by trained participants	Self-reported with no evidence of control or validation of the values reported; lack of concealment to intervention could have influenced outcome reporting

	Were outcomes assessors blind to treatment assignment or exposure status?<br>Is participants' body mass and/ or BMI at recruitment reported?<br>Is it clear exactly when baseline and endpoint outcome data were collected relative to start and end of the dietary intervention?<br>Was the timing of baseline and endpoint outcome measures relative to the start and end of the diet consistent between participants?<br>What is the predicted direction of bias due to outcome measurement?<br>reason for your answers	aware of diet intervention and/ or exposure status		
7. reporting and statistical analyses	Was power analysis performed?<br>Is the reported effect (or lack thereof) likely due to inadequate power/ sample size?<br>Is the methods section detailed enough to identify which statistical techniques were used?<br>Are the statistical techniques used to assess impact of chemical on mass loss or glycaemic control appropriate?<br>Was repeated measures analysis used where appropriate?<br>Was the study protocol available and were all of the study's pre-specified primary and secondary outcomes reported in the current manuscript?<br>If the study protocol was not available, was it clear that the published report included all expected outcomes (i.e. comparing methods and results section)?	Sufficient information in methods to identify techniques used and those techniques were appropriate and internally consistent with results; power analysis performed or sample size large enough for true effects to be detected where they exist; full follow up results reported or reasons given for lack of longer follow up reporting; protocols	Statistical methods not fully explained; unplanned analyses performed; not possible to match results to methods or a protocol; lack of power analysis and small sample size could have failed to find an effect where one exists; incomplete follow up reporting may have occurred; where selective outcome or analysis reporting	Apparent selective reporting based on internal inconsistences within the paper or with other papers on the same study.

		and/or adherence to STROBE guidelines (von Elm et al 2008) was reported.	could have occurred; differences in follow up timeframe and completion rates could have influenced the findings given non linearity of weight loss trajectory.	
	Is the reported effect estimate likely to be selected from multiple outcome measurements?<br>Is the reported effect estimate likely to be selected from multiple analyses of the exposure-outcome relationship?<br>Is the reported effect estimate likely to be selected from different sub groups?<br>What is the predicted direction of bias due to statistics and reporting?<br>reason for your answers			

Answers to closed questions could be 'yes', 'no', 'partly', 'unreported', NA or 'unclear/ unsure' except when a direction of bias was required, when 'leads to over estimate' or leads to underestimate', 'NA' or 'unclear' could be selected. Other questions allowed free text.

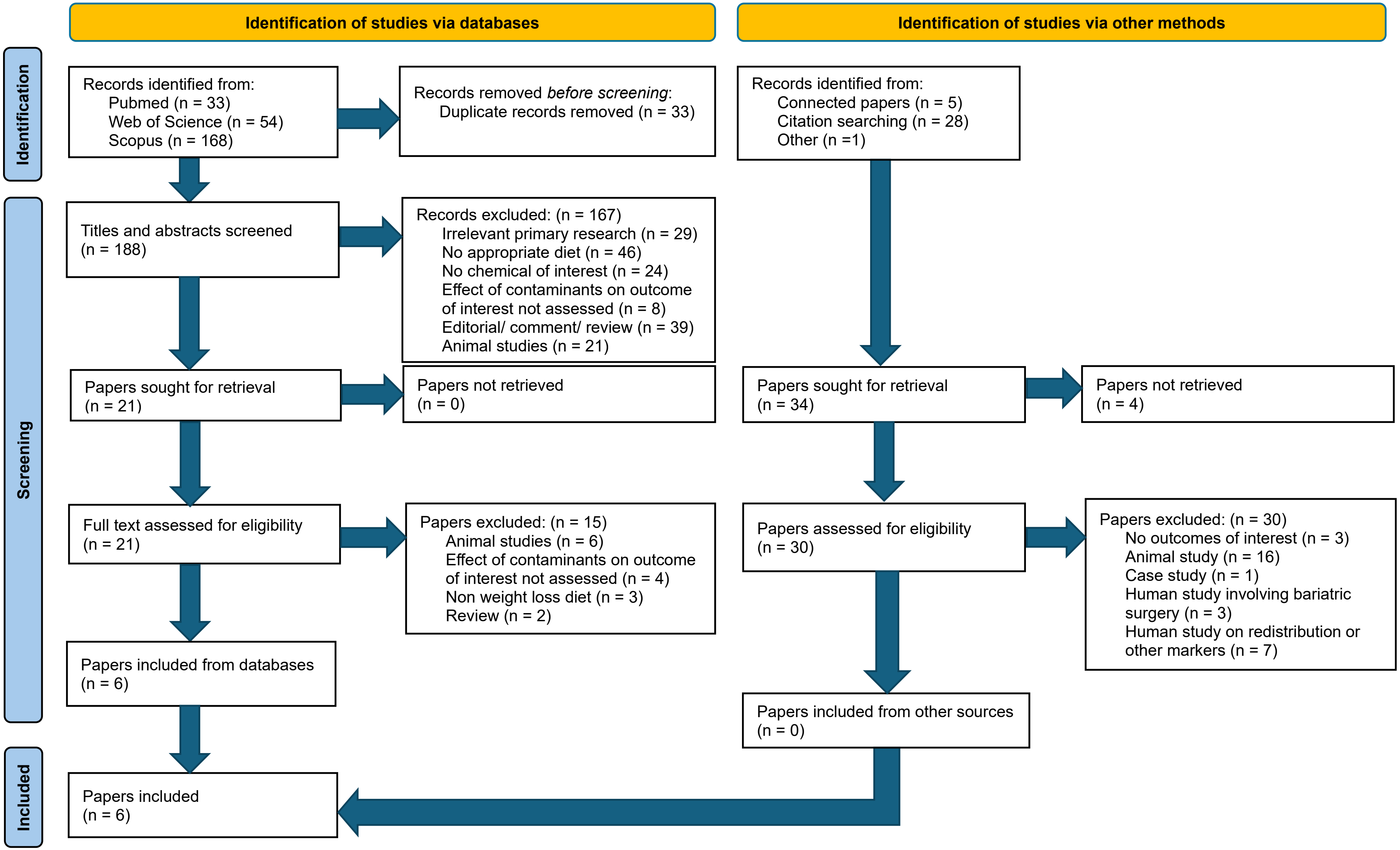
Identification of studies via databases
Identification of studies via other methods
Identification
Screening
Included
Records identified from:
Pubmed (n = 33)
Web of Science (n = 54)
Scopus (n = 168)
Records removed *before screening*:
Duplicate records removed (n = 33)
Records identified from:
Connected papers (n = 5)
Citation searching (n = 28)
Other (n =1)
Titles and abstracts screened
(n = 188)
Records excluded: (n = 167)
Irrelevant primary research (n = 29)
No appropriate diet (n = 46)
No chemical of interest (n = 24)
Effect of contaminants on outcome of interest not assessed (n = 8)
Editorial/ comment/ review (n = 39)
Animal studies (n = 21)
Papers sought for retrieval
(n = 21)
Papers not retrieved
(n = 0)
Papers sought for retrieval
(n = 34)
Papers not retrieved
(n = 4)
Full text assessed for eligibility
(n = 21)
Papers excluded: (n = 15)
Animal studies (n = 6)
Effect of contaminants on outcome of interest not assessed (n = 4)
Non weight loss diet (n = 3)
Review (n = 2)
Papers assessed for eligibility
(n = 30)
Papers excluded: (n = 30)
No outcomes of interest (n = 3)
Animal study (n = 16)
Case study (n = 1)
Human study involving bariatric surgery (n = 3)
Human study on redistribution or other markers (n = 7)
Papers included from databases
(n = 6)
Papers included from other sources
(n = 0)
Papers included
(n = 6)

Fig S4: PRISMA 2020 flow diagram showing outcome of searches of databases and other sources, screening and final inclusion of papers and studies. Based on layout from Page et al (2021).

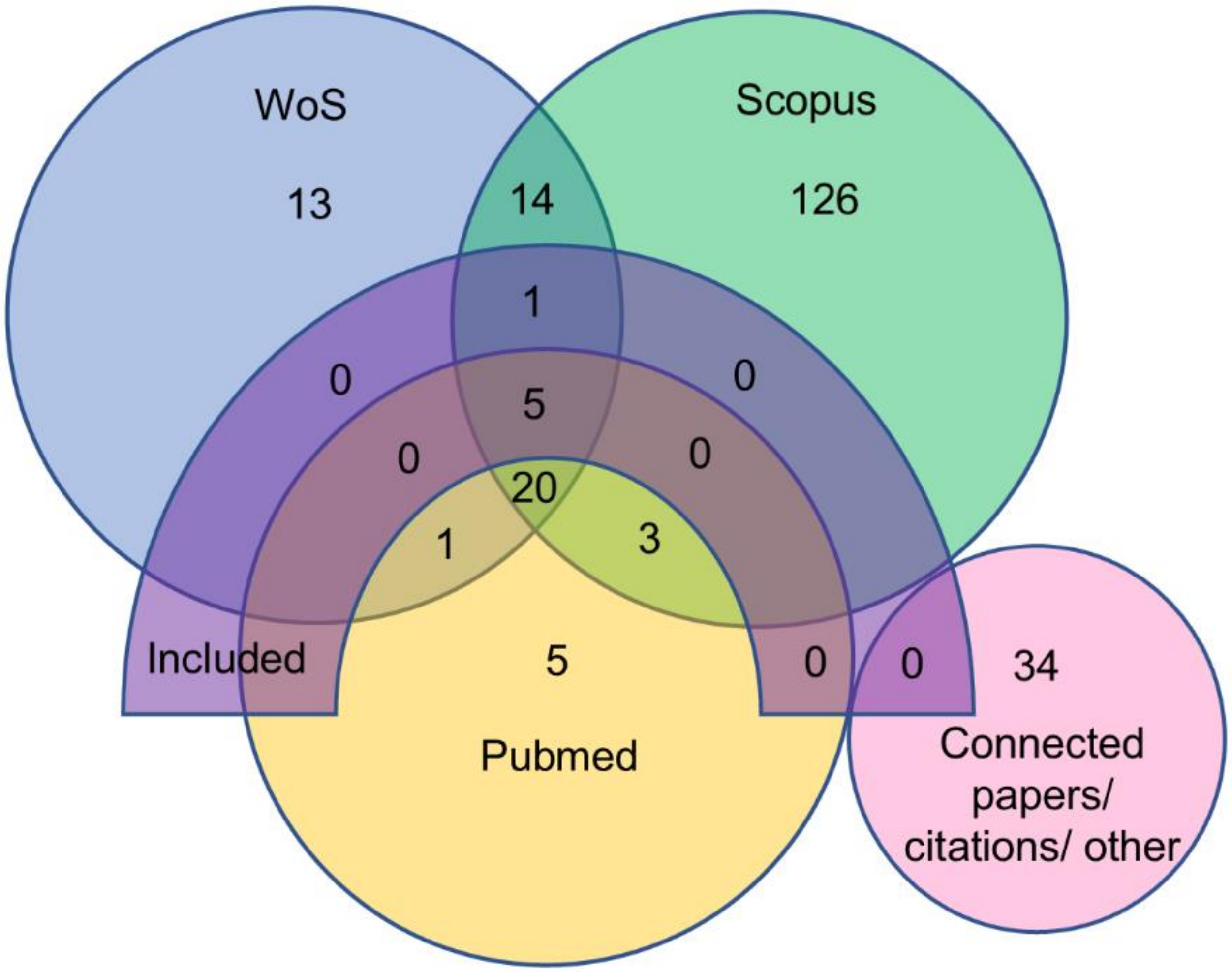

Figure S5: Origin of the papers assessed in this study. Included papers are covered by the arc transecting the three overlapping ellipses and the independent circle that includes references list and Connected papers searches, which yielded papers that were not identified in the data base searches. WoS = web of science.

Table S6: Study characteristics details of the six identified studies that assessed impact of contaminants on mass loss or glycaemic control parameters

		Liu et al (2018)	Ustulin et al (2018)	Van der Meer et al (2021)	Grandjean et al (2023)	Kahleova et al (2016)	Imbeault et al (2002)
Study design		Post hoc analysis on large randomised controlled trial designed to examine the efficacy of different macronutrient diets on weight loss (Sacks et al 2009; NCT00072995).	Two large retrospective cohort studies	Post hoc analysis on large randomised controlled trial designed to examine the efficacy of different macronutrient diets on weight loss (Soenen et al 2012: NCT00862953)	Post hoc analysis on large randomised controlled trial designed to examine the efficacy of different macronutrient diets on weight regain after a low calorie weight loss period (Moore et al 2009; Larsen et al 2009: NCT00390637)	Post hoc analysis on smaller randomised controlled trial designed to examine the effect of a vegetarian diet on oxidative stress and insulin resistance in T2DM patients (Kahleova et al 2011: NCT00883038).	Small prospective cohort study intended to explore effects of POP release from adipose during weight loss on insulin levels and glucose tolerance.
Participant characteristics	Recruitment method	Recruited through mass mailing of the general population (Sacks et al 2009). Participants were overweight or obese, based on standard BMI cut off values.	People who had downloaded the Noom Coach app to their smartphone, irrespective of initial weight or BMI	Patients with BMI of over 27 were referred from a weight loss clinic (Soenen et al 2012).	Families with one adult of BMI >27 and <45 on waiting lists for weight loss projects, or referred from GP or other medical establishment; fliers, posters, advertisements on radio, TV, newspapers and internet to target general population (Larsen et al 2009).	Referred by endocrinologists (Kahleova et al 2011). Participants were overweight or obese, based on standard BMI cut off values.	Recruited through general adverts. Participants were overweight or obese, based on standard BMI cut off values.

	Ethnicity	Predominantly white (78%), with a small proportion of Black participants (16%) and only single digit numbers of people that identified as Hispanic, Asian or Other ethnic groups (given in Sacks et al 2009).	Ethnicity data were not reported	Ethnicity data were not reported here or in Soenen et al (2012).	Ethnicity data were not reported here or in Larsen et al (2009) or Moore et al (2009)	Ethnicity data not reported here or by Kahleova et al (2011).	All participants were 'Caucasian'
	Sex (% female)	62	Study 1: 75<br>Study 2: 75	68	70	53	54
	Sample size	621	Study 1: 2608<br>Study 2: 995 (subset of study 1)	218	381	74	37
	Age range	30-70	Study 1: 37.9-38.8 (95%CI)<br>Study 2: unreported	23-71	24-63	30-70	36-50 (Imbeault et al 2001)
	Location	USA	Study 1: Cities across USA, South Korea, Japan, Netherlands, Germany, UK and Australia<br>Study 2: USA cities	Netherlands	8 countries across Europe (Netherlands, Denmark, UK, Greece, Germany, Spain, Bulgaria, Czech Republic)	Czech Republic	Canada

	T2DM status	DM was an exclusion criterion. T1DM or T2DM is not specified, but Sacks et al (2009) showed no evidence of high fasting blood glucose or insulin at baseline on, suggesting there were not many people with pre-diabetes despite high BMI.	Unreported	19% of participants had DM. T1DM was excluded, suggesting that participants with DM therefore had T2DM.<br><br>DM status was recorded using a questionnaire.	T1DM and T2DM was an exclusion criterion listed in Goyenechea et al (2011)	Patients with T2DM who were taking hypoglycaemic agents and not insulin. HbA1c between 6 and 11%.	DM excluded

	Exclusion criteria	Unstable cardiovascular disease, use of medications that affect body weight, and insufficient motivation to maintain the diet	Used the app for less than 12 months; did not provided starting weight and height; did not provide weight and meal information at least monthly; 42 years old; living outside cities for which PM10 and PM2.5 data were available for 2013 and 2014	Cancer, HIV, large recent weight loss and psychiatric illness, pregnancy and breastfeeding.	BMI > 45; adult offspring still living at home; adults who did not have parental responsibility for a child under 18; Subjects using prescription medication, or suffering from diseases that can influence body weight regulation (malabsorption, untreated thyroid disease, eating disorders, systemic use of steroids, food allergies, cancer within 10 years.) and obesity-related cardiovascular risk factors (heart disease, systolic and diastolic blood pressures 160/100 mmHg, blood glucose > 6.1 mmol L-1, blood cholesterol > 7 mmol L-1, blood triglycerides > 3 mmol L-1; Marked alcohol habits; planned major	HbA1c < 6% or > 11%, use of insulin, abuse of alcohol or drugs, pregnancy, lactation, or current use of a vegetarian diet.	Cardiovascular disease, other endocrine disorders at initial medical check-up, or those on medication that could have influenced triglyceride metabolism, such as β-blockers or antihypertensive drugs.<br><br>Recently been on a diet or involved in a weight-reducing program

					changes in physical activity during the study to an extent that might interfere with the study outcome; blood donation within the previous 2 months; weight change of >3 kg within 2 months prior to first clinical investigation day; psychiatric disease; pregnant or lactating women, or those planning to become pregnant within the next 18 months; surgically treated obesity; participation in other clinical studies within the previous 3 months; drug abuse; unable to give informed consent or engage 8-week low-calorie diet; those already on a special diet (vegetarian, Atkins etc)		

	Lifestyle characteristics	4% current smoker, 37% former smokers; 68% college graduates or beyond; 70% married; 84% income of up to $150,000; exercise habits not reported (Sacks et al 2009)	Number of steps recorded daily	Not heavy smokers or drinkers; patients stratified into diet groups by physical activity assessed by Baecke's score of physical activity (Soenen et al 2012)	Not heavy drinkers; exercise habits recorded but not used in exclusion/ inclusion (Laren et al 2009). Socioeconomic information not reported here or elsewhere (Larsen et al 2009; Moore et al 2009; Goyenechea et al 2011).	19% were smokers; exercise habits not reported here or in Kahleova et al (2011)	All participants typically partook in fewer than 2 exercise sessions of 30 minutes/week, were non-smokers, and were moderate alcohol consumers (fewer than 140 g/week). Body weight had been stable during the 6 months prior to the start of the study

	Diet Intervention	6-month weight loss intervention on one of four randomly assigned diets (low fat, average protein; low fat, high protein; high fat, average protein and high fat, high protein and all included low glycaemic index food, with 8% or less saturated fat, 150mg cholesterol or less per 1000 kcal and a minimum of 20g dietary fibre per day (Sacks et al (2009)) of 750 kcal deficit from baseline, which represents a 30-37% reduction in calorific intake relative to average requirements, but the exact proportion depends on the		3-months on one of 4 different diets (normal protein and normal carbohydrate; normal protein, low carbohydrate; high protein- normal carbohydrate and high protein low carbohydrate) that represented a restriction to 33% of age and sex-based energy requirements.	8 weeks on 800-880 kcal/ day provided by 4 sachets of Modifast® per day, consisting of 42% fat, 44% carbohydrate and 14% fat, and supplemented by drinks, creams and soups and up to 200 g/day tomatoes, 125 g/day cucumber 50 g/day and lettuce (Goyenechea et al 2011).	Vegetarian (60% carbohydrate 15% protein, 20% fat) and non-vegetarian (50% carbohydrate; <30% fat; 20% protein; <7% saturated fat and <200mg/ day cholesterol) antidiabetic diets of 500 kcal deficit for 12 weeks.	Non-macronutrient specific diet of 700kcal less than daily energy expenditure for 15 weeks.<br><br>Some of the participants also received fenfluoramine, an appetite suppressant

		participants' original intake.					
	Energy intake calculation	Calculated per person based on measurements of resting metabolic rate using indirect calorimetry and their physical activity (Sacks et al 2009).	Noom Coach creates individualised plans based on intended weight loss and time, sex and typical activity patterns	Energy intake calculations performed for each person, based on the Harris Benedict equation for estimation of basal metabolic rate (Harris and Benedict, 1918), with a multiplier to adjust for physical activity and were assumed to be isocaloric across participants (Soenen et al 2012).	Calculated on food content, not on energetic requirements of individual participants.	Calculated per person based on measurements of resting metabolic rate calculated using indirect calorimetry (Kahleova et al 2011)	Calculated per person based on measurements of resting metabolic rate using indirect calorimetry and an activity factor of 1.4.
	Follow -up	6 to 12 months after the diet had ended to examine impacts of earlier chemical exposure on weight regain.	Those who used the app for at least 12 months	6 month follow up period when the participants were moved to a weight maintenance phase at 67% baseline energy requirements (Soenen et al 2012), but follow up was not reported here	Reports on 6 months follow up but other studies report longer follow up of 12 months (Larsen et al 2009). Only 101 people remained in the study for 12 months.	24 week follow up (Kahleova et al 2011) not reported in Kahleova et al (2016). The second 12 weeks included prescribed exercise	None reported in this paper, but have been included in larger study (eg Doucet et al 1999; Chevrier et al 2000).

Chemical	Matrix	Plasma	Average annual (study 1) or daily levels in air (study 2) for that city and converted to an air pollution index	Urine	Plasma	Serum	Plasma
	Lipid correction	NA	NA	NA	NA	no	yes
Outcomes reported	Weight or body fat change	Mass (kg), BMI, waist circumference (cm), % fat,	BMI change	BMI, waist circumference (cm), % fat	Mass (kg). BMI, waist, hip and sagittal circumference (cm) and % fat measured but not reported here (Larsen et al (2009)	Mass (kg), BMI, % fat	Mass (kg), BMI, % fat
	Glycaemia control metrics and units	Baseline fasting glucose (mM) and fasting insulin (pM). Change in above and HOMA-IR, HOMA-β; Hb1Ac (%) also all available in Sacks et al (2009)	None	None in van der Meer et al (2021). Fasting glucose (mM); fasting insulin (µU/ml) and HOMA index presented in Soenen et al (2012)	None reported here. OGTT, fasting blood glucose (mM) and insulin (µU/ml) reported in Larsen et al (2009) and Goyenechea et al (2011)	Clearance rate of glucose in last 20 min of hyperinsulinaemic isoglycaemic clamp; Insulin secretion in standardised meal test (insulin units not presented); Hb1Ac (%); fasting glucose (mM)	Fasting glucose (units not presented; data not shown); fasting insulin (pM), AUC in GTT

	Reported changes in mass / fat content metrics as a result of diet	Participants lost an average of 6.4 kg over the first 6 months of the trial, representing about 7% of their initial weight, and then regained an average of 2.7kg over the following 6-24 months (Sacks et al 2009).<br><br>Across all diet groups, 31 to 37% of the participants had lost at least 5% of their initial body weight by the 2-year follow up and 14 to 15% had lost at least 10% of their initial weight (Sacks et al 2012). Only 2 to 4% had lost 20 kg or more. Details of body composition and other metrics were not presented.	Participants showed an average decrease in BMI of 2.139 kg/m2 (95% CI, –2.255 to –2.024).<br><br>~ 80% lost weight, 18% gained weight and ~2% maintained weight<br><br>People from Seoul had lowest initial BMI, highest pollution exposure and saw least reduction in BMI	Participants lost an average of 10 to 14 kg depending on the diet they were on, but showed no difference between diet types (Soenen et al 2012), which allowed grouping of all diet interventions.<br><br>Fat mass fell by 8.6 to 12.1kg, representing a 4-6.2% fall in body fat content across all groups, and waist to hip ratio fell from 0.95 to 0.93 in all but one (normal protein, low carbohydrate) diet groups, suggesting clinically relevant weight loss occurred in these participants.	Participants lost 5.6-28.3kg. Only those who lost 8% or more of their original body mass in the low calorie phase were included in the subsequent randomisation of weight maintenance ad libitum diets presented here.	Mass loss of ~ 6kg in vegetarian and ~ 3.5kg in control diet, 2.15 point reduction in BMI in vegetarian and 1.25 point reduction in standard diet groups.<br><br>Both diets produced 5-6cm reduction in waist circumference and ~6% reduction in sc fat volume.<br><br>The vegetarian diet decreased visceral fat volume by ~9% and the control diet by 3% (Kahleova et al 2011).	Body mass fell by 11kg (10.5%) in men and 9kg (9.9%) in women, on average.<br><br>Men experienced a reduction in BMI of 4 points and women of 3. Body fat content fell by 8% in mean and 2% in women, equating to 10kg in men and 6kg in women.

	Reported changes in glycaemia control metrics	Fall in fasting glucose of 1.2-3%, fall in fasting insulin of 14-19.9%; 13-4- 22.7 % reduction in HOMA across all diets but statistical significance unreported (Sacks et al 2009)	NA	No change in fasting glucose across all diets; significant reduction in fasting insulin of 33-45% across diets and significant fall in HOMA index in all cases (Soenen et al 2012.	Not reported here. Reduction in fasting glucose (5.11mM to 4.83 mM; n = 755) and insulin (11.7-8.13 µU/ml; n = 672) and AUC in OGTT (20.1 to 19.4mM/180min; n = 718) in the larger cohort of people from which this subset was drawn (Goyenechea et al 2011)	Both diets produced an average 1.45mM drop in fasting glucose. Fasting plasma insulin was reduced by 1.92 and 2.85nM in control and vegetarian diets respectively, and. Hb1Ac fell by 0.59% in control and 0.68 % in the vegetarian diet (Kahleova et al 2011).	Fasting insulin fell from 123pM to 93pM in men (30pM reduction) and more modestly from 96 to 86 pM (10pM reduction) in women.<br><br>AUC in the 3 h OGTT fell in both sexes between baseline and 15 weeks.
Analysis	Covariates (see table S6 for more detail)	Age, sex and ethnicity, leptin levels, thyroid hormone levels, smoking status, educational attainment, alcohol consumption, menopausal status, hormone replacement therapy use, and habitual exercise as well as the diet groups and baseline BMI or RMR	Study 1: Age, sex, location, daily caloric intake, input frequency of physical activity<br><br>Study 2: age, sex, season and time of log in, estimated daily calorie intake, daily precipitation, daily temperature	Initial adiposity trait, age and dietary intervention	Assuming same analysis applied to LCD period as applied to weight maintenance: age, sex, weight loss tertile, diet group, baseline BMI, family type (single or both parents) and location that food was obtained in weight maintenance phase	Analysis with and without adjustments for fat volume and BMI were performed	No differences in the weight change trajectory and other metabolic characteristics of the fenfluoramine group compared to control and thus pooled the data from both in subsequent analysis.<br><br>Sex included as a factor in analysis.

	Method	Participants were divided into tertiles of baseline PFAS exposure.<br><br>Linear regression was used to explore the association between baseline PFASs and change in body weight and RMR during both the 6 months of weight loss and then the period of weight regain.	Paired t test of BMI at start and end (last log in day)<br><br>Study 1: Multi level model exploring effect of PM2.5 and PM10 on BMI change with user nested in city and accounting for age, sex, location, daily caloric intake, input frequency of physical activity<br><br>Study 2: mixed effects model to examine effect of pollution index on BMI change accounting for age, gender, season of login, time of login, estimated average daily calories intake, daily intensity of precipitation, and daily temperature. A time by pollutant interaction effect was included	Linear multivariate analysis of the effect of the chemicals on each of the adiposity related traits.<br><br>Both raw p values and adjustments of false discovery rates were reported.	Linear mixed model for each PFAS individually and then including all PFAS using quantile g-computation to account for mixtures. Time component added for data points during weight maintenance phase but not during LCD phase.	Body weight, BMI, volume of visceral fat, insulin sensitivity, HbA1c, parameters of β-cell function were analysed separately using an orthogonal projection to latent structure (OPLS) model to account for collinearity among explanatory variables.<br><br>Changes in POPs were used as an independent variable. The relationship between each individual POP and the metabolic parameters was assessed using multiple regression.	A MANOVA was used to examine sex by time effects, followed by further analysis of a simple main effect for sex, and post hoc analysis using a paired *t* test.<br><br>Univariate associations between variables were examined using Pearson's product moment correlation coefficients and partial correlations were performed.

	Additional analyses	The association between baseline PFASs with baseline values of and changes in fasting glucose, insulin, Hb1Ac and HOMA-IR as well as lipids, thyroid hormones, and leptin were examined using Spearman correlation coefficients with adjustment for the potential confounders detailed above.	None	Exploration of the change in each chemical group over the 3 months of diet intervention; the effect of fat loss in urinary chemical excretion	Sensitivity analysis to establish effect of within couple correlations for both parent families. Allowance for missing values for people who dropped out at earlier time points. All these analyses relate only to post-LCD diet.	None reported	None reported

Findings	Associations with chemical exposure	No association between weight loss rate or resting metabolic rate during weight loss and PFASs.<br><br>After multivariate adjustment, including baseline levels of each metabolic parameter, plasma concentrations of perfluoro-octanesulfonic acid (PFOS), perfluoro-nonanoic acid (PFNA), and perfluoro-decanoic acid (PFDA) at baseline were negatively associated with changes in visceral fat mass during weight loss.<br><br>Baseline PFOA was negatively associated with weight-loss induced changes in	Study 1: People exposed to both PM10 and PM2.5 showed a smaller decrease in BMI than those exposed to lower levels of these pollutants across cities. Removal of cities with people with lowest BMI and highest pollutants (Seoul and Tokyo) removed the significant association of BMI reduction with PM10.<br><br>Study 2: the fall in BMI was less pronounced per unit time when pollutant levels were higher.	Positive association between higher baseline urinary paraben and phthalate levels and post-diet BMI, percentage body fat and waist circumference: higher baseline excretions of methyl (MeP) and propyl paraben (PrP) were associated with an impaired reduction in BMI. The reduction in waist circumference was less pronounced in those participants with higher propylparaben, mono-n-butyl (MBP) phthalate, and mono-benzyl phthalate (MBzP). Mono-ethyl phthalate (MEP) and MBzP were associated with impaired reduction in % body fat. After adjusting for	No association between PFAS and relative weight loss during LCD phase of the trial but analysis not shown.<br><br>Doubling of PFOA associated with increase of 1.5kg over 26 week maintenance diet, equating to 5kg weight gain in people on best performing diet over 52 week post -LCD weight maintenance study. Increase of 1kg with doubling of baseline values associated with other PFAS when considered individually.<br><br>Weight gain of 1.66kg associated with doubling of all PFAS. PFHxS, PFOA, PFDA all produce positive co-efficients and PFOS and PFNA produce negative co-efficients	An effect of the increase in POPs during 12 weeks of weight loss on glycaemic control in T2DM patients, including a positive association between the increase in circulating POPs and both Hb1Ac and fasting glucose.<br><br>A reduction in measures of β cell function associated with the increase in circulating POPs, even when adjusted for BMI and visceral fat content<br><br>No association between the increase in circulating POPs and insulin sensitivity.	Negative association between the change in OCs during weight loss and fasting insulin in men but not in women.<br><br>Neither sex exhibited changes in area under the curve of insulin during a GTT associated with the rise in plasma OCs during weight loss (Imbeault et al 2002).

		HDL cholesterol levels.<br><br>Baseline PFASs were positively associated with greater weight regain, particularly for women, which may be driven by a greater suppression of resting metabolic rate.<br><br>When the data were divided into tertiles, women in the highest PFAS concentration tertile regained 1.7 ± 2.2 kg more mass than those in the lowest tertile. PFOS and PFNA were the two main PFASs significantly associated with greater decline in RMR during the first 6 months of the diet, and a smaller increase in		multiple testing, the associations between MeP and PrP, and BMI, and MBzP and waist circumference and BF% remained significant.<br><br>When T2DM participants were removed from the analysis, the association between MEP and slower fat loss was no longer apparent. T2DM status did not affect the other findings.<br><br>There were no associations between BPA and reported mass loss characteristics.	when considered in a multi pollutant model.		

		RMR during the following 6–24-month weight regain period.<br><br>During the weight regain period, baseline PFOS, PFNA, and PFDA levels were positively associated with changes in waist circumference, and leptin, although these associations were weak.<br><br>Baseline PFOA and perfluoro-hexaneulfonic acid (PFHxS) were associated with a greater increase in visceral fat mass with r_s = 0.30 and 0.27, respectively.<br><br>Positive associations between PFOS, PFOA, and PFNA concentration at					

		baseline with insulin, HOMA-IR, diastolic blood pressure, and free T3 after multivariate adjustment.<br><br>PFHxS and PFDA were positively associated with some of the baseline variables, including visceral fat mass, systolic blood pressure, glucose, triglycerides, LDL cholesterol, free T4, total T4, and leptin.<br><br>Although these associations were statistically significant, the strength of association was weak, with r_s ranging from 0.08 to 0.24.					

		These associations were seen despite the lack of relationship between the PFASs and baseline body weight, waist circumference, BMI or RMR. However, no association between baseline PFASs and glycaemic control metrics following weight loss were reported.<br><br>During the weight regain period, baseline PFOS, PFNA, and PFDA levels were positively associated with changes in insulin although these associations were weak.					

References:

Chevrier J, Dewailly E, Ayotte P, Mauriège P, Després J P, Tremblay A. Body weight loss increases plasma and adipose tissue concentrations of potentially toxic pollutants in obese individuals. *Int J Obesity Related Metab Dis.* 2000. 24: 1272–1278. https://doi.org/10.1038/sj.ijo.0801380

Doucet E, St-Pierre S, Alméras N, Després JP, Bouchard C, Tremblay A. Evidence for the existence of adaptive thermogenesis during weight loss. *Brit J Nutr* 2001. 85: 715–723. https://doi.org/10.1079/bjn2001348

Goyenechea E, Holst C, van Baak MA. et al. Effects of different protein content and glycaemic index of *ad libitum* diets on diabetes risk factors in overweight adults: the DIOGenes multicentre, randomized, dietary intervention trial. *Diabetes Metab Res Rev* 2011.27:705–716. https://doi.org/10.1002/dmrr.1218

Imbeault P, Chevrier J, Dewailly E, et al. Increase in plasma pollutant levels in response to weight loss in humans is related to *in vitro* subcutaneous adipocyte basal lipolysis. *Int J Obesity Related Metabolic Dis* 2001. 25: 1585–1591. https://doi.org/10.1038/sj.ijo.0801817.

Imbeault P, Tremblay A, Simoneau JA, Joanisse DR. Weight loss-induced rise in plasma pollutant is associated with reduced skeletal muscle oxidative capacity. *Am J Physiol. Endocrinol Metab*. 2002. 282: E574–E579. https://doi.org/10.1152/ajpendo.00394.2001

Kahleova H, Matoulek M, Malinska H et al. Vegetarian diet improves insulin resistance and oxidative stress markers more than conventional diet in subjects with Type 2 diabetes. *Diabet Med.* 2011. 28: 549–559. https://doi.org/10.1111/j.1464-5491.2010.03209.x.

Larsen TM, Dalskov S, van Baak M et al. The Diet, Obesity and Genes (Diogenes) Dietary Study in eight European countries - a comprehensive design for long-term intervention. *Obesity Rev* 2010 11: 76–91. https://doi.org/10.1111/j.1467-789X.2009.00603.x

Moore CS, Lindroos AK, Kreutzer M et al. Dietary strategy to manipulate *ad libitum* macronutrient intake, and glycaemic index, across eight European countries in the Diogenes Study. *Obesity Rev* 2010. 11: 67–75. https://doi.org/10.1111/j.1467-789X.2009.00602.x

Sacks FM, Bray GA, Carey VJ et al. Comparison of weight-loss diets with different compositions of fat, protein, and carbohydrates. *NEJM*. 2009. 360: 859–873. https://doi.org/10.1056/NEJMoa0804748

Soenen S, Bonomi AG, Lemmens SG et al. Relatively high-protein or 'low-carb' energy-restricted diets for body weight loss and body weight maintenance? *Physiol Behav.* 2012. 107: 374–380. https://doi.org/10.1016/j.physbeh.2012.08.004

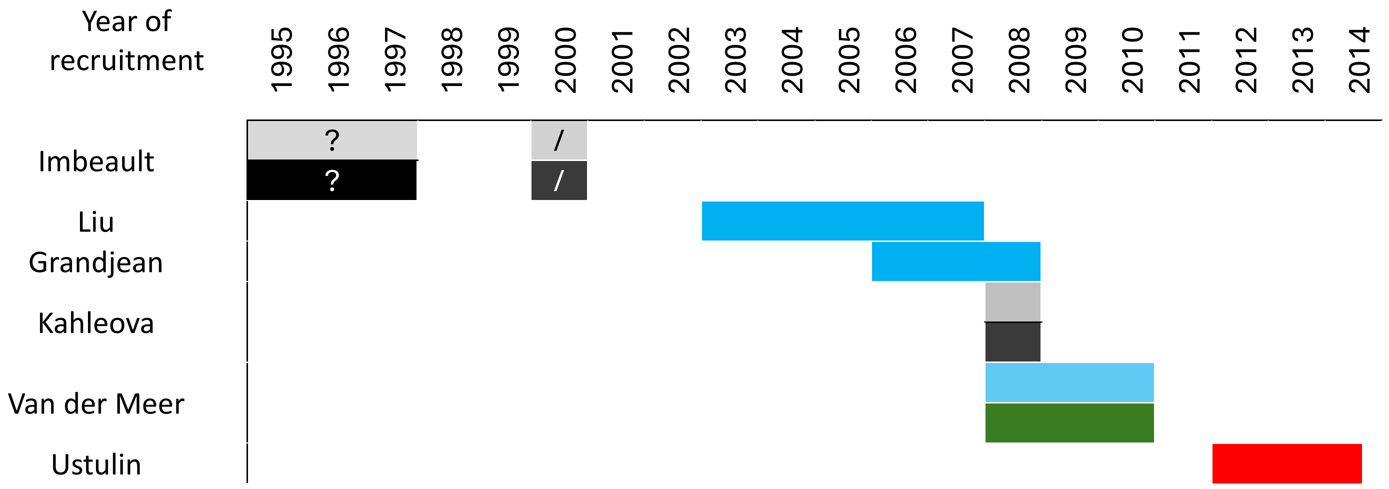

Figure S7: timeline of studies investigating contaminant exposure and weight loss in humans. Grey = OCPs; black = PCBs; mid blue = PFAS; yellow = parabens; dark green = plasticisers (BPA and phthalates); red = air pollutants. ? indicates where the study timeline is unreported but has been inferred from other papers that report on similar or the same participants. / indicates where study timeline is unknown and the year before publication has been highlighted as the latest time that the study could have been undertaken.

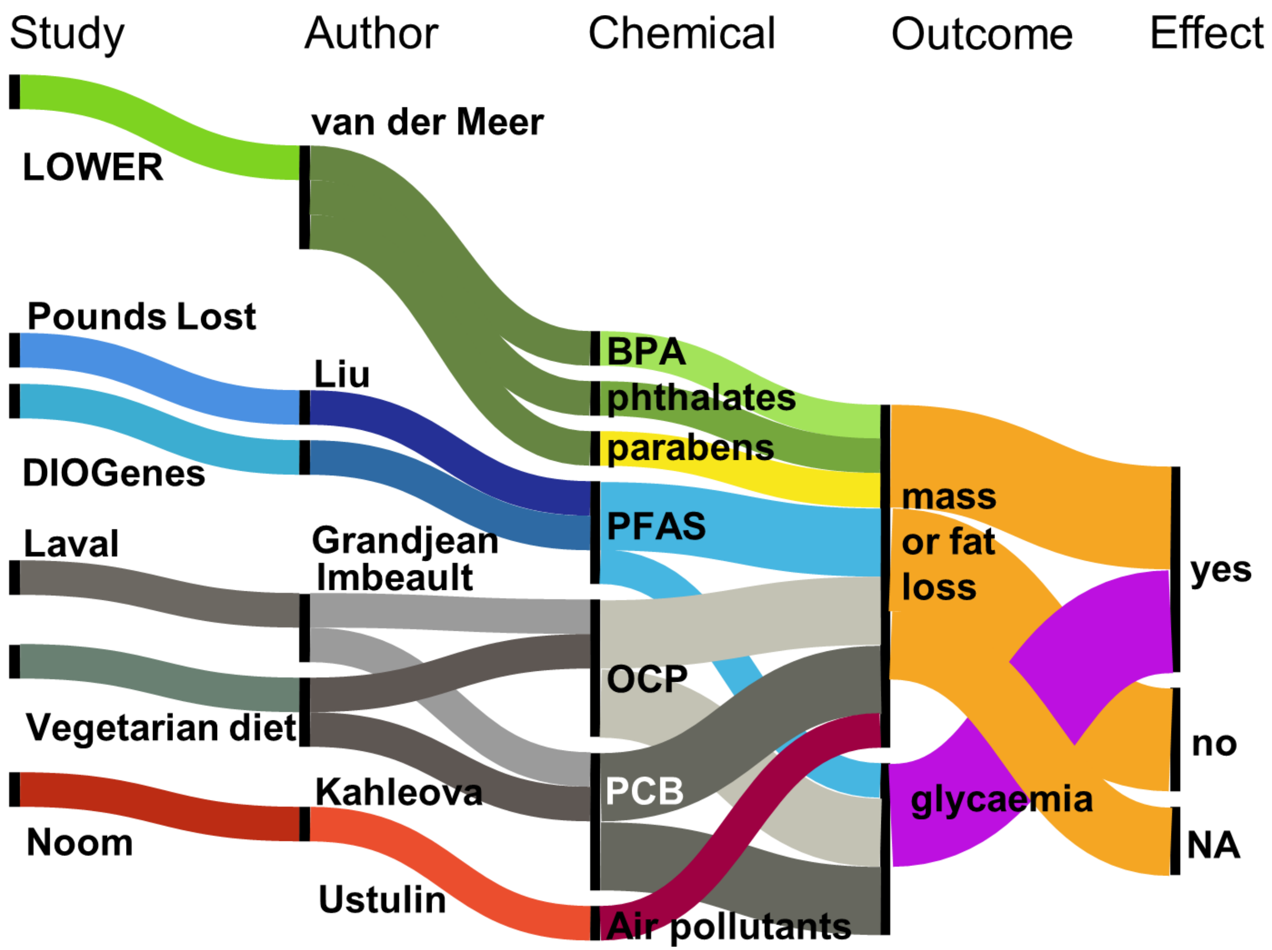

Figure S8. We used RAWgraphs (https://www.rawgraphs.io/; Mauri et al. 2017) to create a Sankey chart demonstrating information flow to summarise the current evidence base. Information flow from the six human weight loss diet intervention studies identified that investigated the impact of contaminant exposure on either mass loss or fat content metrics, or glycaemic control metrics or both. The wider studies in node 1 are linked to the individual papers in node 2, which link to the various chemical groups of interest they reported in node 3. The links from nodes 1-3 are broadly colour coded by chemical group of interest, with shades denoting different studies. Node 3 links to the outcomes of interest that were measured in node 4, which can be mass or fat loss (orange), or glucose regulation metrics (purple), or both. In turn, the outcome nodes are linked to the result in node 5, which indicates whether a significant

effect was reported or not, or whether, despite the availability of the data, that aspect was not tested (NA). Nodes are represented by black bars and links between them indicated by coloured lines, the thickness of which represents the relative size of the link, based on the number of instances of the connection between the two nodes (i.e. not number of papers or studies sine more than one chemical or outcome may be measured in any one paper). The figure highlights that more work has been conducted on persistent pollutants, including OCPs, PCBS and PFAS, than other pollutant types, a greater focus on mass change than glycaemic control metrics and the lack of current consensus on chemical effects across pollutant categories.

Mauri M, Elli T, Caviglia G, Uboldi G, Azzi M. RAWGraphs: A visualisation platform to create open outputs. In Proceedings of the 12th Biannual Conference on Italian SIGCHI 2017. Chapter (p. 28:1–28:5). New York, NY, USA: ACM. https://doi.org/10.1145/3125571.3125585.

Table S9: Detailed reported findings from the six studies that examined impact of chemical exposure on weight or body fat loss, or on changes in glycaemic control metrics resulting from an intentional weight loss diet.

Paper	Outcome	Chemical	Effect
Liu et al 2018	Weight change during 6 months weight loss diet (kg)	PFOS	No significant difference in estimated least squares mean between PFOS tertiles when adjusted for confounds (p = 0.27) and when TH were also accounted for (p = 0.63) or when PFOS was treated as continuous variable in each model (p=0.99; p =0.59, respectively).
		PFOA	No significant difference in estimated least squares mean between PFOA tertiles when adjusted for confounds (p = 0.73) and when TH were also accounted for (p = 0.41) or when PFOA was treated as continuous variable in each model (p=0.80; p =0.39, respectively).
		PFHxS	No significant difference in estimated least squares mean between PFHxS tertiles when adjusted for confounds (p = 0.76) and when TH were also accounted for (p = 0.54) or when PFHxS was treated as continuous variable in each model (p = 0.45; p = 0.22, respectively).
		PFNA	No significant difference in estimated least squares mean between PFNA tertiles when adjusted for confounds (p = 0.18) and when TH were also accounted for (p = 0.35) or when PFNA was treated as continuous variable in each model (p = 0.29; p = 0.54, respectively).
		PFDA	No significant difference in estimated least squares mean between PFDA tertiles when adjusted for confounds (p = 0.49) and when TH were also accounted for (p = 0.56) or when PFDA was treated as continuous variable in each model (p = 0.45; p = 0.76, respectively).
	Resting metabolic rate (RMR) change during	PFOS	Estimated least squares mean RMR was 6.9 ± 15.6 kcal day^{-1} in the lowest PFOS tertile versus -48.6 ± 14.8 kcal day^{-1} in the highest when adjusted for confounds (p <0.001). Lowest = -5 ±

	6 months weight loss diet (kcal/day)		16.3 kcal day^{-1} v -45.4 ± 15.5 kcal day^{-1} when TH were also accounted for (p = 0.005). Associations were significant when PFOS was treated as continuous variable in each model (p <0.001; p = 0.02, respectively) but effect sizes were not reported.
		PFOA	Estimated least squares mean RMR was not different in the lowest PFOA tertile versus highest when adjusted for confounds (p = 0.48) and even when TH were also accounted for (p = 0.61). Associations were not significant when PFOA was treated as continuous variable in each model (p = 0.37; p = 0.86, respectively).
		PFHxS	Estimated least squares mean RMR was -11.9 ± 15.4 kcal day^{-1} in the lowest PFHxS versus -42.3 ± 16.0 kcal day^{-1} in the highest when adjusted for confounds (p = 0.04). This difference was no longer apparent when TH were accounted for (p = 0.25). Associations were not significant when PFHxS was treated as continuous variable in each model (p = 0.20; p = 0.75, respectively).
		PFNA	Estimated least squares mean RMR was 8.1 ± 15.3 kcal day^{-1} in the lowest PFOS tertile versus -54.6 ± 15.1 kcal day^{-1} in the highest when adjusted for confounds (p <0.001). Lowest = -3.3 ± 16.1 kcal day^{-1} v -49.8 ± 15.9 kcal day^{-1} when TH were also accounted for (p = 0.002). Associations were significant when PFNA was treated as continuous variable in each model (p <0.001; p = 0.003, respectively) but effect sizes were not reported.
		PFDA	Estimated least squares mean RMR was -8.8 ± 15.7 kcal day^{-1} in the lowest PFDA tertile versus -43.1 ± 15.2 kcal day^{-1} in the highest when adjusted for confounds (p = 0.01). This difference was no longer significant when TH were also accounted for (p = 0.09). Associations were also significant when PFDA was treated as continuous variable in each model (p = 0.002; p = 0.05, respectively) but effect sizes were not reported.

	# Weight change during 6-24 months post diet (kg)	PFOS	Estimated least squares mean mass increase was 1.8 ± 0.6kg in the lowest PFOS tertile versus 3.3 ± 0.6 kg in the highest when adjusted for confounds (p = 0.009). Lowest = 1.5 ± 0.6 kg v 3.2kg when TH were also accounted for (p = 0.007). Associations were significant when PFOS was treated as continuous variable in each model (p =0.03; p = 0.02, respectively) but effect sizes were not reported.
		PFOA	Estimated least squares mean mass increase was not different in the lowest PFOA tertile versus highest when adjusted for confounds (p = 0.16) and even when TH were also accounted for (p = 0.12). Associations were not significant when PFOA was treated as continuous variable in each model (p = 0.07; p = 0.06, respectively).
		PFHxS	Estimated least squares mean mass increase was not different in the lowest PFHxS tertile versus highest when adjusted for confounds (p = 0.32) and even when TH were also accounted for (p = 0.18). Associations were not significant when PFHxS was treated as continuous variable in each model (p = 0.49; p = 0.21, respectively).
		PFNA	Estimated least squares mean mass increase was 2.0 ± 0.6 kg in the lowest PFNA tertile versus 3.4 ± 0.6 kg in the highest when adjusted for confounds (p = 0.01). Lowest = 1.8 ± 0.6 kg v 3.5 ± 0.6 kg when TH were also accounted for (p = 0.007). Associations were significant when PFNA was treated as continuous variable in each model (p = 0.01; p = 0.008, respectively) but effect sizes were not reported.
		PFDA	Estimated least squares mean mass increase was not different in the lowest PFDA tertile versus highest when adjusted for confounds (p = 0.16) but when TH were also accounted for, the difference between lowest and highest PFDA tertiles was significantly different (2.0 ± 0.6 kg vs 3.2 ± 0.6kg; p = 0.05). Associations were not significant when PFDA was treated as

		continuous variable in each model (p = 0.14; p = 0.06, respectively).
# Resting metabolic rate (RMR) change during 6-24 months post diet (kcal/day)	PFOS	Estimated least squares mean RMR was 108.3 ± 27.4 kcal day^{-1} in the lowest PFOS tertile versus 17.2 ± 26 kcal day^{-1} in the highest when adjusted for confounds (p <0.001). Lowest = 94.6 ± 27.5 kcal day^{-1} v -0.9 ± 26.2 kcal day^{-1} when TH were also accounted for (p < 0.001). Associations were significant when PFOS was treated as continuous variable in each model (p <0.001; p = 0.001, respectively) but effect sizes were not reported.
	PFOA	Estimated least squares mean RMR was 82.4 ± 26.9 kcal day^{-1} in the lowest PFOA tertile versus 27.7 ± 27.8 kcal day^{-1} in the highest when adjusted for confounds (p = 0.03). Lowest = 69.3 ± 27.3 kcal day^{-1} v -12.7 ± 28.1 kcal day^{-1} when TH were also accounted for (p = 0.03). Associations were significant when PFOA was treated as continuous variable in each model (p = 0.03; p = 0.04, respectively) but effect sizes were not reported.
	PFHxS	Estimated least squares mean RMR was 100.3 ± 27.1 kcal day^{-1} in the lowest PFHxS tertile versus 39.1 ± 28.3 kcal day^{-1} in the highest when adjusted for confounds (p = 0.02). Lowest = 81.5 ± 27.5 kcal day^{-1} v -24.6 ± 28.5 kcal day^{-1} when TH were also accounted for (p = 0.02). Associations were significant when PFHxS was treated as continuous variable in each model (p = 0.02; p = 0.04, respectively) but effect sizes were not reported.
	PFNA	Estimated least squares mean RMR was 83.8 ± 27.5 kcal day^{-1} in the lowest PFNA tertile versus 27.9 ± 27.4 kcal day^{-1} in the highest when adjusted for confounds (p = 0.03). Lowest = 73.7 ± 27.6 kcal day^{-1} v -14.1 ± 27.7 kcal day^{-1} when TH were also accounted for (p = 0.02). Associations were significant when PFNA was treated as continuous variable in each model (p = 0.004; p = 0.002, respectively) but effect sizes were not reported.

		PFDA	Estimated least squares mean RMR was 88.6 ± 27.7 kcal day^{-1} in the lowest PFDA tertile versus 40.3 ± 27.2 kcal day^{-1} in the highest when adjusted for confounds (p = 0.05). When TH were also accounted for the difference was no longer significant (p = 0.09). Associations were not significant when PFDA was treated as continuous variable in each model (p = 0.07; p = 0.08, respectively).
	Δ waist circumference (cm)	PFOS, PFOA, PFHxS, PFNA, PFDA	No significant association assessed by partial Spearman correlation co-efficient. ρ =-0.04 to 0.02.
	Δ visceral adipose mass (kg)	PFOS, PFOA, PFHxS, PFNA, PFDA	No significant association assessed by partial Spearman correlation co-efficient for PFOA (ρ =-0.14) or PFHxS (ρ =-0.11). Significant negative association with PFOS (ρ =-0.19), PFNA (ρ =-0.27) and PFDA (ρ =-0.26) but effect size is not reported.
	Δ total fat mass (kg)	PFOS, PFOA, PFHxS, PFNA, PFDA	No significant association assessed by partial Spearman correlation co-efficient. ρ =-0.08 to 0.06.
	Δ fasting glucose (mM)	PFOS, PFOA, PFHxS, PFNA, PFDA	No significant association assessed by partial Spearman correlation co-efficient. ρ =-0.08 to 0.04.
	Δ Insulin	PFOS, PFOA, PFHxS, PFNA, PFDA	No significant association assessed by partial Spearman correlation co-efficient. ρ =-0.03 to 0.03.
Ustulin et al 2018	BMI	PM $_{2.5}$ µg m^{3-1}	Significant positive association. Estimate = 0.085 [0.054; 0.115] CI; (p < 0.001). Relationship remains significant even when cities with high exposure and low initial BMI ad BMI change are excluded
		PM $_{10}$ µg m^{3-1}	Significant positive association. Estimate = 0.043 [0.021; 0.064] CI; (p= 0.002). Relationship remains significant even when cities with high exposure and low initial BMI and BMI change are excluded
		Pollution index x time	Significant positive association such that those with higher pollution exposure lose fewer BMI points over time. Estimate = 1.5 x 10^{-5} [9.01 x 10^{-6}; 2 x 10^{-5}] CI; (p <0.0001).
van der Meer et al 2021	BMI	Methyl paraben (MeP)	Significant positive association. Estimate = 0.11 [0.02; 0.21] CI; (p= 0.0234; q = 0.0469). Remains significant when those with T2DM are removed

		Ethyl paraben (EtP)	No association. Estimate = 0.04 [-0.04; 0.13] CI (p= 0.3214; q = 0.4285).
		Propyl paraben (PrP)	Significant positive association. Estimate = 0.10 [0.02; 0.18]; p = 0.0188; q = 0.0469.) Remains significant when those with T2DM are removed.
		BPA	No association. Estimate = -0.02 [-0.18; 0.14] CI; (p= 0..7757; q = 0.7757).
		Mono-ethyl phthalate (MEP)	No association. Estimate = 0.07 [-0.04; 0.18] CI; (p= 0.2115; q = 0.3384).
		Mono-iso-butyl phthalate (MiBP)	No association. Estimate = 0.04 [-0.15; 0.23] CI; (p= 0.6555; q = 0.6555).
		Mono-n-butyl phthalate (MnBP)	No association. Estimate = 0.15 [-0.05; 0.36] CI; (p= 0.1456; q = 0.3310).
		Mono-(2-ethylhexyl) phthalate (MEHP)	No association. Estimate = 0.07 [-0.12; 0.26] CI; (p= 0.4505; q = 0.5148).
		Mono-(2-ethyl-5-hydroxyhexyl) phthalate (MEHHP)	No association. Estimate = 0.21 [-0.02; 0.45] CI; (p= 0.0793; q = 0.331).
		Mono-(2-ethyl-5-oxohexyl) phthalate (MEOHP)	No association. Estimate = 0.11 [-0.10; 0.32] CI; (p= 0.3056; q = 0.4074).
		Mono-(2-ethyl-5-carboxypentyl) phthalate (MECPP)	No association. Estimate = 0.19 [-0.05; 0.44] CI; (p= 0.1254; q = 0.331).
		Mono-benzyl phthalate (MBzP)	No association. Estimate = 0.11 [-0.05; 0.27] CI; (p= 0.1655; q = 0.331).
	Waist circumference (cm)	Methyl paraben (MeP)	No association. Estimate = 0.26 [0.05; 0.56] CI; (p= 0.1039; q = 0.2078).
		Ethyl paraben (EtP)	No association. Estimate = 0.13 [-0.14; 0.40] CI (p= 0.3341; q = 0.2078).
		Propyl paraben (PrP)	No association after adjustment for multiple testing. Estimate = 0.29 [0.03; 0.15]; p = 0.0280; q = 0.1121)

		BPA	No association. Estimate = 0.06 [-0.43; 0.56] CI; (p= 0..8045; q = 0.8045).
		Mono-ethyl phthalate (MEP)	No association. Estimate = 0.25 [-0.09; 0.59] CI; (p= 0.1531; q = 0.3062).
		Mono-iso-butyl phthalate (MiBP)	No association. Estimate = 0.37 [-0.23; 0.97] CI; (p= 0.2312; q = 0.322).
		Mono-n-butyl phthalate (MnBP)	No association after adjustment for multiple testing. Estimate = 0.61 [0.06; 1.16] CI; (p = 0.0315; q = 0.1258).
		Mono-(2-ethylhexyl) phthalate (MEHP)	No association. Estimate = 0.00 [-0.58; 0.58] CI; (p= 0.9995; q = 0.9995).
		Mono-(2-ethyl-5-hydroxyhexyl) phthalate (MEHHP)	No association. Estimate = 0.58 [-0.14; 1.30] CI; (p= 0.1179; q = 0.3062).
		Mono-(2-ethyl-5-oxohexyl) phthalate (MEOHP)	No association. Estimate = 0.38 [-0.26; 1.02] CI; (p= 0.2415; q = 0.322).
		Mono-(2-ethyl-5-carboxypentyl) phthalate (MECPP)	No association. Estimate = 0.36 [-0.37; 1.09] CI; (p= 0.3377; q = 0.3859).
		Mono-benzyl phthalate (MBzP)	Significant positive association . Estimate = 0.68 [0.21; 1.16] CI; (p = 0.0053; q = 0.0424). Remains significant when those with T2DM are removed.
	% body fat	Methyl paraben (MeP)	No association. Estimate = -0.11 [-0.39; 0.17] CI; (p= 0.4445; q = 0.8890).
		Ethyl paraben (EtP)	No association. Estimate = -0.03 [-0.27; 0.21] CI (p= 0.7992; q = 0.8903).
		Propyl paraben (PrP)	No association. Estimate = 0.02 [-0.21; 0.25]; p = 0.8903; q = 0.8903)
		BPA	No association. Estimate = 0.19 [-0.26; 0.64] CI; (p= 0..4049; q = 0.8890).
		Mono-ethyl phthalate (MEP)	No association after adjustment for multiple testing and after removal of people with T2DM. Estimate = 0.32 [0.01; 0.62] CI; (p= 0.0438; q = 0.1752).

		Mono-iso-butyl phthalate (MiBP)	No association. Estimate = 0.28 [-0.26; 0.83] CI; (p= 0.3124; q = 0.3982).
		Mono-n-butyl phthalate (MnBP)	No association after adjustment for multiple testing. Estimate = 0.43 [-0.08; 0.93] CI; (p = 0.0994; q = 0.2651).
		Mono-(2-ethylhexyl) phthalate (MEHP)	No association. Estimate = -0.25 [-0.77; 0.27] CI; (p= 0.3484; q = 0.3982).
		Mono-(2-ethyl-5-hydroxyhexyl) phthalate (MEHHP)	No association. Estimate = 0.39 [-0.26; 1.05] CI; (p= 0.2421; q = 0.3874).
		Mono-(2-ethyl-5-oxohexyl) phthalate (MEOHP)	No association. Estimate = 0.23 [-0.35; 0.81] CI; (p= 0.4411; q = 0.4411).
		Mono-(2-ethyl-5-carboxypentyl) phthalate (MECPP)	No association. Estimate = 0.50 [-0.16; 1.17] CI; (p= 0.1399; q = 0.2799).
		Mono-benzyl phthalate (MBzP)	Significant positive association. Estimate = 0.62 [0.19; 1.05] CI; (p = 0.0053; q = 0.0424). Association remains significant when those with T2DM are removed.
Grandjean et al 2023	Relative weight loss	PFAS (PFHxS, PFOA, PFOS, PFNA, PFDA)	Weak association of each chemical measured (p> 0.07). No further detail on effect size provided or on breakdown of different congeners
	*Measured absolute weight loss (kg) tertiles: 5.6-9.2kg v 11.8-28.3kg	PFHxS (ng/ml)	0.6 [0.3,1.0] v 0.8 [0.4-1.3]; p = 0.03
		PFOS (ng/ml)	9.3 [6.1-14.2] v 10.3 [7.1-16.7]; p = 0.11
		PFOA (ng/ml)	2.4 [1.5,3.4] v 2.8 [1.8-4.3]; p = 0.02
		PFNA (ng/ml)	0.7 [0.5-0.9] v 0.8 [0.6-1.1]; p = 0.09
		PFDA (ng/ml)	0.3 [0.2,0.3] v 0.3 [0.2,0.3]; p = 0.88
	# Predicted absolute weight regain (kg) after 26 weeks of weight maintenance	PFHxS (ng/ml)	0.906kg (0.544 -1.268) per doubling; p <0.001.
		PFOS (ng/ml)	1.015kg (0.431 - 1.598) per doubling; p <0.001
		PFOA (ng/ml)	1.496kg (0.883 to 2.110) per doubling; p <0.001
		PFNA (ng/ml)	1.104 (0.351 to 1.857) per doubling; p <0.001
		PFDA (ng/ml)	1.059 (0.176 to 1.943) per doubling; p 0.02

	following low calorie diet	Sum PFAS (ng/ml) adjusted for within couple dependence	1.71kg (0.86-2.56) per doubling; $p < 0.001$
Kahleova et al 2016	ΔHb1Ac (%)	Sum Δ POPs (pg g^{-1}): of the 24 measured the following showed significant associations: OCDD; 2,3,7,8 TCDF$^{\$}$; 2,3,4,7,8-PeCDF; WHO-PCDD/ F-TEQ; CB77$^{\$}$,118$^{\$}$,123$^{\$}$,114; 105$^{\$}$;126$^{\$}$; 167$^{\$}$; 156$^{\$}$;157$^{\$}$;169$^{\$}$; 189	Positive correlation R^2= 0.34; $p < 0.01$ with little change in co-efficients after adjustment for ΔBMI or Δ visceral fat. Individual regression co-efficients for each congener (when all others are held constant) varied between 0.02 and 0.06. POP predictors explained 10.6% of variability in ΔHb1Ac (2% after cross validation).
	Δ Fasting plasma glucose (mM)	Sum Δ POPs (pg g^{-1}): of the 24 measured the following showed significant associations: OCDD; 2,3,7,8 TCDF$^{\$}$; 2,3,4,7,8-PeCDF$^{\$}$; WHO-PCDD/ F-TEQ$^{\$}$; CB77$^{\$}$,118$^{\$}$,123,114$^{\$}$; 105$^{\$}$;126$^{\$}$; 167$^{\$}$; 156$^{\$}$;157$^{\$}$;169$^{\$}$; 189$^{\$}$	Positive correlation R^2 = 0.41; $p < 0.01$ with little change in co-efficients after adjustment for Δ BMI or Δ visceral fat. Individual regression co-efficients for each congener (when all others are held constant) varied between 0.02 and 0.05. POP predictors explained 16.9% of variability in Δ fasting glucose (12.6 % after cross validation).
	Δ β-cell function (assessed as modelled insulin secretion at reference glucose level after standard meal test)	Sum Δ POPs (pg g^{-1}): of the 24 measured the following showed significant associations: OCDD; 2,3,7,8 TCDF$^{\$}$; 2,3,4,7,8-PeCDF$^{\$}$; WHO-PCDD/ F-TEQ$^{\$}$;	Negative correlation R^2 = -0.37; $p < 0.01$ with little change in co-efficients after adjustment for Δ BMI or Δ visceral fat. Individual regression co-efficients for each congener (when all others are held constant) varied between -0.01 and -0.05. Explained 13.4% of variability in Δ β-cell function (10 % after cross validation).

		CB77$, 118$, 123, 114$; 105$; 126$; 167$; 156$; 157$; 169$; 189$	
	Δ whole body insulin sensitivity (assessed using metabolic clearance rate of glucose in last 20 mins of 3 h hyperinsulinaemic (1 mU kg^{-1} min^{-1}) isoglycaemic clamp, after adjusting for changes in glucose pool size)	24 measured POPs	No correlation
	Δ mass	24 measured POPs	Unreported
	Δ BMI	24 measured POPs	Unreported – used to adjust for changes in other metabolic parameters in OPLS
	Δ visceral fat volume	24 measured POPs	Unreported – used to adjust for changes in other metabolic parameters in OPLS
Imbeault et al 2002 a	Baseline fasting insulin (pmol/l)	OCs (CB53; Aroclor 1260; p-p'-DDE; β-HCH and HCB)	No correlation in men or women (r = -0.2 to 0.29; p >0.05). No further details or data are reported to determine effect size
	Baseline AUC for insulin during OGTT		No association in men; positive association in women for HCB, β-HCH Aroclor 1260 and CB153 (r = 0.49 to 0.67; p < 0.05) but not p-p'-DDE. No further details or data are reported to determine effect size.
	Δ fasting insulin during weight loss (pmol/l)	Δ p,p -DDE	r = -0.49 in men; r = -0.17 in women. Partial correlation when adjusted for fat content is NS (r =-0.32).No further details or data
		Δ β-HCH	r = -0.53 in men; r = 0.10 in women. Partial correlation when adjusted for fat content is NS (r =-0.39).No further details or data

	Δ HCB	r = -0.57 in men; r =- 0.27 in women. Partial correlation when adjusted for fat content remains significant (r =-0.51; p <0.05). No further details or data
	Δ Aroclor 1260	r = -0.59 in men; r = -0.15 in women. Partial correlation when adjusted for fat content remains significant (r =-0.51; p <0.05). No further details or data
	Δ CB153	r = -0.57 in men; r = -0.22 in women. Partial correlation when adjusted for fat content is NS (r =-0.46; p =0.07). No further details or data
Post diet AUC for insulin during OGTT	Δ OCs (CB53; Aroclor 1260; p-p'-DDE; β-HCH and HCB)	No association in men or women (r =-0.41 to -0.08; p >0.05). No further details or data
Post diet fasting glucose (units not reported)	Δ OCs (CB53; Aroclor 1260; p-p'-DDE; β-HCH and HCB)	No association in men or women (r =-0.25 to -0.41; p >0.05). No further details or data
Post diet AUC for glucose	Δ OCs (CB53; Aroclor 1260; p-p'-DDE; β-HCH and HCB)	No association in men or women (r =-0.25 to -0.041; p >0.05). No further details or data

Table S10: Covariates and potential confounds

	Liu et al 2018		Ustulin et al 2018		Van der Meer et al 2021		Grandjean et al 2023		Kahleova et al 2016		Imbeault et al 2002	
	R	A	R	A	R	A	R	A	R	A	R	A
Age	Y	Y	Y	Y	Y	Y	Y	Y	Y	N	Y	N
Sex	Y	Y	Y	Y	Y	Y	Y	Y	Y	N	Y	Y
Ethnicity	Y	Y	N	-	N	-	N	-	N	-	Y	NA
Educational attainment	Y	Y	N	-	N	-	N	-	N	-	N	-
Occupation	N	-	N	-	N	-	N	-	N	-	N	-
Socio-economic group	Y	N	N	-	N	-	N	-	N	-	N	-
Smoking status	Y	Y	N	-	P	N	N	-	Y	N	P	N
Alcohol consumption	Y	Y	N	-	P	N	E	NA	Y	N	P	N
Habitual exercise/ activity levels	Y	Y	U	-	N	-	N	-	N	-	Y	N
Exercise/ activity during diet intervention	Y	Y	N	-	U	N	Y	U	N	-	Y	N
Menopause status	Y	Y	N	-	N	-	N	-	N	--	N	-
HRT status	Y	Y	N	-	N	-	N	-	N		N	-
Stability of weight prior to diet	N	-	N	-	Y	NA	Y	NA	N	-	Y	NA
Concurrent diabetes medications	NA	-	N	-	N?	-	E	NA	Y	N	NA	-
Concurrent other medications	E	NA	N	-	N?	-	E	NA	Y	N	E	NA
T1D	E	NA	N	-	E	NA	E	NA	E	NA	E	NA

T2D	E	NA	N	-	Y	Y	E	NA	Y	NA	E	NA
T2D duration	NA	NA	N	-	N	N	NA	NA	N		NA	NA
T2D severity	NA	NA	N	-	N	N	NA	NA	Y	NA	NA	NA
Urban vs rural residence	N	-	Y	NA	N	-	N	-	N	-	N	-
Initial adiposity or resting metabolic rate	Y	Y	Y	N	Y	Y	Y	U	Y	N	Y	Y?
Adherence to diet	Y	Y	U	U	Y	N	E	NA	Y	N	N	-
Leptin levels	Y	Y	N	-	N	-	N	-	Y	N	N	-
Thyroid hormone levels	Y	Y	N	-	N	-	E	NA	N	-	N	-
Normal diet	Y	N	N	-	N	-	N	-	Y	N	N	-

Covariates and potential confounds that were reported (R) either in the paper in question or a previous report from the same study that is cited in the paper, and/ or included in the analysis (A) of the six papers that were critically evaluated in detail. E = specifically excluded; Y = yes; N = no; P = partly; U = unclear; NA = not applicable because the variable was constant/ limited or excluded

Table S11: Congeners (IUPAC number) measured in the two studies that examined PCBs and methodological considerations.

Paper (first author/year)	Method	Lipid	77	81	105	114	118	123	126	138	153	156	157	167	169	180	189
Imbeault (2002)	Plasma spiked with PCB198 hexane: acetone extracted and defatted with sulphuric acid. Water and KOH washes preceded sodium sulphate filtration. Florisil clean-up of centrifuged plasma, elution solvent unreported. Detection using GC with dual capillary columns and dual ^{63}Ni detectors (HP-5890) at Québec Toxicology Center, Canada.	y									x						
Kahleova (2016)	Homogenised plasma spiked with a dioxin and unspecified dioxin -like PCB standard. Sample mixed with sodium sulphate and methylene- chloride hexane mix and extracted in a Soxtec extractor. Sample clean up performed using silica gel and dual layer carbon-reversible column followed by evaporation to dryness and resuspension in nonane. Internal standards added to each extract immediately prior to injection. Detection performed by isotope dilution using HRGC/ HRMS. Multi point calibration, spiked samples and standards tested for QC. Non dioxin like PCBs analysed in serum. Sample mixed with sodium sulphate and methylene-chloride acetone mix, extracted and filtered. Extract diluted in cyclohexane and cleaned by gel permeation chromatography then concentrated to near dryness and reconstituted in isooctane and measured by GC on high-resolution capillary columns (DB-5 and DB-17) and detected using a microcell electron capture detector and calibration performed with external standard.	n	x	x	x	x	x	x	x	x	x	x	x	x	x	x	x

Lipid indicates whether reported values were lipid corrected (y) or not (n). x indicates where that congener was measured. GC = gas chromatography. GC-MS = gas chromatography linked to mass spectrometry. HRGC = high-resolution gas chromatography; HRMS high-resolution mass spectrometry

Table S12: Risk of bias detail. Risk of bias for the six studies that addressed the potential association between contaminant exposure and weight loss or glycaemic control for each of seven domains indicated in the lefthand column. Each cell provides a synopsis of consensus responses to signal questions within each domain (see **Table S3**) and allow comparison between studies for each aspect considered.

	**Liu et al (2018)**	**Ustilin et al (2018)**	**Van der Meer et al (2021)**	**Grandjean et al (2023)**	**Kahleova et al (2016)**	**Imbeault et al (2002)**
1. Confounds and covariates	Low/no concern: Measured, recorded and accounted for many covariates (Table S10). Activity and food source could be confounds.	High concern: recorded and accounted for small number of covariates. Confounds of higher exposure status, such as city of residence and daily weather patterns, and number of log ins were partly identified and some controlled for in the analysis. Lack of reporting of health status, especially T2DM, concurrent medications; socioeconomic group; occupation; menopause status in females; ethnicity were not reported and thus not included in later analysis. Lack of information on education may have a bearing on accuracy of recording. The authors report they were unable to extract these data.	Moderate concern: Lifestyle habits including normal diet; concurrent medications especially for diabetes management; socioeconomic group; occupation; menopause status in females; health status other than T1DM; T2DM duration; and ethnicity were not reported and thus not included in later analysis. Activity and food source could be confounds.	Low concern: Measured, recorded and accounted for some covariates (Table S10). Exclusion criteria largely controlled participants' characteristics and thus likely minimised confounds at the study outset. Socioeconomic group; occupation or educational attainment; menopause status or HRT use in females; typical diet or activity levels; activity during the dietary intervention; and ethnicity were not reported and thus not included in later analysis. Clinical	Moderate concern: Confounds of higher exposure status were partly identified and some controlled for in the analysis. Incomplete reporting or accounting for medications and diabetes duration. Concurrent antidiabetic medication was reported in terms of percentages in Kahleova et al (2011), but polypharmacy was not. Other medications (e.g. lipid lowering and blood pressure control medications) that may influence glycaemia, contaminant metabolism or weight loss were also	Low concern: Strict exclusion criteria that tightly controlled participants' characteristics and thus likely minimised the confounds at the study onset. Some confounds were identified and strategies to deal with these confounds were partly implemented, such as testing for differences between placebo and the appetite suppressing drug prior to further analysis. However, no data on socioeconomic group or other social inequality marker that can affect contaminant exposure and menopause

	Initial BMI was not used as a covariate. Activity and food source could be confounds. It is unclear how much the app may vary in its advice to people from different regions. Participants were nested in city which may partly account for this potential confound. However, not attempt made to group people by the type of advice received.		centre and whether the person came from a family with one or two obese adults were included as covariates, which allows for confounding effects across countries and where individuals share the same home influences.	reported as percentages. While this information was available it is not clear how it was used later in analysis. Medication use was not included as a covariate. It is unclear how the adherence data were incorporated into the analysis. Activity and food source could be confounds	status in women was not recorded despite the older range of participants. Activity and food source could be confounds.
No concern: Unclear if participants were weight stable at the outset but a run-in period of 2 weeks was used to standardise food intake at 100% energy requirements.	Moderate concern: Unclear if participants were weight stable at the outset.	Moderate concern: Those that had recently lost weight of more than 10% were excluded, but this is a very large reduction so some participants may have not been weight stable within those bounds	No concern: Those that had recently lost weight of more than 3kg were excluded	Moderate concern: Unclear if participants were weight stable at the outset.	No concern: Participants were weight stable at start.
Low concern: Activity level was accounted for in calorie requirements and was monitored, though how this was incorporated is not	High concern: frequency of physical activity was included as a covariate in Study 1 but it is unclear how this was recorded (number of logs or nature of activity). In study 2 this was not	Low concern: Exercise regime before and during diet was measured using a questionnaire in the original study and did not change with time individuals or	Low concern: Activity levels were not accounted for but those who were intending to change their activity regime during the study were excluded	Low concern: Participants were asked to maintain their normal activity patterns during the dietary intervention, and these were monitored such that	Low concern: Activity level was accounted for in calorie requirements, although this was not individualised. Participants reported to be sedentary, and

reported here or in Sacks et al (2009)	accounted for, but the mixed effects design may incorporate individual level differences in habitual activity. It is not clear how changes in activity by individuals over time was accounted for in study 2.	between groups, and group composition was based on even distribution of physical activity levels (Soenen et al 2012). Physical activity was thus unlikely to be a confound in weight loss trajectory.		exercise was not deemed a potential confound here.	this appears to be consistent between participants.
Low concern: All participants were on the same diet and no adjustments were made on individual or group basis	High concern: the Noom coach app provides individualised weight loss plans that include tailored dietary advice and such differences, especially when unaccounted for, could add substantial between individual diet interventions that may unintentionally change exposure status or result from differences in initial exposure. Such differences may be region specific. The model accounted for regional differences, but it is unclear how.	Moderate concern: Where amount of protein could not be achieved with the assigned menus in high protein diets, participants were given additional shakes, which would have increased calorie intake.	Low concern: All participants were on the same diet	Low concern: No adjustments to the diet made for individuals or in specific groups	Low concern: All participants were on the same diet and no adjustments were made on individual or group basis
Low concern: no difference between	High concern: it is not clear what the differences in	Moderate concern: People were given individual advice on	Low concern: no difference between	Moderate concern: Diet adherence was supported where	Low concern: no difference between

	individuals or groups in advice given	mindfulness/ behavioural coaching was between participants which could have created large disparities in advice given within and between cities.	diets, where needed, such that some differences could have arisen between participants that was not recorded or reported.	individuals or groups in advice given	required by counselling, such that not all participants received the same level of input and this was not accounted for.	individuals or groups in advice given
	Low concern: Although meal plans were provided, participants had to source the food for themselves, and these choices may have influenced chemical exposures, which may add unaccounted for variability	Low concern: Since participants sourced the food for themselves, these choices may have influenced other chemical exposures in addition to air pollution or actual calories consumed which may add unaccounted for variability	Low concern: Since participants had to source the food for themselves, these choices may have influenced chemical exposures or actual calories consumed which may add variability	Low concern: all participants had the same replacement diet and had strict limits to what it could be supplemented with	Low concern: food was provided in specific locations such that food intake and source was standardised	Low concern: Participants had to source the food for themselves, and these choices may have influenced chemical exposures or actual calories consumed, which may add variability
2. Participant selection	No concern: Clear and thorough selection criteria.	Low concern: all participants who signed up for the App and were not excluded for stated reasons were included and thus the study co-ordinators did not impose additional bias.	No concern: Clear and thorough selection criteria.	No concern: Clear and thorough selection criteria.	No concern: Clear and thorough selection criteria. Inclusion criteria were very specific, including criteria for diabetes diagnosis, and were provided in a previous paper (Kahleova et al 2011).	No concern: Clear selection criteria.
	Low concern: Recruitment by mass mailing assumed to	Moderate concern: Access to a Smart phone and being comfortable	Low concern: Access to healthcare providers and ability	Moderate concern: Access to healthcare providers and ability	Low concern: Subjects were referred from an	Low concern: Recruitment by mass mailing was assumed

be representative of the general population. Source of mailing lists was provided (commercial vendors and local governments for lists of registered voters or drivers). Secondary methods included advertisements on buses and subways, worksite advertisements, newspaper advertisements, recruitment flyers, and mail to local healthcare centres and businesses such that the sample was recruited using a wide range of approaches likely to minimise bias. Further detailed information on participants and recruitment was available in Sacks et al (2009).	with the use of this technology, sharing data and comfort level with cognitive behavioural therapy approaches on which Noom is based may have influenced the representativeness of study participants. The degree of marketing of Noom in different locations and the target audience of the App likely has a substantial influence. This is reflected in the very narrow age range of study participants. In addition, a highly variable percentage of people who had a BMI <25 was reported, which is much more variable and much greater than the other studies, which largely targeted overweight and obese people. The larger proportions of 'normal weight' BMI categories (between 11 and 67%) may influence the ability to detect a change in	to attend clinics may have influenced the representativeness of the sample in this study. Participants were referred by their general practitioner or specialist at weight management programs in outpatient-clinics and this represents people already seeking help with weight loss rather than the wider population and may also produce bias towards selection of those demographics with greater health seeking behaviours or access to health care, which can correlate with exposure status.	to attend clinics that may have influenced the representativeness of the sample in this study was offset by recruitment via mass mailing, although the proportion of each is not reported. However, of greater concern is that to be included in the weight regain study, people had to have lost 8% or more of their original body mass such that people who had more limited weight loss in the low calorie phase are not reported in this subset of the original study and thus we have no information on their PFAS levels.	endocrinologist. It is unclear whether this may affect selection since endocrinologists may refer the people who are struggling most to lose weight or regain normoglycaemia. Information on whether all patients who met the inclusion criteria were referred, and whether this was sequential, was not provided. People were already seeking help with weight loss rather than the wider population and this may produce bias towards selection of those demographics with greater health seeking behaviours or access to health care, which can correlate with exposure status.	to be representative of the general population but recruitment of only Caucasians could represent some bias in initial selection that is difficult to identify from the description. Approaches used that minimised the opportunity to unintentionally pre-select specific demographics at recruitment. Some omissions still present but not considered substantial eg. age was not specified in selection criteria; details given on exclusion criteria were adequate in terms of disease status although which endocrine disorders were excluded are not specified; women's pregnancy, breastfeeding, menopausal and HRT status were not

Approaches used that minimised the opportunity to unintentionally pre-select specific demographics at recruitment. Some omissions still present but not considered substantial e.g. not clear exactly which medications were excluded and what was deemed as sufficient motivation.	mass as well as reflecting highly weight and/ or health-conscious attitudes decoupled from actual adiposity. No further demographic information is provided				reported, and the way in which diabetes was tested for and thus excluded was not clear.
Low concern: Allocation concealment is unclear here and in Sacks et al (2009). Randomisation to treatment was performed by a data manager to ensure an even number of people in each of four diet groups at the different study sites, such that assignment to group may not have been	No concern: there is no allocation concealment because each person is provide with advice on the basis of their input details which the study authors do not appear to have had access to	Low concern: Allocation concealment is unclear here and in Soenen et al (2012). Allocation to treatment group could have occurred but was not likely to have substantially affected results. Blinding of experimenters to diet group is unclear because participants were not assigned in a	Low/ no concern: all participants were on one diet for the initial weight loss phase and randomisation to the later weight regain phase diet is not relevant.	Low concern: Allocation concealment is unclear here and in Kahleova et al (2011). Non-random allocation to treatment group could have occurred but was not likely to have substantially affected results.	Moderate concern: Allocation concealment is unclear. How participants were assigned to placebo or appetite suppressant groups is not reported, such that randomisation is unclear. The data reported are stratified by sex rather than treatment in the tables making it impossible to

completely random. However, randomisation to diet groups is unlikely to have affected the study outcome assessing impact of contaminant on weight loss parameters		random way but were assigned on basis of sex, BMI and activity. The intervention groups were thus artificially well balanced, but this is unlikely to affect the assessment of contaminant exposure on weight loss.			determine whether there was a difference in contaminant levels at the start of the intervention between placebo and fenfluoramine treated individuals. The study was not balanced in terms of placebo and treatment and allocation to treatment not explained or clearly blinded. However, the authors report no treatment effects on POP levels, suggesting no selection bias in allocation of people to groups that could have affected the outcome of interest.
No concern: Blinding of participants to diet allocation was maintained by use of similar foods (Sacks et al 2009)	Low concern: participants had downloaded the app, signalling their intent to lose weight and could have compared the advice received with other users but it is unclear how such lack of blinding would affect	Low concern: Participants were given detailed macronutrient advice, such that they likely knew or could calculate what they were trying to increase or decrease in the diet and thus	No concern: all participants were on diet such that no blinding was required	Low concern: Blinding of participants to treatment is not reported, but it would have been clear who was assigned to a conventional versus vegetarian diet and it was not clear how participant contact	Low concern: It is not clear if blinding of participants to treatment with appetite suppressant occurred, but such blinding is unlikely to have influenced outcomes in regard to

		weight loss. There was no intentional allocation to different regimes and to compare them in this study	the blinding process for dietary intervention was not adequate. However, such lack of blinding may have affected their motivation, but is unlikely to have affected the outcome of the post hoc analysis about contaminant exposure.		between groups was managed. This suggests blinding to treatment did not occur and this may have affected motivation to adhere to the diet. Nevertheless, this potential for lack of blinding is unlikely to be associated with contaminant status, such that risk of bias was not considered significant.	contaminant exposure.
3. Diet intervention	Low concern: Diet duration was clearly reported and was sufficiently long for differences in mass loss trajectory or glycaemic control between participants to be seen.	Moderate concern: Although App users had to have used the App for at least 12 months, it is not clear how much variation there was between participants in diet duration, which could affect total change in BMI.	Low concern: Diet duration was clearly reported and was sufficiently long for differences in mass loss trajectory or glycaemic control between participants to be seen	Low concern: Diet duration was clearly reported and was sufficiently long for differences in mass loss trajectory to be seen. Positive glycaemia control effects reported in Goyenechea et al (2011).	Low concern: Diet duration was clearly reported and was sufficiently long for differences in mass loss trajectory or glycaemic control between participants to be seen	Low concern: Diet duration was clearly reported and was sufficiently long for differences in mass loss trajectory or glycaemic control between participants to be seen
	Low concern: Diet described in detail in Sacks et al (2009).	High concern: no information is given on the nature of the diets that people were assigned and how much this differed between	Moderate concern: Total diet composition is given in Soenen et al (2012), with some information on what food stuffs were	Low concern: Diet described in detail in Goyenechea et al (2011)	Low concern: Information on the diets provided in some detail in Kahleova et al (2011), including balance of	High concern: No detail on the nature of the diet, particularly composition by macronutrient group/ specific food stuffs, or

	participants or regions/ cities	included, but more detail on specific menus is not provided. Participants appear to have been given food menus, but not the food itself.		carbohydrates, proteins and fats, and the nature of specific food stuffs included.	setting in which they were eaten was provided.
Low concern: Possibility that participants had the opportunity to deviate from the diet and for that to go unrecorded or unaccounted for. However, details of the diets were extensive and information on compliance was reported and at least partially used in analysis. Participants were provided with 2 weeks of daily meal plans in blocks and had group sessions to support and monitor adherence alongside a web-based food diary monitoring tool. Parameters on	High concern: Possibility that participants had the opportunity to deviate from the diet and for that to go unrecorded or unaccounted for since all monitoring is self-reported and on a self-regulated schedule that can differ between people. The degree to which participants differed in regularity of information input including weight and food intake is unclear. The calorie estimation provided by the app and the time over which calorie intake was estimated is not provided and could have differed between people. However, 80% of users lost weight, suggesting	Moderate concern: Possibility that participants had the opportunity to deviate from the diet and for that to go unrecorded or unaccounted for. Adherence was monitored through weekly clinic assessments made in the first month, every 2 weeks over the next 3 months and monthly during the last eight months of the original study (which was not reported on in van der Meer et al 2021). In addition, there were group sessions to support the diet and healthy eating. What was required of	Moderate concern: Possibility that participants had the opportunity to deviate from the diet and for that to go unrecorded or unaccounted for. No information on adherence monitoring in the low calorie phase of the study is reported. However only those who successfully lost >8% body mass were included in this analysis and thus must have adhered to the diet.	Low concern: Possibility that participants had the opportunity to deviate from the diet and for that to go unrecorded or unaccounted for. However, details of the diets were extensive and information on compliance was reported and at least partially used in analysis. Compliance was monitored: meals were provided in a restaurant and their collection monitored. Diet diaries of representative weekdays and weekend were recorded and checked by a dietician and	High concern: Possibility that participants had the opportunity to deviate from the diet and for that to go unrecorded or unaccounted for. Compliance assessment and parameters for completion of the diet programme were undescribed. Doucet et al (2001), which contains more detail on the regime used here, includes information on dietitian visits but their regularity, how these were monitored and whether compliance was included in the analysis is unclear.

completion and adherence were only partly reported in Sacks et al (2009) and it was not clear how this information was used in the later analysis in Liu et al (2018).	some adherence by most participants	participants to remain in the programme in terms of attendance is not reported. Soenen et al (2012) reports dropouts and these are people who could not attend the clinics, but it is not clear how many absences were tolerated.<br><br>Attendance at clinical check-ups and adherence is not reported for the participants included in the van der Meer et al (2021) paper, which only contains those people for whom the start- and end -of -diet urine samples were available, making the data from the earlier study not directly usable.		follow up phone calls were made to monitor compliance. Cholesterol levels were also monitored to assess compliance. The adherence data were used to assign participants to high, medium, and low groups according to excess calories consumed, and participants had to meet energy intake and cholesterol level cut offs to be considered adherent and the % adherence is reported. Adherence in the different diet types is reported in Kahleova et al (2011).	
Low concern: Measured RMR directly and used this as the basis of the calorie content of the meals prescribed.	Moderate concern: Individualised plans based on participant-entered details likely include assumptions about activity and metabolic rate but how	Moderate concern: Assumptions about both BMR (based on generic equation) and physical activity (simple 1.5 multiplier applied) that may	Moderate concern: all participants were given the same diet irrespective of individual requirements, which may contribute to	Low concern: Measured RMR directly and used this as the basis of the calorie content of the meals prescribed.	Moderate concern: Caloric intake was based on an initial direct measurement of resting metabolic rate, with a multiplier of 1.4 applied to

	this is calculated is not reported	have affected whether the diet was truly isocaloric between participants.	variability in weight loss trajectory		account for physical activity.
Low concern: Physical activity was assumed to be 90 minutes moderate activity per week and was assessed using standardised questionnaires to control for activity.	Moderate concern: for study 1 activity level was accounted for and based on monitoring from the individual's device. Any inaccuracies are thus due to technical issues related to device registration of steps/ movement and whether the individual has the device on their person when moving, rather than on recollection/ self-reporting. It is unclear how much control users have over the reporting of this data to the app and what information is used. In study 2 activity does not appear to be accounted for, but inter individual differences are absorbed in the random effect of user.	Low concern: Physical activity was measured using a questionnaire and did not change over the study between people or groups and is thus unlikely to account for differences in weight loss trajectory.	Moderate concern: activity levels were not accounted for	Low concern: Physical activity was monitored using a pedometer for two weekdays and a weekend at baseline, evaluated using a standardised questionnaire and participants were instructed not to change their normal activity patterns during the 12-week weight loss phase:	Moderate concern: assumptions made about activity levels based on self reporting, but these were not measured or accounted for
Low concern: Unclear whether intermittent fasting	Low concern: Unclear whether intermittent fasting was imposed or	Low concern: Unclear whether intermittent fasting was imposed	Low concern: Unclear whether intermittent fasting was imposed	Low concern: Unclear whether intermittent fasting was imposed	Low concern: Unclear whether intermittent fasting was imposed

	was imposed or could have been followed by some but not all participants because timing of meals, which could affect weight loss and glycaemic control, was not restricted or reported.	could have been followed by some but not all participants because timing of meals, which could affect weight loss and glycaemic control, was not restricted, or reported.	or could have been followed by some but not all participants because timing of meals, which could affect weight loss and glycaemic control, was not restricted or reported.	or could have been followed by some but not all participants because timing of meals, which could affect weight loss and glycaemic control, was not restricted or reported.	or could have been followed by some but not all participants because timing of meals, which could affect weight loss and glycaemic control, was not restricted or reported.	or could have been followed by some but not all participants because timing of meals, which could affect weight loss and glycaemic control, was not restricted or reported.
4. Exposure measurement	No concern: Employed a direct method of exposure assessment from a sample taken from the person using an appropriate and validated matrix.	High concern: In study 1 exposure was taken as the annual exposure based on location and the granularity in the database cited seems to be a single value for each PM, which suggests every person in each city would be assigned the same exposure value. In study 2 the data were more granular and adjusted for weather, such that every daily user location should have a corresponding air quality index comprising an algorithm derived from a combination of ozone, $PM_{2.5}$ and PM_{10} and their corresponding	No concern: Employed a direct method of exposure assessment from a sample taken from the person using an appropriate and validated matrix.	No concern: Employed a direct method of exposure assessment from a sample taken from the person using an appropriate and validated matrix.	No concern: Employed a direct method of exposure assessment from a sample taken from the person using an appropriate and validated matrix.	No concern: Employed a direct method of exposure assessment from a sample taken from the person using an appropriate and validated matrix.

	health risks. These data may suffer from issues with temporality.				
No concern: Single point sample, which is appropriate given the long half-life of PFAS. Reported on how reproducible within-person blood measures of PFAS were in a pilot study, which helps to ascertain that snapshot measurements were appropriate for this chemical group.	High concern: values are annual average in study 1 and a composite value in study 2 such that the measures in each case are very indirect and do not take account of very localised sources of exposure and behaviour patterns, including occupational or indoor exposures	Low concern: 24 h urine samples taken and mixed prior to measurement to provide an average. While day to day differences may exist, this approach is considered standard for the chemical groups measured here.	No concern: Single point sample, which is appropriate given the long half-life of PFAS. Reported on how reproducible within-person blood measures of PFAS were in a pilot study, which helps to ascertain that snapshot measurements were appropriate for this chemical group.	No concern: Single point sample, which is appropriate given the long half-life of POPs.	No concern: Single point sample, which is appropriate given the long half-life of POPs.
Low concern: Suite of chemical compounds within the target chemical group measured.	Moderate concern: two or three major air pollutants of health concern are considered but in study 2 this is a composite measure	Low concern: Suite of chemical compounds within the target chemical group measured.	Low concern: Suite of chemical compounds within the target chemical group measured.	Low concern: Suite of chemical compounds within the target chemical group measured.	Low concern: Suite of chemical compounds within the target chemical group measured.
No concern: Limited potential for knowledge of the outcome measurements to have influenced the measurement of exposure.	No concern: since data are amalgamated from a variety of external providers, the study coordinators here could not have influenced the exposure measurements	No concern: Limited potential for knowledge of the outcome measurements to have influenced the measurement of exposure.	No concern: Limited potential for knowledge of the outcome measurements to have influenced the measurement of exposure.	No concern: Limited potential for knowledge of the outcome measurements to have influenced the measurement of exposure.	No concern: Limited potential for knowledge of the outcome measurements to have influenced the measurement of exposure.

Low concern: Reagent and serum blanks were measured (Vestergaard et al 2012), which should account for any contamination of blood tubes or other plasticware used in collection and analysis.	Low concern: measurements in study 1 are provided by WHO from annual monitoring programmes with transparent regimes for data recording. In study 2 the AQI data are provided by the US Environmental Protection Agency and the raw data are acquired by EPA approved state, local or tribal monitoring agencies that use federal reference or equivalent monitoring methods	Moderate concern: It is not clear how the authors ensured that the collection of urine was phthalate or BPA free since this was not the initial intended use of the urine collected	Moderate concern: It is not clear how the authors ensured that the collection of blood was PFAS free since this was not the initial intended use of the blood collected because information on procedural blanks is not specifically stated.	Moderate concern: sampling and storage was unlikely to have introduced contamination, but standards were not used for all the POPs measured and POP levels were not lipid corrected.	Moderate concern: sampling and storage was unlikely to have introduced contamination, but only one PCB standard was used (PCB198), which was not the target analyte
No concern: Timing of baseline measurement reported and aligned with other measurements.	High concern: for study 1 where annual values are used, the timing of start and end of the diet period will vary for each person. Study 2 suffers from temporality issues since the pollution AQI associated with the log in day is used such that BMI and pollution data appear to come from the same day. It is unclear whether any lagging was implemented in the	No concern: Timing of baseline measurement reported and aligned with other measurements.	No concern: Timing of baseline measurement reported and aligned with other measurements.	Moderate concern: Timing of baseline measurement reported and aligned with other measurements. Although POPs were measured prior to diet onset and at the end, the change in POPs rather than absolute values is reported, which is concurrent with weight loss, making	Moderate concern: Timing of baseline measurement unclear but appear to align with other measurements. Although POPs were measured prior to diet onset and at the end, the change in POPs rather than absolute values is reported, which is concurrent with weight loss, making

	study analysis to account for downstream effects of pollutant exposure. Instead mean pollution over the whole period is used.			temporality impossible to establish.	temporality impossible to establish.
Low concern: Variation in exposure levels could have been insufficient to identify associations with mass loss parameters because the original study did not target those with a range of exposures. However, range of exposures in study population was substantial.	Moderate concern: Variation in exposure levels could have been insufficient to identify associations with mass loss parameters because the original study did not target those with a range of exposures. Although the annual levels of PMs exceeded current WHO targets, and showed up to 3.5 fold difference between highest and lowest values, all countries considered here are considerably lower in their PM levels compared to many LMICs especially in the global south. In study 2 the highest AQI values are 70, which only reaches the second lowest of 6 categories of concern for AQI (irnow.gov/aqi/aqi-basics/), which can	Moderate concern: Variation in exposure levels could have been insufficient to identify associations with mass loss parameters because the original study did not target those with a range of exposures.<br>BPA levels in the study participants were lower than in the general population and thus the ability to detect a trend if one were present was dramatically reduced. For the other chemical groups there was less concern that exposures were not representative and spanning an adequate range.	Moderate concern: Variation in exposure levels could have been insufficient to identify associations with mass loss parameters because the original study did not target those with a range of exposures. Range of exposures in study population was substantial for some (eg PFOS) but not all of the analytes, especially PFNA, making it difficult to detect true effects	Moderate concern: Variation in exposure levels could have been insufficient to identify associations with mass loss parameters because the original study did not target those with a range of exposures. the range of measurements in the chemicals was too small to see a biologically important difference between people, but this is hard to assess because the measure of variability used in the table reporting on POPs is not defined. The range of POPs measurements appears to be very small and largely lies within 10% of the	Low concern: Variation in exposure levels could have been insufficient to identify associations with mass loss parameters because the original study did not target those with a range of exposures. The range in POPs in the participants appears to be large enough to see a difference between those with high and low exposures if one were to exist.

exceed 301. Values here are thus very compressed compared the full range of possible values.

mean across all groups. Similarly, the mean change in most of the POPs is less than 10% over the 12 week diet, except for TCDF which increased by 23% in the vegetarian diet group. These issues may reduce the ability to detect a true biological effect.

5. Missing data	Low concern: At least 80% of participants completed the diet in the original study. Reasons for drop-out in the larger original cohort are provided in Sacks et al (2009): 811 people were recruited and 80% of them (645 people) completed the full two-year study. Provides post hoc analysis, in which only the weight loss part of the original study was included, and only those people who were followed throughout the weight loss phase and for whom samples existed for contaminant analysis were reported. Liu et al (2018) thus used the data from the 621 people of these who also had baseline plasma	Moderate concern: of the people who met inclusion criteria, all data appear to be reported, including 'null' results (people who gained weight or remained weight stable). Of interest would be comparisons with people who did not maintain use of the app and thus were not included. Characteristics of those who stopped using the App and those who completed the required 12 months were not reported. Although no data were missing, there was a great deal of potential flexibility in the allowed number of uploads above the minimum threshold. It is unclear how the authors accounted for differences between users in frequency of data input (eg people with the minimum 12 interactions and those who provided more data.	Low concern: At least 80% of participants completed the diet in the original study. Only the weight loss part of the original study was included. Full follow up and the full cohort is not included because only those that fulfilled data and sample availability criteria were included in this post hoc analysis, and as such there was 100% completion for the study participants considered, but drop out data and reasons are given in the earlier report from the study (Soenen et al 2012).	Low concern: At least 80% of participants completed the diet in the original study (Astrup et al 2015). Grandjean et al (2023) provides post hoc analysis, in which only those people who were followed throughout the weight loss and then weight maintenance phase and for whom samples existed for contaminant analysis were reported. Full follow up and the full cohort is not included because only those that fulfilled data and sample availability criteria were included in this post hoc analysis, and as such there was 100% completion for the study participants considered, but drop out data and reasons are given in the earlier report from the study.	Low concern: At least 80% of participants completed the diet in the original study. Full follow up and the full cohort is not included because only those that fulfilled data and sample availability criteria were included in this post hoc analysis, and as such there was 100% completion for the study participants considered, but drop out data and reasons are given in the earlier report from the study (Kahleova et al 2011).	Low concern: 100% completion reported

	samples available. Plasma samples were available from 592 people from the 6 month follow up and 460 from the 2 years follow up. Characteristics of those who dropped out of the study and those who completed the study, and between those with and without baseline plasma samples were comparable (Sacks et al 2009).	It is not clear if and how data were weighted for this possible difference in frequency. Information on number of logins is not reported				
6. Outcome measurement	No concern: Reported body mass, body composition or BMI measured in a clinical setting by a trained professional using standardised means of measurement that was clearly articulated.	High concern: mass measurements were self reported and thus not standardised. Even if advice were given on when and how to perform measurements, this is not reported and people do not typically have calibrated and high precision scales.	No concern: Reported body mass, body composition or BMI measured in a clinical setting by a trained professional using standardised means of measurement that was clearly articulated.	No concern: Reported body mass, body composition or BMI measured in a clinical setting by a trained professional using standardised means of measurement that was clearly articulated during the weight loss phase. Mass was self reported in weight maintenance phase	No concern: Reported body mass, body composition or BMI measured in a clinical setting by a trained professional using standardised means of measurement that was clearly articulated.	No concern: Reported body mass, body composition or BMI measured in a clinical setting by a trained professional using standardised means of measurement that was clearly articulated.

	No concern: Clinical professionals measuring outcomes were blinded to diet intervention	High concern: those measuring the outcome (BMI) were participants and thus with a vested interest in the measurement and success of the intervention	Low concern: Unclear if those measuring outcomes were blind to the diet intervention, but the outcome measurement preceded the measurement of the chemical exposure and could not influence the results reported	No concern: all participants on same diet so concealment was not needed	Low concern: Unclear if those measuring outcomes were blind to the diet intervention, but the outcome measurement preceded the measurement of the chemical exposure and could not influence the results reported	Low concern: Blinding of the outcome assessors to the participants' group is unreported. The impact of potential lack of blinding is unlikely to have affected the reported results since there were no treatment effects on weight loss or insulin levels.
7. Analysis and reporting	Moderate concern: The methods section was detailed enough to identify which statistical techniques were used and the methods and results section within the paper were internally consistent. However, reasons for and calculations of weighted means are not provided, and reasons for the multiple analysis done using both linear mixed effects modelling and stratified by different	Moderate concern: The methods section was detailed enough to identify which statistical techniques were used and the methods and results section within the paper were internally consistent but choice of covariates to use and the rationale for lack of activity and initial BMI input into study 2 is unclear.	Low concern: The methods section was detailed enough to identify which statistical techniques were used and the methods and results section within the paper were internally consistent.	Moderate concern: The methods section was detailed enough to identify which statistical techniques were used and the methods and results section within the paper were internally consistent, however the analysis of the low calorie, weight loss phase of the intervention is not specifically detailed. Reason for breaking the data into tertiles rather than using the continuous data available is not	Low concern: The methods section was detailed enough to identify which statistical techniques were used and the methods and results section within the paper were internally consistent.	Moderate concern: The methods section was detailed enough to identify which statistical techniques were used and the methods and results section within the paper were internally consistent. It is unclear whether the statistical techniques used to assess impact of chemical on mass loss or glycaemic control were appropriate because the use of partial correlations was not well described.

groups, in addition to quantile analysis is not clear. Reason for breaking the data into tertiles rather than using the continuous data available is not provided.			provided and the tertiles of weight loss are likely to be confounded by sex.		
Low concern: Power analysis was performed in Sacks et al (2009). The sample size was sufficiently large for the original study to detect a difference of 1.67 kg mass loss between groups over 2 years and assuming a 40% withdrawal rate.	High concern: power analysis was not performed but the sample size was so large that it is more likely a statistically significant but biologically unimportant effect was detected than lack of detection of a true effect. The small changes in BMI (~2) over time are likely to represent a range of 5-8kg change in absolute mass across a range of sizes from 150-200cm and from 50-200kg initial body mass. This represents a substantial variation in percentage mass reduction from 3 to more than 10% such that for some people this change	Low concern: No power analysis was performed but the number of participants across the treatment groups seems adequate to detect a true biological effect.	Low concern: Power analysis was performed for the original study on diets for weight maintenance but not for the low calorie weight loss phase. However, the number of participants is large enough to detect a true effect if one exists.	Low concern: No power analysis was performed but the number of participants across the treatment groups seems adequate to detect a true biological effect.	Moderate concern: No power analysis was reported such that eg lack of effect in women may be due to underpowered study. The change in POPs during fasting (and fat loss/ mass loss) was much greater in males (23-27% increase) compared to females (9-12.5% increase) which may explain why the association between the increase and plasma insulin was only seen in men and not women.

would be clinically significant, but only at lower initial BMIs. Initial BMI was not controlled for to allow for effect of pollutant burden on weight loss in those with equivalent BMI to be assessed. By combining all BMI categories, including normal weight people, the ability to detect clinically important differences may have been masked. The change in BMI with pollution level is very small. Figure 3 shows no change in BMI with pollution index given confidence intervals and is not accompanied with description or interpretation. Co-efficients referred to in text are not related to the Figure. The statistics reveal very strong evidence of a very weak association that is unlikely to be biologically meaningful and is several orders of magnitude

	smaller than effects such as gender and calorie intake.				
Low concern: A longer follow up after the weight loss diet intervention was performed in the original study, and this follow up period was included in analysis here.	Moderate concern: no follow up is reported but the overall duration of the diet could have varied between people, which may appear as less change per unit time in those who had a longer duration of recording in study 1 where timecourse was not considered.	Moderate concern: A longer follow up after the weight loss diet intervention was performed in the original study, and this follow up period was not included in analysis here, but reasons are not provided. More dramatic results in early phases of weight loss may be lost later and lack information on longer term pollutant effects is thus lacking.	Low concern A longer follow up after the weight loss diet intervention was performed in the original study, and this follow up period was included in analysis here, with reasons given for the lack of information on the 52 weeks time point, when too few participants remained in the study. More dramatic results in early phases of weight maintenance may be lost later and lack information on longer term pollutant effects is thus lacking.	Moderate concern: A longer follow up after the weight loss diet intervention was performed in the original study, and this follow up period was not included in analysis here, but reasons are not provided. More dramatic results in early phases of weight loss may be lost later and lack information on longer term pollutant effects is thus lacking	High concern: A longer follow up after the weight loss diet intervention was performed in the original study, and this follow up period was not included in analysis here, but reasons are not provided. For example, Chevrier et al (2000) has a second phase of the study and shares a large number of features with Imbeault et al (2002). More dramatic results in early phases of weight loss may be lost later and lack information on longer term pollutant effects is thus lacking.
Low concern: A study protocol was available but (understandably) did not include the post hoc contaminant	Moderate concern: A study protocol was not available to assess whether all the study's pre-specified primary and secondary outcomes	Moderate concern: A study protocol was available but (understandably) did not include the post hoc contaminant	Moderate concern: A study protocol was available but (understandably) did not include the post hoc contaminant	Moderate concern: A study protocol was not available to assess whether all the study's pre-specified primary and	High concern: A study protocol was not available to assess whether all the study's pre-specified primary and

measurements and analyses reported.	were reported but the methods and results sections tally in terms of analyses. It is unclear why mass as well as BMI was not included since these data were available to the authors. It is unclear whether data that were not available were not available because they were not recorded, not available because they were not released by the app provider or not requested.	measurements and analyses reported. The LOWER study also measured and reported mass, fasting blood glucose, waist-to -hip ratio, body composition/fat mass, circulating lipids, fasting insulin, HOMA-IR, blood pressure and hunger at baseline and 3 months later (Soenen et al 2012). It is unclear why only a subset of the metrics that were available were examined by van der Meer et al (2021).<br><br>In Soenen et al (2012) the overall sample size is smaller than that reported by van der Meer et al (2021), which means that additional participants were included in the study after the Soenen et al (2012) publication and for which drop out data are not available in van der Meer et al	measurements and analyses reported here. The DioGenes study also reported a number of glycaemia control and other adiposity metrics such as BMI, waist to hip ratio and body composition in previous papers (Goyenechea et al 2011) but only mass is reported here. It is thus possible that the effect is drawn from selected reporting of outcomes.<br><br>The lack of detailed reporting of the analysis of the weight loss phase of the study precludes evaluation or data extraction of the association between PFAS and weight loss.<br><br>Of the 400 of the original 932 participants with enough sample	secondary outcomes were reported. Some unplanned analysis was reported. Correlations were explored for glycaemic control, but not weight loss parameters and adiposity, despite measurement of these latter outcomes. It is thus possible that the effect is drawn from selected reporting of multiple analyses and these potential biases in reporting could lead to overestimate of the effect size.	secondary outcomes were reported. A large number of other papers with slightly different participant numbers all performed around the same time by the same group reporting on different outcomes (Doucet et al 1999; Chevrier et al 2000; Imbeault et al 2001; 2002b; Tremblay et al 2004; Pelletier et al 2000). We cannot eliminate the possibility that the most dramatic outcomes were selected for reporting, or that the participants were selected as a subgroup from a wider pool. Such reporting bias would lead to an overestimate of the effect size if a meta-analysis were performed. Many of the correlations

		(2021). It is thus not possible to assess from the publication if those with high exposures were more likely to drop out, or if those who dropped out because they struggled to lose weight were those with higher exposure or to establish whether other characteristics were similar.	available, 9 had incomplete covariate information and 10 had not lost enough weight to be included in the study reported here. PFAS in those 10 people is not reported and compared to those who successfully lost weight.		between glycaemic control metrics and POP changes were not strong or significant.
Low concern: uses STROBE checklist for reporting and Sacks et al (2009) from the original study provides a flow chart compliant with CONSORT guidelines	Moderate concern: no reference is made to STROBE reporting guidelines and several aspects on the checklist could be more comprehensive	Moderate concern: no reference to reporting guidelines, either STROBE (for posthoc analysis) or CONSORT for the original trial either here or in Soenen et al (2012) and several aspects on the checklist could be more comprehensive	Moderate concern: no reference to reporting guidelines, either STROBE (for posthoc analysis) or CONSORT for the original trial (Moore et al 2009) but a flow chart is provided in previous publications (Goyenechea et al 2011)	Moderate concern: no reference to reporting guidelines, either STROBE (for posthoc analysis) or CONSORT for the original trial but a flow chart is provided in previous publications (Kahleova et al 2011)	Moderate concern: study was performed before STROBE guidelines were issued in 2007and recruitment spanned the time of release of the original CONSORT guidelines

References:

Astrup A, Raben A, Geiker N. The role of higher protein diets in weight control and obesity-related comorbidities. *Int J. Obesity* 2015.39: 721–726. https://doi.org/10.1038/ijo.2014.216

Chevrier J, Dewailly E, Ayotte P, Mauriège P, Després, JP, Tremblay A. Body weight loss increases plasma and adipose tissue concentrations of potentially toxic pollutants in obese individuals. *Int J Obesity Related Metab Dis.* 2000. 24: 1272–1278. https://doi.org/10.1038/sj.ijo.0801380

Doucet E, St-Pierre S, Alméras N, Després JP, Bouchard C, Tremblay A. Evidence for the existence of adaptive thermogenesis during weight loss. *Brit J Nutr* 2001. 85: 715–723. https://doi.org/10.1079/bjn2001348

Goyenechea E, Holst C, van Baak MA et al. Effects of different protein content and glycaemic index of *ad libitum* diets on diabetes risk factors in overweight adults: the DIOGenes multicentre, randomized, dietary intervention trial. *Diabetes Metab Res Rev* 2011. 27:705–716. https://doi.org/10.1002/dmrr.1218

Imbeault P, Chevrier J, Dewailly E et al. Increase in plasma pollutant levels in response to weight loss in humans is related to *in vitro* subcutaneous adipocyte basal lipolysis. *Int J Obesity Related Metabolic Dis* 2001. 25: 1585–1591. https://doi.org/10.1038/sj.ijo.0801817.

Imbeault P, Tremblay A, Simoneau JA, Joanisse DR. Weight loss-induced rise in plasma pollutant is associated with reduced skeletal muscle oxidative capacity. *Am J Physiol. Endocrinol Metab.* 2002. 282: E574–E579. https://doi.org/10.1152/ajpendo.00394.2001

Kahleova H, Matoulek M, Malinska H. et al. Vegetarian diet improves insulin resistance and oxidative stress markers more than conventional diet in subjects with Type 2 diabetes. *Diabet Med.* 2011. 28: 549–559. https://doi.org/10.1111/j.1464-5491.2010.03209.x.

Larsen TM, Dalskov S, van Baak M et al. The Diet, Obesity and Genes (Diogenes) Dietary Study in eight European countries - a comprehensive design for long-term intervention. *Obesity Rev* 2010. 11: 76–91. https://doi.org/10.1111/j.1467-789X.2009.00603.x

Moore CS, Lindroos AK, Kreutzer M et al. Dietary strategy to manipulate *ad libitum* macronutrient intake, and glycaemic index, across eight European countries in the Diogenes Study. *Obesity Rev* 2010. 11: 67–75. https://doi.org/10.1111/j.1467-789X.2009.00602.x

Pelletier C, Doucet E, Imbeault P, Tremblay A. Associations between weight loss-induced changes in plasma organochlorine concentrations, serum T(3) concentration, and resting metabolic rate. 2002. *Toxicol Sci.* 67: 46–51. https://doi.org/10.1093/toxsci/67.1.46.

Sacks FM, Bray GA, Carey VJ et al. Comparison of weight-loss diets with different compositions of fat, protein, and carbohydrates. *NEJM.* 2009.360: 859–873. https://doi.org/10.1056/NEJMoa0804748

Schulz KF, Altman DG, Moher D, CONSORT Group, CONSORT 2010 statement: updated guidelines for reporting parallel group randomized trials. *Annal Intern Med.* 2010. 152: 726–732. https://doi.org/10.7326/0003-4819-152-11-201006010-00232

Soenen S, Bonomi AG, Lemmens SG,et al. Relatively high-protein or 'low-carb' energy-restricted diets for body weight loss and body weight maintenance? *Physiol Behav.* 2012. 107: 374–380. https://doi.org/10.1016/j.physbeh.2012.08.004

Tremblay A, Pelletier C, Doucet E, Imbeault P. Thermogenesis and weight loss in obese individuals: a primary association with organochlorine pollution. *Int J Obesity Related Metab Dis* 2004. 28: 936–939. https://doi.org/10.1038/sj.ijo.0802527

von Elm E, Altman DG, Egger M, Pocock SJ, Gøtzsche PC, Vandenbroucke JP. STROBE Initiative. The Strengthening the Reporting of Observational Studies in Epidemiology (STROBE) statement: guidelines for reporting observational studies. *J Clin Epidemiol.* 2008. 61:344-9. https://doi.org/10.1016/j.jclinepi.2007.11.008

Table S13: Signal questions, pre-consensus responses from the two evaluators (AS and KB) and consensus risk of bias evaluation for each of seven domains considered for each of the six papers evaluated.

Domain	Question	Paper	AS response	KB response	consensus
1: confounds	Were confounding factors identified?	Liu	yes	yes	
	Were strategies to deal with confounds stated?		yes	yes	
	What confounds, if any, did not appear to be accounted for?		Social economic status	NA	
	Did the participants receive similar treatment, other than the dietary intervention?		yes	yes	
	What is the predicted direction of bias due to unaccounted for confounds?		unclear	NA	
	reason for your answers		Social and economic factors may influence compliance and dropout rates	Very large number of confounds measured and accounted for: Covariates considered in multivariate adjustments included baseline age (continuous), sex, race, educational attainment (high school or less, some college, or college graduate or beyond), smoking status (never, former, or current smoker), alcohol consumption (continuous), physical activity (continuous). Moreover, menopausal status and hormone replacement therapy (women only) were also entered into the model in a sensitivity analysis	
	Overall assessment domain 1		Low	Low	Low

	Were confounding factors identified?	Ustulin	Partly	Partly	
	Were strategies to deal with confounds stated?		Partly	Partly	
	What confounds, if any, did not appear to be accounted for?		ethnicity; education; morbidities	Medications; activity levels; menopause, breastfeeding or pregnancy status; advice /plans given to users; number of logins; socioeconomic information	
	Did the participants receive similar treatment, other than the dietary intervention?		Unsure	No	
	What is the predicted direction of bias due to unaccounted for confounds?		Unsure	Unsure	
	reason for your answers		No pre-determined interventions. Participants were not allocated to groups, but data was from a phone app so self-reported. Environmental data should be OK but personal data is not verified.	The users were all users of an app that provides tailored lifestyle advice so meal plans, timing, other advice such as mindfulness and activity were not accounted for and no additional information on whether people were complying is given	
	Overall assessment domain 1				
	Were confounding factors identified?	Van der Meer	Partly	Partly	
	Were strategies to deal with confounds stated?		Partly	Partly	

	What confounds, if any, did not appear to be accounted for?		Ethnic group, socio-economic circumstances, co-morbidities (except Type 1 Diabetes), pre/post menopausal in female participants, lifestyle factors (alcohol consumption, smoking, exercise).	Smoking; alcohol consumption; socioeconomic group; normal diet or exercise; exercise regime during diet; ethnic background	
	Did the participants receive similar treatment, other than the dietary intervention?		yes	unclear	
	What is the predicted direction of bias due to unaccounted for confounds?		unclear	unclear	
	reason for your answers		Possible that unaccounted for medications or exercise could affect result? eg if those on particular drugs that influence metabolism of phthalates etc are also those that fail to lose weight, is it because of drugs or phthalates? If people must source food themselves, is the food choice/ source making it appear that those with higher phthalates (which could be those that choose precooked or processed	Sex, age and whether or not they had T2DM were included in the model, but other information collected was not (eg exercise levels). In Soenen et al it states that there were no heavy smokers or alcohol users. Where the amount of protein could not be achieved with the assigned menus, participants in high protein diets were given additional shakes, but in Soenen et al this does not appear to make a difference. People were given individual advice on diets where needed and it is not clear how that was recorded or reported. Direction of bias hard to estimate: may just lead to greater degree of noise in the data therefore harder to pull out a true effect	

			food for example) have slower weight loss?		
	Overall assessment domain 1		Moderate	Moderate	Moderat e
	Were confounding factors identified?	Grandjea n	Yes	Partly	
	Were strategies to deal with confounds stated?		Yes	Partly	
	What confounds, if any, did not appear to be accounted for?		Ethnicity and activity level have not been identified	socioeconomic status; menopause status; HRT; occupation or educational attainment; ethnicity; typical activity levels; normal diet or activity levels; activity levels during the LCD phase	
	Did the participants receive similar treatment, other than the dietary intervention?		No, but this is later weight maintenance phase when those in different countries received food through different mechanisms	Unsure	
	What is the predicted direction of bias due to unaccounted for confounds?		Unsure	Unsure	
	reason for your answers		Shops and instructions centres differed between the country that the people were in but they all received same during weight loss phase	They do account for whether the person comes from a single parent or both parent family, but this is listed as single parent if only one person is obese rather than if single parent. Since everyone was on one diet there is little that could be a confound, so accounting for covariates is more the issue. Clinical centre was included as covariate	
	Overall assessment domain 1		Low	Low	Low
	Were confounding factors identified?	Kahleova	Partly	Partly	
	Were strategies to deal with confounds stated?		Partly	No	

	What confounds, if any, did not appear to be accounted for?		Ethnic group; life style; comorbidities; medication	sex, age, duration or severity of diabetes status, concomitant medications, ethnic background, socioeconomic or educational attainment group	
	Did the participants receive similar treatment, other than the dietary intervention?		yes	yes	
	What is the predicted direction of bias due to unaccounted for confounds?		unclear	unclear	
	reason for your answers		More details are given in the original study	Statistics doesn't account for noise between participants and parameters that may correlate with higher exposure status	
	Overall assessment domain 1		Unsure	Moderate	Moderat e
	Were confounding factors identified?		Yes	Partly	
	Were strategies to deal with confounds stated?		partly	Partly	
	What confounds, if any, did not appear to be accounted for?		age; pregnancy; menopausal status.	socioeconomic group or other social inequality marker eg education level	
	Did the participants receive similar treatment, other than the dietary intervention?		No	No	
	What is the predicted direction of bias due to unaccounted for confounds?		Unclear	Unclear	

	reason for your answers		Fenfluramine or placebo groups are uneven and unexplained	Some participants were given an appetite suppressant. Both sex and fenfluoramine accounted for in analysis. All 'Caucasian'. Many other potential confounds are excluded.	
	Overall assessment domain 1		Low	Moderate	Low
2. Participant selection	Were inclusion criteria such as sex, age and demographic/ source population clearly stated?	Liu	yes	yes	
	What inclusion criteria were missing, if any?		NA	NA	
	Were specific medications excluded?		no	yes	
	Were specific health conditions excluded?		yes	yes	
	Were specific life style factors excluded?		no	yes	
	Indicate the exclusions identified		Abnormal thyroid activity	40% would be men. Participants had to be 30 to 70 years of age and have a BMI of 25 to 40. Major criteria for exclusion were the presence of diabetes or unstable cardiovascular disease, the use of medications that affect body weight, and insufficient motivation as assessed by interview and questionnaire. It is not clear which medications were excluded and what counts as insufficient motivation. This detail is given in sacks et al 2009. People with type 2 diabetes controlled with diet, or with hypertension or hyperlipidaemia treated with diet or drugs, were eligible to participate. Exclusions were diabetes treated with oral medications or insulin, serious gastrointestinal disease, alcohol or drug abuse, treatment for an eating disorder, unstable or recent onset of cardiovascular disease, or other serious illness; weight-loss medications and other drugs that affect body weight such as some anti-psychotic or	

				anti-depressant drugs, or corticosteroids; hypothyroidism defined by abnormal thyroid stimulating hormone (TSH); urinary microalbumin >100 ug/g creatinine; or unstable dose of medication for hyperlipidemia, hypertension, or psychiatric disorder all given in Sacks et al 2009.	
	Are other relevant aspects of the participants' individual characteristics reported t hat may influence their weight loss trajectory during the diet?		yes	yes	
	List any characteristics that were provided or were missing		PROVIDED - age; race; BMI; Weight; Waist circumference; RMR; Education Level; Smoking Status; Alcohol consumption; Physical Activity; Blood pressure; Blood Glucose ; Insulin; Cholesterol; LDL; HDL; Triglycerides; FreeT3; Free T4; Serum PFOS; PFOA; PFHxS; PFNA; PFDA MISSING: medications, social economic/occupation; diabetic status although blood glucose is reported	Using standardized questionnaires, authors obtained information on age, sex, race (white, black, Hispanic, or other), educational attainment (high school or less, some college, or college graduate or beyond), smoking status (never, former, or current smoker), alcohol consumption (drinks/week), menopausal status (yes or no), and hormone replacement therapy use (yes or no). Physical activity was assessed using a questionnaire, which included 16 items inquiring about levels of habitual physical activities. All participants tested for normal thyroid function at study baseline	
	If overweight or obesity was included/ excluded, are the criteria used to define the condition provided?		Yes	Yes	

	Were participants recruited from a particular demographic or setting eg weight loss clinic, general population, high exposure population?		Yes	Yes	
	If yes, what setting or demographic did the study recruit from?		mass mailing described in reference 37	Mass mailings: names were identified from lists of registered voters or drivers. The primary sources of mailing lists were commercial vendors and local governments (for lists of registered voters or drivers). Secondary methods included advertisements on buses and subways, worksite advertisements, newspaper advertisements, distribution of recruitment flyers, and mailings to local healthcare centres and businesses (from Sacks et al 2009 supp mat)	
	Are the demographics of the participants, such as age, sex, socioeconomic group, ethnic background and their relevant clinical characteristics reported?		Partly	Yes	
	Is the location (city and country) reported?		Yes	Yes	
	Is the year(s) of recruitment to the study reported or easily available (for example in a clinical trial registration)?		Yes	Yes	
	If participants were diabetic, is the duration of diabetes reported?		No	No	

	Were participants weight stable and in steady state at start of the diet intervention?		unreported	unreported	
	If diabetes or IGT was excluded/ included, are the criteria for diabetes diagnosis provided?		no	unclear	
	Is complete and consecutive inclusion used?		unclear	unclear	
	For randomised controlled trials only: was true randomization used for assignment of participants to treatment groups?		unreported	Yes (see Sacks et al (2009))	
	Where a 'control' group used, is the diet clearly specified and matched in some way with the weight reduction diet and or lifestyle changes requested in control group clearly specified?		N/A	yes	
	What is the predicted direction of bias due to the information available about participants and their characteristics?		unclear	No effect	
	Please provide any additional information		Additional detail in reference 37	The randomisation is unclear: from Sacks et al 2009, the computer ensured even mix between people but maybe that was done through algorithm rather than random allocation. Sacks et al 2009 supp mat	

	that shows the reason for your answers			says: 'Randomization assignments to one of 4 diet groups were generated by the data manager at the coordinating centre, upon request of a study dietitian, after confirming, by computer program, that all screening activities had occurred, that the participant met all eligibility criteria, and that all required baseline data had been collected. Diet group assignments were stratified by site with varying block sizes to ensure a balance at each site. ' Randomisation to diet should not matter here because the diet didn't affect weight loss rate and there can be no effect of randomisation on exposure. It is unclear whether diabetes status is included.	
	Overall assessment of bias due to participant selection		Low	Low	Low
	Were inclusion criteria such as sex, age and demographic/ source population clearly stated?	Ustulin	Yes	Partly	
	What inclusion criteria were missing, if any?		ethnicity; education; morbidities	whether patients were diabetic or had any other disease; no information on medical history	
	Were specific medications excluded?		No	No	
	Were specific health conditions excluded?		No	No	
	Were specific life style factors excluded?		No	No	
	Indicate the exclusions identified			none. only the use of the app was used as criteria for selection so people who did not input core details or whose age was 42 were excluded plus any that did not use the app for a year, logged in less than one month at a time and provided inadequate detail on foods eaten	

	Are other relevant aspects of the participants' individual characteristics reported t hat may influence their weight loss trajectory during the diet?		No (and more detail would be informative)	No (and more detail would be informative)	
	List any characteristics that were provided or were missing		missing - ethnicity; education; morbidities	no detail on activity or on the usual diet or diet and advice provided	
	If overweight or obesity was included/ excluded, are the criteria used to define the condition provided?		Yes	NA	
	Were participants recruited from a particular demographic or setting eg weight loss clinic, general population, high exposure population?		Yes	Yes	
	If yes, what setting or demographic did the study recruit from?		Users of an app	Noom coach users	
	Are the demographics of the participants, such as age, sex, socioeconomic group, ethnic background and their relevant clinical characteristics reported?		Partly	Partly	

	Is the location (city and country) reported?		Yes	Yes	
	Is the year(s) of recruitment to the study reported or easily available (for example in a clinical trial registration)?		Yes	Yes	
	If participants were diabetic, is the duration of diabetes reported?		No	NA	
	Were participants weight stable and in steady state at start of the diet intervention?		Unreported	Unreported	
	If diabetes or IGT was excluded/ included, are the criteria for diabetes diagnosis provided?		NA	NA	
	Is complete and consecutive inclusion used?		Yes	Unclear	
	For randomised controlled trials only: was true randomization used for assignment of participants to treatment groups?		NA	NA	
	Where a 'control' group used, is the diet clearly specified and matched in some way with the weight reduction diet		NA	NA	

	and or lifestyle changes requested in control group clearly specified?				
	What is the predicted direction of bias due to the information available about participants and their characteristics?		Unsure	Unsure	
	Please provide any additional information that shows the reason for your answers		Overall, participants are not sufficiently characterised.	Very sparse detail on users and this is likely to lead to overall greater variability in the data and the ability to detect an effect if one is present. People tend to be young in this study and are also self-motivated to lose weight. may bias towards groups with easy phone access and those who are comfortable using such technology	
	Overall assessment of bias due to participant selection		Moderate	Moderate	Moderat e
	Were inclusion criteria such as sex, age and demographic/ source population clearly stated?	Van der Meer	Partly	Yes	
	What inclusion criteria were missing, if any?		Ethnic group; menopausal status in females; co-morbidities; medication	N/A	
	Were specific medications excluded?		No	No	
	Were specific health conditions excluded?		Yes	Yes	
	Were specific life style factors excluded?		No	No	
	Indicate the exclusions identified		Type 1 diabetes excluded (and type 2 is mentioned in	Type 1 diabetes. In Soenen et al study also excluded cancer, HIV, psychiatric disease, more than 10% weight loss in previous 6 mo; pregnancy and breastfeeding	

			table 1 but not as an exclusion)		
	Are other relevant aspects of the participants’ individual characteristics reported t hat may influence their weight loss trajectory during the diet?		Yes	No (and more detail would be informative)	
	List any characteristics that were provided or were missing		Type 2 diabetes is listed in table 1. Other info to consider could be socio-economic status, occupation (could influence exposure e.g. manufacturing industry workers versus office or outdoor working) More detail is given in original study paper e.g. 'Exclusion criteria were cancer, HIV infection, psychiatric disease, more than 10% reduction of BW during the last six months; moreover, women who were pregnant or breastfeeding were excluded'. (Soenen et al 2012)	Exercise regularity; concurrent medications especially for diabetes management are missing	
	If overweight or obesity was included/ excluded, are the criteria used to define the condition provided?		yes	yes	

	Were participants recruited from a particular demographic or setting eg weight loss clinic, general population, high exposure population?		yes	yes	
	If yes, what setting or demographic did the study recruit from?		Obese Dutch individuals referred by their general practitioner or specialist to a weight management program of an outpatient-clinic in Hengelo, The Netherlands. Note the number of participants is different to those reported by (Soenen et al 2012)	potential clients of a weight management program of an outpatient-clinic in the city of Hengelo, The Netherlands, referred by GP or specialist	
	Are the demographics of the participants, such as age, sex, socioeconomic group, ethnic background and their relevant clinical characteristics reported?		Partly	partly	
	Is the location (city and country) reported?		Country only	yes	
	Is the year(s) of recruitment to the study reported or easily available (for example in a clinical trial registration)?		yes	Yes	

	If participants were diabetic, is the duration of diabetes reported?		No	no	
	Were participants weight stable and in steady state at start of the diet intervention?		yes	Yes	
	If diabetes or IGT was excluded/ included, are the criteria for diabetes diagnosis provided?		no	no	
	Is complete and consecutive inclusion used?		no	unclear	
	For randomised controlled trials only: was true randomization used for assignment of participants to treatment groups?		Unreported	No	
	Where a 'control' group used, is the diet clearly specified and matched in some way with the weight reduction diet and or lifestyle changes requested in control group clearly specified?		N/A	yes	
	What is the predicted direction of bias due to the information available about participants and their characteristics?		unclear	unclear	

	Please provide any additional information that shows the reason for your answers		More detail in Soenen et al 2012		
	Overall assessment of bias due to participant selection		Low	Moderate	Low
	Were inclusion criteria such as sex, age and demographic/ source population clearly stated?	Grandjea n	Partly	Yes	
	What inclusion criteria were missing, if any?		referred to ref 19 which then cites previous papers	NA -provided in detail in Goyenechea et al (2011)	
	Were specific medications excluded?		Unsure	Yes	
	Were specific health conditions excluded?		Unsure	Yes	
	Were specific lifestyle factors excluded?		Unsure	Yes	
	Indicate the exclusions identified			A range of medications and diseases that might affect weight loss or regain were excluded and this is extensive albeit reported in previous study. Note that to be included in the weight regain study, people had to have lost 8% or more of their original body mass so people who had low weight loss would not appear here. Drug use and alcohol use were excluded but activity levels were not. Specific diets and those that had recently lost weight of more than 3kg were excluded. This information is in previous papers by the group. Pregnancy, lactation, and intention to become pregnant is excluded but no mention of menopause so we may have women who are on HRT or peri/ post-menopausal	
	Are other relevant aspects of the		No (and more detail would be informative)	No (but sufficient detail is given in other cited papers)	

	participants' individual characteristics reported t hat may influence their weight loss trajectory during the diet?				
	List any characteristics that were provided or were missing		ethnicity; activity level	economic group; income; exercise	
	If overweight or obesity was included/ excluded, are the criteria used to define the condition provided?		Unsure	Yes	
	Were participants recruited from a particular demographic or setting eg weight loss clinic, general population, high exposure population?		No	yes	
	If yes, what setting or demographic did the study recruit from?			weight loss program waiting lists, patients referred from practice but also includes general public. The proportion of these does not seem to be reported	
	Are the demographics of the participants, such as age, sex, socioeconomic group, ethnic background and their relevant clinical characteristics reported?		Partly	Partly	
	Is the location (city and country) reported?		Yes	Yes	

	Is the year(s) of recruitment to the study reported or easily available (for example in a clinical trial registration)?		Yes	Yes	
	If participants were diabetic, is the duration of diabetes reported?		No	No	
	Were participants weight stable and in steady state at start of the diet intervention?		Unreported	Yes – reported in cited paper	
	If diabetes or IGT was excluded/ included, are the criteria for diabetes diagnosis provided?		NA	Unsure	
	Is complete and consecutive inclusion used?		Unclear	No	
	For randomised controlled trials only: was true randomization used for assignment of participants to treatment groups?		Unreported	Unreported but not relevant	
	Where a 'control' group used, is the diet clearly specified and matched in some way with the weight reduction diet and or lifestyle changes		Yes	NA	

	requested in control group clearly specified?				
	What is the predicted direction of bias due to the information available about participants and their characteristics?		Unsure	Unsure	
	Please provide any additional information that shows the reason for your answers		Details not provided in Grandjean but in paper cited by ref 19	everyone is on one diet so no randomisation; preselects for those who have lost weight effectively so likely to underestimate effect	
	Overall assessment of bias due to participant selection		Unsure	Moderate	Moderat e
	Were inclusion criteria such as sex, age and demographic/ source population clearly stated?	Kahleova	partly	yes	
	What inclusion criteria were missing, if any?		Ethnic group, lifestyle factors, socio economic status	NA	
	Were specific medications excluded?		No	Yes	
	Were specific health conditions excluded?		No	Yes	
	Were specific lifestyle factors excluded?		yes	yes	
	Indicate the exclusions identified		use of insulin, abuse of alcohol or drugs, pregnancy, lactation, or current use of a vegetarian diet. excluded -	Information from Kahleova et al 2011: Inclusion criteria were: Type 2 diabetes, age 30–70 years, HbA1c between 6 and 11% (42–97 mmol µ mol), BMI between 25 and 53, and willingness to change dietary habits and follow a prescribed exercise programme. Exclusion criteria were HbA1c < 6% (< 42 mmol µ mol) or > 11% (> 97 mmol µmol), use	

		mentioned in original study (ref 15)	of insulin, abuse of alcohol or drugs, pregnancy, lactation, or current use of a vegetarian diet.	
Are other relevant aspects of the participants' individual characteristics reported t hat may influence their weight loss trajectory during the diet?		yes	No (and more detail would be informative)	
List any characteristics that were provided or were missing		Provided: willingness to change dietary habits; willingness to follow a prescribed exercise programme	Not provided: anti-diabetic or other medications; pre-existing diabetes duration	
If overweight or obesity was included/ excluded, are the criteria used to define the condition provided?		Yes	Yes	
Were participants recruited from a particular demographic or setting eg weight loss clinic, general population, high exposure population?		Not reported	yes	
If yes, what setting or demographic did the study recruit from?		NA	Subjects with Type 2 diabetes treated by oral hypoglycaemic agents were recruited from February to May 2008. Referred by endocrinologist. In Kahleova et al 2011	
Are the demographics of the participants, such as age, sex, socioeconomic group, ethnic		Partly	Party	

	background and their relevant clinical characteristics reported?				
	Is the location (city and country) reported?		No	Yes	
	Is the year(s) of recruitment to the study reported or easily available (for example in a clinical trial registration)?		Yes	Yes	
	If participants were diabetic, is the duration of diabetes reported?		No	No	
	Were participants weight stable and in steady state at start of the diet intervention?		Unreported	unreported	
	If diabetes or IGT was excluded/ included, are the criteria for diabetes diagnosis provided?		Yes	yes	
	Is complete and consecutive inclusion used?		yes	unreported	
	For randomised controlled trials only: was true randomization used for assignment of participants to treatment groups?		yes	unreported	
	Where a 'control' group used, is the diet clearly		yes	yes	

	specified and matched in some way with the weight reduction diet and or lifestyle changes requested in control group clearly specified?				
	What is the predicted direction of bias due to the information available about participants and their characteristics?		unclear	unclear	
	Please provide any additional information that shows the reason for your answers		Matched to a conventional diet rather than a 'no change control'	Note that it is quite possible the people would know if they were eating a vegetarian or non-vegetarian diet so were not blinded to treatment. Randomisation not reported but given lack of difference between diets and lack of knowledge about exposure status this will have little effect on results here. It's not clear if people were already trying to lose weight at start and thus what their levels of POPs were. if the ones who had higher plasma levels at start were already trying to lose weight before the study began, they may have had a. higher levels of reported POPs and b. less pronounced weight loss rates/ less improvement in glycaemia because they were already in trajectory of improving glycaemia and losing weight It is possible that this could affect results here because we don't know starting weight stability	
	Overall assessment of bias due to participant selection		Low	Moderate	Low
	Were inclusion criteria such as sex, age and demographic/ source population clearly stated?	Imbeault	partly	yes	

	What inclusion criteria were missing, if any?		age	NA	
	Were specific medications excluded?		Yes	Yes	
	Were specific health conditions excluded?		Yes	Yes	
	Were specific lifestyle factors excluded?		Yes	Yes	
	Indicate the exclusions identified		medication, which could have influenced triglyceride metabolism – β- blockers, antihypertensive drugs, etc;	Details given on exclusion are quite detailed: Subjects with cardiovascular disease, diabetes mellitus, endocrine disorders, or those on medication, which could have influenced triglyceride metabolism (beta-blockers, antihypertensive drugs, etc), were excluded. All participants undertook fewer than 2 exercise sessions of 30 minutes/week), nonsmokers, and moderate alcohol consumers (i.e., fewer than 140 g/week). None had recently been on a diet or involved in a weight-reducing program, and their body weight had been stable during the last 6 months prior to the study.	
	Are other relevant aspects of the participants' individual characteristics reported t hat may influence their weight loss trajectory during the diet?		No (and more detail would be informative)	No (and more detail would be informative)	
	List any characteristics that were provided or were missing		Provided: medical history; activity level; certain medications; lifestyle factor (smoking and alcohol) were all considered	Missing: Women's menopausal/ HRT status. Assume pregnancy and breast feeding were excluded but this is not clear	
	If overweight or obesity was included/ excluded, are the criteria used to		Yes	Yes	

	define the condition provided?				
	Were participants recruited from a particular demographic or setting eg weight loss clinic, general population, high exposure population?		yes	yes	
	If yes, what setting or demographic did the study recruit from?		Caucasians from general population recruited through the media	They were recruited through the media so assumed to be general public	
	Are the demographics of the participants, such as age, sex, socioeconomic group, ethnic background, and their relevant clinical characteristics reported?		Partly	Partly	
	Is the location (city and country) reported?		No	Yes	
	Is the year(s) of recruitment to the study reported or easily available (for example in a clinical trial registration)?		No	Yes	
	If participants were diabetic, is the duration of diabetes reported?		No	No	
	Were participants weight stable and in steady state		Yes	Yes	

	at start of the diet intervention?				
	If diabetes or IGT was excluded/ included, are the criteria for diabetes diagnosis provided?		unclear	no	
	Is complete and consecutive inclusion used?		no	unreported	
	For randomised controlled trials only: was true randomization used for assignment of participants to treatment groups?		no	unreported	
	Where a 'control' group used, is the diet clearly specified and matched in some way with the weight reduction diet and or lifestyle changes requested in control group clearly specified?		partly	NA	
	What is the predicted direction of bias due to the information available about participants and their characteristics?		unclear	unclear	
	Please provide any additional information that shows the reason for your answers		Participants all on 15-week diet but some had fenfluramine and others a placebo	Information quite sparse. Criteria for diabetes evaluation for exclusion not made clear	

	Overall assessment of bias due to participant selection		Low	Low	Low
3. Diet intervention	Is the diet duration provided?	Liu	Yes	Yes	
	Is the nature of the diet described in sufficient detail, such as total calories and composition by macronutrient group/ specific food stuffs and timing of meals?		Partly	Yes	
	What was the setting in which meals were eaten?		Participants given dietary advice but prepared food unsupervised	Participants given dietary advice but prepared food unsupervised	
	How was compliance/ adherence to diet ensured?		check-up/regular conversations with nutritionist	Diet diaries	
	Is there a possibility that participants did not adhere to the diet?		Yes	Yes	
	Is the diet duration adequate to allow for sufficient weight loss to occur or glycaemic control be established?		Yes	Yes	
	Were participants blind to treatment assignment?		Yes	Yes	
	Were parameters for 'completion' of the diet programme clearly reported?		Partly	No	

	What is the predicted direction of bias due to the identified potential for departures from the intended diet?		Leads to overestimate of apparent effect	Unclear	
	Reason for your answers		Non-compliance more likely to be over rather than under eating on diet --> more rapid weight gain more likely. More details about the study provided in ref 37	I cannot find any details on how many sessions a participant had to attend or how many diaries etc had to be submitted for them to be considered complete. Liu et al only reports on the people who were successfully followed up over 24 months from the total study. Sacks et al reports: 'Group sessions were held once a week, 3 of every 4 weeks during the first 6 months and 2 of every 4 weeks from 6 months to 2 years; individual sessions were held every 8 weeks for the entire 2 years. Daily meal plans in 2-week blocks were provided. Participants were instructed to record their food and beverage intake in a daily food diary and in a Web-based self-monitoring tool that provided information on how closely their daily food intake met the goals for macronutrients and energy. Behavioural counselling was integrated into the group and individual sessions to promote adherence to the assigned diets. Contact among the groups was avoided.' However, the paper doesn't tell us how this information was used.	
	Overall assessment of bias due to potential deviations from intended diet		Moderate	Low	Low
	Is the diet duration provided?	Ustulin	Unclear	Unclear	
	Is the nature of the diet described in sufficient detail, such as total calories and composition by macronutrient group/		Partly	No	

	specific food stuffs and timing of meals?				
	What was the setting in which meals were eaten?		Other	Unreported	
	How was compliance/ adherence to diet ensured?		Undescribed	Undescribed	
	Is there a possibility that participants did not adhere to the diet?		Yes	Yes	
	Is the diet duration adequate to allow for sufficient weight loss to occur or glycaemic control be established?		Unclear	Yes	
	Were participants blind to treatment assignment?		No	No	
	Were parameters for 'completion' of the diet programme clearly reported?		No	Partly	
	What is the predicted direction of bias due to the identified potential for departures from the intended diet?		Unsure	Unsure	
	Reason for your answers		No specific intervention. Assumption that app users were sufficiently aware of the importance of diet, but they were not aware that	If people who do not stick to the advice are those who experience higher levels of pollution they will not be seen in this study - tend to lose people - no tests of whether those that dropped out were similar to those that didn't in terms of pollution exposure is not tested. No information is given on what participants were advised to do or how well they stuck to that advice is reported. they do have a	

			their data was in the study and data was self - reported	minimum requirement for log ins and reports for food so average calorie intake is estimated but this can vary between participants, so the estimate is not only based on self-reports but isn't consistent between people	
	Overall assessment of bias due to potential deviations from intended diet				
	Is the diet duration provided?	Van der Meer	yes	Yes	
	Is the nature of the diet described in sufficient detail, such as total calories and composition by macronutrient group/ specific food stuffs and timing of meals?		partly	partly	
	What was the setting in which meals were eaten?		unreported	Participants given dietary advice but prepared food unsupervised	
	How was compliance/ adherence to diet ensured?		unreported	check-up/regular conversations with nutritionist	
	Is there a possibility that participants did not adhere to the diet?		Yes	Yes	
	Is the diet duration adequate to allow for sufficient weight loss to occur or glycaemic control be established?		Yes	Yes	
	Were participants blind to<br>treatment assignment?		Unreported	Unreported	

	Were parameters for 'completion' of the diet programme clearly reported?		unclear	partly	
	What is the predicted direction of bias due to the identified potential for departures from the intended diet?		Leads to underestimate of apparent effect	Unclear	
	Reason for your answers			Total diet composition is provided in Soenen et al 2012, with some information on what food stuffs were included, although more detail on specific menus is not. Timings of meals is not provided. Appears that people were given food menus but not the food itself. Hard to see how participants could have been blinded because they were given detailed macronutrient advice so seems likely they knew what they were trying to increase or decrease in the diet. Weekly clinic assessments made in first month, every 2 weeks in next 3 months and monthly during last eight months. Unclear if this is the same as group sessions to support the diet and healthy eating. So efforts made to ensure compliance but not reported what people had to attend to remain in programme. Soenen et al 2012 reports dropouts and these are people who could not attend the clinics but it is not clear how many were tolerated. In van der Meer et al we know that they only selected people for the post hoc analysis who had urine available at start and end of the 3 months, but we don't know how much the people did or did not attend clinics between. Protein content of diet also monitored through N in the urine, but this is more about protein and not weight loss.	
	Overall assessment of bias due to potential deviations from intended diet			Moderate	Moderat e

	Is the diet duration provided?	Grandjean	Yes	yes	
	Is the nature of the diet described in sufficient detail, such as total calories and composition by macronutrient group/ specific food stuffs and timing of meals?		No	Partly	
	What was the setting in which meals were eaten?		Unreported	Unreported	
	How was compliance/ adherence to diet ensured?		Unreported	Unreported	
	Is there a possibility that participants did not adhere to the diet?		Yes	Unclear	
	Is the diet duration adequate to allow for sufficient weight loss to occur or glycaemic control be established?		Yes	Yes	
	Were participants blind to treatment assignment?		No	No	
	Were parameters for 'completion' of the diet programme clearly reported?		Unsure	Yes	
	What is the predicted direction of bias due to the identified potential		Leads to overestimate of apparent effect	Leads to underestimate of apparent effect	

	for departures from the intended diet?				
	Reason for your answers		Diet not described in detail by Grandjean but in https://doi.org/10.1111/j.1467-789X.2009.00602.x	Only those people who stuck to the diet are reported because this study reports mostly on the follow up period so for the LCD element we are only seeing the data from the people who already lost the 8% or more body mass. This shows that they were adhering to the diet adequately, but we have no information about those (10 people for which samples were available) who did not adhere during the LCD phase. The 8 weeks is clearly long enough for very substantial weight loss in some people because it is an extreme restriction. People given food sachets but not clear on meal timings and settings in which meals were eaten or how their compliance was monitored during the LCD phase because most attention was on follow up	
	Overall assessment of bias due to potential deviations from intended diet		Moderate	Moderate	Moderate
	Is the diet duration provided?	Kahleova	Yes	Yes	
	Is the nature of the diet described in sufficient detail, such as total calories and composition by macronutrient group/ specific food stuffs and timing of meals?		Yes	partly	
	What was the setting in which meals were eaten?		Participants given meal packs to eat unsupervised	meals given in a restaurant and pick up is monitored	
	How was compliance/ adherence to diet ensured?		check-up/regular conversations with nutritionist	Diet diaries	

	Is there a possibility that participants did not adhere to the diet?		Yes	Yes	
	Is the diet duration adequate to allow for sufficient weight loss to occur or glycaemic control be established?		Yes	Yes	
	Were participants blind to treatment assignment?		No	No	
	Were parameters for 'completion' of the diet programme clearly reported?		Unclear	yes	
	What is the predicted direction of bias due to the identified potential for departures from the intended diet?		unclear	No overall effect	
	Reason for your answers		More detail provided in ref 15	While it may be possible to cheat on the diet, several measures in place to monitor compliance and reading Kahleova et al 2011 they also assigned people to different adherence categories. The adherence then appears to have been used in Kahleova et al 2015. Adherence % is reported at more than 70%	
	Overall assessment of bias due to potential deviations from intended diet		Moderate	Low	Low
	Is the diet duration provided?	Imbeault	Yes	Yes	
	Is the nature of the diet described in sufficient		Partly	No	

	detail, such as total calories and composition by macronutrient group/ specific food stuffs and timing of meals?				
	What was the setting in which meals were eaten?		Unreported	Unreported	
	How was compliance/ adherence to diet ensured?		unreported	unreported	
	Is there a possibility that participants did not adhere to the diet?		Yes	Yes	
	Is the diet duration adequate to allow for sufficient weight loss to occur or glycaemic control be established?		Yes	Yes	
	Were participants blind to treatment assignment?		unreported	No	
	Were parameters for 'completion' of the diet programme clearly reported?		Unclear	No	
	What is the predicted direction of bias due to the identified potential for departures from the intended diet?		unclear	unclear	
	Reason for your answers			All people on same diet so blinding is not an issue here. Not clear about blinding with regard to placebo versus appetite suppressant. No information provided on adherence to diet in terms of how it was	

				monitored and how much people had to comply to remain in the trial. Doucet et al 1999, which contains more detail on the regime used here, includes information on dietitian visits but regularity and how these were monitored is unclear	
	Overall assessment of bias due to potential deviations from intended diet		Moderate		
4. contaminant measurement	How is the exposure measured in the individual ?	Liu	using a direct method from a sample taken from the person	using a direct method from a sample taken from the person	
	Is the exposure measured in an appropriate and validated matrix?		Yes	Yes	
	Is the exposure measurement from a single spot sample or more than one measurement (eg 24 h urine)?		single point sample	single point sample	
	Is it appropriate for exposure to be estimated from the sampling regime use?		Yes	Yes	
	Is the exposure measured in way that is either the gold standard for the chemical or another well accepted way?		Yes	Yes	
	Is a single chemical measured or are multiple		Several chemicals measured and reported individually	Several chemicals measured and reported individually	

	congeners/ metabolites within a given group measured?				
	Is there a concern that the variation in exposure levels was insufficient to identify associations with mass loss rate or glycaemic control?		yes	unclear	
	Was information on exposure status recorded prior to the diet intervention?		Yes	Yes	
	Is it clear exactly when baseline and endpoint exposure data was collected relative to start and end of the dietary intervention?		Yes	Yes	
	Was the timing of baseline and endpoint exposure measures relative to the start and end of the diet consistent between participants?		Yes	Yes	
	Could measurement of exposure status have been affected by knowledge of the outcome (mass change)?		no	no	

	What is the predicted direction of bias due to exposure measurement?		unclear	No overall effect	
	reason for your answers		Could be either over/under estimate	Reports on how reproducible within person blood measures of PFAS are	
	Overall assessment of bias due to exposure measurement		Low	Low	Low
	How is the exposure measured in the individual ?	Ustulin	environmental monitoring in place of work or at home	modelling/ risk approach such as assuming exposure based on residency in an area or occupation	
	Is the exposure measured in an appropriate and validated matrix?		Yes	Unsure	
	Is the exposure measurement from a single spot sample or more than one measurement (eg 24 h urine)?		other (eg average value over week/ month)	other (eg average value over week/ month)	
	Is it appropriate for exposure to be estimated from the sampling regime use?		Unsure	Yes but with low precision	
	Is the exposure measured in way that is either the gold standard for the chemical or another well accepted way?		Yes	No	
	Is a single chemical measured or are multiple		Several chemicals measured and reported individually	Several chemicals within a group measured and reported as a sum and individually	

	congeners/ metabolites within a given group measured?				
	Is there a concern that the variation in exposure levels was insufficient to identify associations with mass loss rate or glycaemic control?		Unsure	Unsure	
	Was information on exposure status recorded prior to the diet intervention?		No	Unsure	
	Is it clear exactly when baseline and endpoint exposure data was collected relative to start and end of the dietary intervention?		Partly	No	
	Was the timing of baseline and endpoint exposure measures relative to the start and end of the diet consistent between participants?		Yes	No	
	Could measurement of exposure status have been affected by knowledge of the outcome (mass change)?		No	Unsure	

	What is the predicted direction of bias due to exposure measurement?		Unsure	Unsure	
	reason for your answers		Additional individual exposure e.g. due to occupational environment, not taken into account	The information on how data were obtained and matched to participants is not very detailed so it's not clear how the information was integrated with when people were losing weight.<br><br>The average for PM2.5 is above the current AQG level of 5 ug/m3 from WHO (https://apps.who.int/iris/bitstream/handle/10665/345329/9789240034228-eng.pdf) which is when they think levels start to have an effect on all cause mortality for all people across cities reported in Table 2. The highest levels are in Seoul at 24 but current levels in other cities are much higher - 46-47 in places like Cairo and Mumbai and can go over 80 so the range reflects lower levels in wealthier countries (see https://www.visualcapitalist.com/mapped-air-pollution-levels-around-the-world-2022/) and levels were previously higher (see 2018 data)<br><br>For PM10 the target level is 15 from WHO but can be over 150. The highest here was 46 so 3.5 fold spread but not as high as in global south and LMICs	
	Overall assessment of bias due to exposure measurement		Moderate		
	How is the exposure measured in the individual ?	Van der Meer	using a direct method from a sample taken from the person	using a direct method from a sample taken from the person	
	Is the exposure measured in an appropriate and validated matrix?		Yes	Yes	

	Is the exposure measurement from a single spot sample or more than one measurement (eg 24 h urine)?		24 h exposure	24 h exposure	
	Is it appropriate for exposure to be estimated from the sampling regime use?		Yes	Yes	
	Is the exposure measured in way that is either the gold standard for the chemical or another well accepted way?		Unsure	Yes	
	Is a single chemical measured or are multiple congeners/ metabolites within a given group measured?		Several chemicals measured and reported individually	Several chemicals measured and reported individually	
	Is there a concern that the variation in exposure levels was insufficient to identify associations with mass loss rate or glycaemic control?		no	Unclear	
	Was information on exposure status recorded prior to the diet intervention?		Yes	Yes	
	Is it clear exactly when baseline and endpoint		Yes	Yes	

	exposure data was collected relative to start and end of the dietary intervention?				
	Was the timing of baseline and endpoint exposure measures relative to the start and end of the diet consistent between participants?		yes	Yes	
	Could measurement of exposure status have been affected by knowledge of the outcome (mass change)?		Unclear	Unclear	
	What is the predicted direction of bias due to exposure measurement?		Unclear	Unclear	
	reason for your answers			Exposure measured using standard methods. Difficult to know whether a 24 h period gives a good and representative average over a longer time window. Since this is a post hoc study, the researchers would have known weight loss status prior to measuring the chemicals of interest. It is not clear how they ensured that the collection of urine was phthalate or BPA free since this is not the initial intended use of the urine	
	Overall assessment of bias due to exposure measurement		Low	Moderate	Moderate
	How is the exposure measured in the individual?	Grandjean	using a direct method from a sample taken from the person	using a direct method from a sample taken from the person	

	Is the exposure measured in an appropriate and validated matrix?		Yes	Yes	
	Is the exposure measurement from a single spot sample or more than one measurement (eg 24 h urine)?		Single point sample	Single point sample	
	Is it appropriate for exposure to be estimated from the sampling regime use?		Yes	Yes	
	Is the exposure measured in way that is either the gold standard for the chemical or another well accepted way?		Yes	Yes	
	Is a single chemical measured or are multiple congeners/ metabolites within a given group measured?		Several chemicals within a group measured and reported as a sum and individually	Several chemicals within a group measured and reported as a sum and individually	
	Is there a concern that the variation in exposure levels was insufficient to identify associations with mass loss rate or glycaemic control?		Yes	No	
	Was information on exposure status recorded		Yes	Yes	

	prior to the diet intervention?				
	Is it clear exactly when baseline and endpoint exposure data was collected relative to start and end of the dietary intervention?		Party	Yes	
	Was the timing of baseline and endpoint exposure measures relative to the start and end of the diet consistent between participants?		Unsure	Yes	
	Could measurement of exposure status have been affected by knowledge of the outcome (mass change)?		Unsure	No	
	What is the predicted direction of bias due to exposure measurement?		Unsure	No overall effect	
	reason for your answers		For some of the chemicals there is not big enough a range to see a difference e.g. PFDA and depends on how it's been stratified	exposures measured a long time after the original study but uses gold standard methods. There is fourfold difference between people in levels which should be adequate to see a difference if one exists. Uses information from previous studies showing stability of PFAS in blood over extended periods.	
	Overall assessment of bias due to exposure measurement		Moderate	Low	Moderat e

	How is the exposure measured in the individual ?	Kahleova	using a direct method from a sample taken from the person	using a direct method from a sample taken from the person	
	Is the exposure measured in an appropriate and validated matrix?		yes	yes	
	Is the exposure measurement from a single spot sample or more than one measurement (e.g. 24 h urine)?		single point sample	single point sample	
	Is it appropriate for exposure to be estimated from the sampling regime use?		Unsure	Yes	
	Is the exposure measured in way that is either the gold standard for the chemical or another well accepted way?		unsure	unsure	
	Is a single chemical measured or are multiple congeners/ metabolites within a given group measured?		Several chemicals measured and reported individually	Several chemicals measured and reported individually	
	Is there a concern that the variation in exposure levels was insufficient to identify associations with		yes	no	

	mass loss rate or glycaemic control?				
	Was information on exposure status recorded prior to the diet intervention?		Yes	Yes	
	Is it clear exactly when baseline and endpoint exposure data was collected relative to start and end of the dietary intervention?		Yes	Yes	
	Was the timing of baseline and endpoint exposure measures relative to the start and end of the diet consistent between participants?		Yes	Yes	
	Could measurement of exposure status have been affected by knowledge of the outcome (mass change)?		No	No	
	What is the predicted direction of bias due to exposure measurement?		unclear	unclear	
	reason for your answers		very tight variability in POP levels	It seems that standards were not used for all the POP measured and that the levels were not lipid corrected. It also seems that the change in POPs rather than the POP levels at start were used as the explanatory variable in the analysis. POPs are all measured after an overnight fast.	

	Overall assessment of bias due to exposure measurement		Low	Moderate	Moderat e
	How is the exposure measured in the individual ?	Imbeault	using a direct method from a sample taken from the person	using a direct method from a sample taken from the person	
	Is the exposure measured in an appropriate and validated matrix?		yes	yes	
	Is the exposure measurement from a single spot sample or more than one measurement (eg 24 h urine)?		single point sample	single point sample	
	Is it appropriate for exposure to be estimated from the sampling regime use?		unsure	Yes	
	Is the exposure measured in way that is either the gold standard for the chemical or another well accepted way?		unsure	yes	
	Is a single chemical measured or are multiple congeners/ metabolites within a given group measured?		Several chemicals measured and reported individually	Several chemicals measured and reported individually	
	Is there a concern that the variation in exposure		No	No	

	levels was insufficient to identify associations with mass loss rate or glycaemic control?				
	Was information on exposure status recorded prior to the diet intervention?		Yes	Yes	
	Is it clear exactly when baseline and endpoint exposure data was collected relative to start and end of the dietary intervention?		Yes	no	
	Was the timing of baseline and endpoint exposure measures relative to the start and end of the diet consistent between participants?		Yes	unclear	
	Could measurement of exposure status have been affected by knowledge of the outcome (mass change)?		Unclear	no	
	What is the predicted direction of bias due to exposure measurement?		unclear	unclear	
	reason for your answers			It is not clear when the pre weight loss sample was taken relative to start of diet or when after sample was taken following weight loss. It is the change in the POPs rather than absolute values that is	

				reported, and this means the change is happening concurrently with weight loss rather than the reporting of the prior contaminant level.	
	Overall assessment of bias due to exposure measurement		Moderate		Moderate
5. missing data	Was follow up complete (ie was there missing data)?	Liu	no	yes	
	Were the reasons for loss to follow up described and explored?		Unclear	NA	
	Was there adequate completion (ie over 80% of participants followed through to completion) to prevent bias?		Yes	yes	
	Were strategies used to address incomplete follow up, i.e., considering time in the denominator.		NA	NA	
	Are the proportion of participants and reasons for missing data similar across exposures?		Unclear	NA	
	What is the predicted direction of bias due to missing data?		Unclear	No overall effect	
	reason for your answers		Probably minimal because >80% completed which is high.	Note that the original study did have some drop out but overall retention was still above 80% and they report no differences in baseline between people on different diets and between those who were followed up for 2 years and those who were drop outs. This study only reports on those people who were measured throughout	

	Overall assessment of bias due to missing data		Low	Low	Low
	Was follow up complete (i.e. was there missing data)?	Ustulin	No	Unclear	
	Were the reasons for loss to follow up described and explored?		NA	No	
	Was there adequate completion (i.e. over 80% of participants followed through to completion) to prevent bias?		Yes	Unclear	
	Were strategies used to address incomplete follow up, i.e., considering time in the denominator.		Yes	No	
	Are the proportion of participants and reasons for missing data similar across exposures?		NA	Unclear	
	What is the predicted direction of bias due to missing data?		Unsure	Unsure	
	reason for your answers			We have no data on people who dropped out early or didn't complete. We do know that 80% of selected people lost weight. reasons for drop out not mentioned and probably not possible to identify	
	Overall assessment of bias due to missing data		Low		Moderate

	Was follow up complete (ie was there missing data)?	Van der Meer	unclear	yes	
	Were the reasons for loss to follow up described and explored?		Partly	NA	
	Was there adequate completion (ie over 80% of participants followed through to completion) to prevent bias?		Yes	Yes	
	Were strategies used to address incomplete follow up, i.e., considering time in the denominator.		no	NA	
	Are the proportion of participants and reasons for missing data similar across exposures?		Unclear	NA	
	What is the predicted direction of bias due to missing data?		unclear	Unclear	
	reason for your answers		More detail on participants dropping out in Soenen et al (2012) but participant numbers are different.	Note that this is a post hoc study, so they only used participants for whom the 0- and 3-month samples were available, so they have 100% follow up. Sample size is similar between the diets. In Soenen et al (2012) they do report the drop outs from the study BUT the overall sample size is smaller so that means the van der Meer study contains more participants and thus we don't know dropout rates for the total study and thus we don't know if those with high exposures were more likely to drop out or if people who dropped out because they struggled to lose weight were those with higher exposure	

	Overall assessment of bias due to missing data		Low	Moderate	Low
	Was follow up complete (ie was there missing data)?	Grandjean	Unclear	No	
	Were the reasons for loss to follow up described and explored?		No	No	
	Was there adequate completion (i.e. over 80% of participants followed through to completion) to prevent bias?		No	Yes	
	Were strategies used to address incomplete follow up, i.e., considering time in the denominator.		Unclear	NA	
	Are the proportion of participants and reasons for missing data similar across exposures?		Unclear	NA	
	What is the predicted direction of bias due to missing data?		Leads to underestimate of apparent effect	No overall effect	
	reason for your answers		People may drop out if no weight loss (but could this lack of motivation be related to exposure?)	There are no missing data because this is post hoc analysis of only those for which all data exist and for which there were available samples. Issues with reporting dealt with in domain 7	
	Overall assessment of bias due to missing data		Moderate	Low	Low

	Was follow up complete (i.e. was there missing data)?	Kahleova	Yes	No	
	Were the reasons for loss to follow up described and explored?		Yes	Yes	
	Was there adequate completion (i.e. over 80% of participants followed through to completion) to prevent bias?		Yes	Yes	
	Were strategies used to address incomplete follow up, i.e., considering time in the denominator.		Yes	Unclear	
	Are the proportion of participants and reasons for missing data similar across exposures?		Yes	Yes	
	What is the predicted direction of bias due to missing data?		No overall effect	Unclear	
	reason for your answers		ref 15 describes intention to treat protocol so all randomised participants included in final analysis regardless of whether they completed the diet and exercise intervention.	Kahleova et al 2011 reports that only 35 in the vegetarian group and 34 in the normal diet completed to 3 months. In each case lack of motivation was the reason given, plus in control group 2 had other personal reasons. If those who gave up also had high exposures (or low exposures) we might see different results if they had stayed to the end. It is not clear how drop out was dealt with	
	Overall assessment of bias due to missing data		Low	Low	Low

	Was follow up complete (i.e. was there missing data)?	Imbeault	Yes	No	
	Were the reasons for loss to follow up described and explored?		NA	NA	
	Was there adequate completion (i.e. over 80% of participants followed through to completion) to prevent bias?		Yes	Yes	
	Were strategies used to address incomplete follow up, i.e., considering time in the denominator.		NA	NA	
	Are the proportion of participants and reasons for missing data similar across exposures?		NA	NA	
	What is the predicted direction of bias due to missing data?		NA	Unclear	
	reason for your answers		Non-compliance and dropout rate were not mentioned so assuming 100% completion	Other studies by this group use different numbers of participants so it's not clear whether some people were excluded from this specific analysis and if so why, or why this subgroup were chosen.	
	Overall assessment of bias due to missing data		Low	Moderate	Low
6.	How was body mass and/ or the presence of obesity/ overweight assessed?	Liu	in a clinical setting by a trained professional	in a clinical setting by a trained professional	

	If diabetes or IGT or blood glucose was assessed (our secondary outcome), how was this done?		previous diagnosis	previous diagnosis	
	Were other secondary outcomes measured in a standard way?		unclear	Yes	
	Was allocation to diet concealed from the study co-ordinators/ those assigning the patients?		Yes	Yes	
	Were outcomes assessors blind to treatment assignment or exposure status?		Yes	Yes	
	Is participants' body mass and/ or BMI at recruitment reported?		Yes	Yes	
	Is it clear exactly when baseline and endpoint outcome data were collected relative to start and end of the dietary intervention?		Yes	Yes	
	Was the timing of baseline and endpoint outcome measures relative to the start and end of the diet consistent between participants?		Yes	Yes	

	What is the predicted direction of bias due to outcome measurement?		unclear	NA	
	reason for your answers		Could be either over or underestimate	No evidence that outcomes were measured in anything other than highest standards and by clinical professionals who were blinded to diet intervention	
	Overall assessment of bias due to outcome measurement		Low	Low	Low
	How was body mass and/ or the presence of obesity/ overweight assessed?	Ustulin	self-reported mass	self-reported mass	
	If diabetes or IGT or blood glucose was assessed (our secondary outcome), how was this done?		NA: not a reported outcome	NA: not a reported outcome	
	Were other secondary outcomes measured in a standard way?		No	No	
	Was allocation to diet concealed from the study co-ordinators/ those assigning the patients?		Unreported	Unreported	
	Were outcomes assessors blind to treatment assignment or exposure status?		No	Unclear	
	Is participants' body mass and/ or BMI at recruitment reported?		Yes	Yes	

	Is it clear exactly when baseline and endpoint outcome data were collected relative to start and end of the dietary intervention?		Yes	No	
	Was the timing of baseline and endpoint outcome measures relative to the start and end of the diet consistent between participants?		Yes	no	
	What is the predicted direction of bias due to outcome measurement?		Unsure	Unsure	
	reason for your answers		Self-reported height and weight (BMI)	it seems different people have different durations and detail of information throughout, and all information is self-reported	
	Overall assessment of bias due to outcome measurement				
	How was body mass and/ or the presence of obesity/ overweight assessed?	Van der Meer	in a clinical setting by a trained professional using standardised means	in a clinical setting by a trained professional using standardised means	
	If diabetes or IGT or blood glucose was assessed (our secondary outcome), how was this done?		NA: not a reported outcome	NA: not a reported outcome	
	Were other secondary outcomes measured in a standard way?		Unclear	Yes	

	Was allocation to diet concealed from the study co-ordinators/ those assigning the patients?		No	Unreported	
	Were outcomes assessors blind to treatment assignment or exposure status?		No	Unclear	
	Is participants' body mass and/ or BMI at recruitment reported?		yes	yes	
	Is it clear exactly when baseline and endpoint outcome data were collected relative to start and end of the dietary intervention?		yes	yes	
	Was the timing of baseline and endpoint outcome measures relative to the start and end of the diet consistent between participants?		yes	yes	
	What is the predicted direction of bias due to outcome measurement?		unclear	No overall effect	
	reason for your answers		Details of how measurements were obtained have been provided and these are standard and detailed but there is not discussion	The blinding of experimenters is unclear because actually the people were not assigned in a random way but were assigned on basis of sex and BMI and activity so actually the groups should be artificially well balanced. None of the experimenters at the time of measurement could know the patients' exposure measures but they could know the diets so if there as an expectation based on diet this would	

			about the qualification or training of the individual making the measurements	appear as a diet effect in the analysis not a contaminant effects, so I don't think outcome measurement adds much to risk of bias here even with potential for lack of randomisation and blinding	
	Overall assessment of bias due to outcome measurement		Low	Low	Low
	How was body mass and/ or the presence of obesity/ overweight assessed?	Grandjean	in a clinical setting by a trained professional using standardised means to measure body mass; in a clinical setting by a trained professional using standardised measures of body composition;	in a clinical setting by a trained professional using standardised means to measure body mass; in a clinical setting by a trained professional using standardised measures of body composition;	
	If diabetes or IGT or blood glucose was assessed (our secondary outcome), how was this done?		NA	In a laboratory from a blood sample taken at a standard time after eating by a clinically trained person using a gold standard clinical chemistry analyser (see Goyenechea et al (2011)	
	Were other secondary outcomes measured in a standard way?		Unclear	Yes	
	Was allocation to diet concealed from the study co-ordinators/ those assigning the patients?		No	No	
	Were outcomes assessors blind to treatment assignment or exposure status?		No	No	
	Is participants' body mass and/ or BMI at recruitment reported?		Yes	Yes	

	Is it clear exactly when baseline and endpoint outcome data were collected relative to start and end of the dietary intervention?		Yes	Yes	
	Was the timing of baseline and endpoint outcome measures relative to the start and end of the diet consistent between participants?		Yes	Yes	
	What is the predicted direction of bias due to outcome measurement?		Unsure	No overall effect	
	reason for your answers		Composition of weight loss is not captured	Outcomes clearly measured at start and end of the LCD in a clinic, and they would have had a. no knowledge of the exposures and b. all people on same LCD.	
	Overall assessment of bias due to outcome measurement		Low	Low	Low
	How was body mass and/ or the presence of obesity/ overweight assessed?	Kahleova	in a clinical setting by a trained professional using standardised means	in a clinical setting by a trained professional using standardised means	
	If diabetes or IGT or blood glucose was assessed (our secondary outcome), how was this done?		In a laboratory from a blood sample taken at a standard time after eating by a clinically trained person using a gold standard clinical chemistry analyser	In a laboratory from a blood sample taken at a standard time after eating by a clinically trained person using a gold standard clinical chemistry analyser	

	Were other secondary outcomes measured in a standard way?		Unclear	Yes	
	Was allocation to diet concealed from the study co-ordinators/ those assigning the patients?		Unreported	Unreported	
	Were outcomes assessors blind to treatment assignment or exposure status?		Unclear	Unclear	
	Is participants' body mass and/ or BMI at recruitment reported?		Yes	Yes	
	Is it clear exactly when baseline and endpoint outcome data were collected relative to start and end of the dietary intervention?		Yes	Yes	
	Was the timing of baseline and endpoint outcome measures relative to the start and end of the diet consistent between participants?		yes	yes	
	What is the predicted direction of bias due to outcome measurement?		unclear	NA	
	reason for your answers			measurement done on outpatient basis at time of diet onset. Lack of blinding to diet unlikely to influence results here	

	Overall assessment of bias due to outcome measurement		Low	Low	Low
	How was body mass and/ or the presence of obesity/ overweight assessed?	Imbeault	in a clinical setting by a trained professional using standardised means	in a clinical setting by a trained professional using standardised means	
	If diabetes or IGT or blood glucose was assessed (our secondary outcome), how was this done?		In a laboratory from a blood sample taken at a standard time after eating by a clinically trained person using a gold standard clinical chemistry analyser	In a laboratory from a blood sample taken at a standard time after eating by a clinically trained person using a gold standard clinical chemistry analyser	
	Were other secondary outcomes measured in a standard way?		Unclear	yes	
	Was allocation to diet concealed from the study co-ordinators/ those assigning the patients?		Unreported	No	
	Were outcomes assessors blind to treatment assignment or exposure status?		Unclear	No	
	Is participants' body mass and/ or BMI at recruitment reported?		Yes	Yes	
	Is it clear exactly when baseline and endpoint outcome data were collected relative to start and end of the dietary intervention?		Yes	No	

	Was the timing of baseline and endpoint outcome measures relative to the start and end of the diet consistent between participants?		Yes	Unclear	
	What is the predicted direction of bias due to outcome measurement?		Unclear	Unclear	
	reason for your answers		All participants on diet but blinding and randomisation procedures for allocation to fenfluramine/placebo are unclear.	Authors are not specific on timings. The outcomes themselves are measured in a standard way	
	Overall assessment of bias due to outcome measurement		Low	Low	Low
7. reporting and statistical analyses	Was power analysis performed?	Liu	Yes (See Sacks et al 2009)	No	
	Is the reported effect (or lack thereof) likely due to inadequate power/ sample size?		No	No	
	Is the methods section detailed enough to identify which statistical techniques were used?		Yes	Yes	
	Are the statistical techniques used to assess impact of		Yes	Yes	

	chemical on mass loss or glycaemic control appropriate?				
	Was repeated measures analysis used where appropriate?		Unclear	Yes	
	Was the study protocol available and were all of the study's pre-specified primary and secondary outcomes reported in the current manuscript?		Yes	No	
	If the study protocol was not available, was it clear that the published report included all expected outcomes (i.e. comparing methods and results section)?		Unclear	No	
	Is the reported effect estimate likely to be selected from multiple outcome measurements?		No	Yes	
	Is the reported effect estimate likely to be selected from multiple analyses of the exposure-outcome relationship?		No	Unclear	
	Is the reported effect estimate likely to be selected from different subgroups?		No	No	

	What is the predicted direction of bias due to statistics and reporting?		Unclear	Leads to overestimate of effect	
	reason for your answers		Stats not well explained; estimated marginal means calculated but why and according to what group? Risk of overfitting	Glycaemic control indices were measured in the original study (See Sacks et al 2009 supp mat) but not analysed here in same way as other metrics. Multiple analysis done using both LME and stratified by different groups plus quantile analysis. Why was it all needed? Why quartiles?	
	Overall assessment of bias due to reporting of results and statistical analysis		Low	Moderate	Moderat e
	Was power analysis performed?	Ustulin	No	No	
	Is the reported effect (or lack thereof) likely due to inadequate power/ sample size?		No	No	
	Is the methods section detailed enough to identify which statistical techniques were used?		No	No	
	Are the statistical techniques used to assess impact of chemical on mass loss or glycaemic control appropriate?		NA	Unsure	
	Was repeated measures analysis used where appropriate?		Yes	Yes	
	Was the study protocol available and were all of		No	No	

	the study's pre-specified primary and secondary outcomes reported in the current manuscript?				
	If the study protocol was not available, was it clear that the published report included all expected outcomes (i.e., comparing methods and results section)?		Yes	Unclear	
	Is the reported effect estimate likely to be selected from multiple outcome measurements?		Unclear	Unclear	
	Is the reported effect estimate likely to be selected from multiple analyses of the exposure-outcome relationship?		Unclear	No	
	Is the reported effect estimate likely to be selected from different sub groups?		Unclear	No	
	What is the predicted direction of bias due to statistics and reporting?		Leads to overestimate of effect	Unsure	
	reason for your answers		Figure 3 interpretation is questionable. Significant evidence for a very weak/no correlation	Very uncertain how pollution data is derived and matched to the people losing weight.	

	Overall assessment of bias due to reporting of results and statistical analysis			Moderate	
	Was power analysis performed?	Van der Meer	No	No	
	Is the reported effect (or lack thereof) likely due to inadequate power/ sample size?		No	No	
	Is the methods section detailed enough to identify which statistical techniques were used?		Yes	Yes	
	Are the statistical techniques used to assess impact of chemical on mass loss or glycaemic control appropriate?		Yes	Yes	
	Was repeated measures analysis used where appropriate?		Yes	Yes	
	Was the study protocol available and were all of the study's pre-specified primary and secondary outcomes reported in the current manuscript?		Yes	Unclear	
	If the study protocol was not available, was it clear that the published report included all expected		Unclear	No	

	outcomes (i.e. comparing methods and results section)?				
	Is the reported effect estimate likely to be selected from multiple outcome measurements?		No	Yes	
	Is the reported effect estimate likely to be selected from multiple analyses of the exposure-outcome relationship?		Yes	No	
	Is the reported effect estimate likely to be selected from different subgroups?		No	No	
	What is the predicted direction of bias due to statistics and reporting?		No overall effect	Unclear	
	reason for your answers			seems like only the adiposity traits that showed effect are reported. Not clear why longer follow up period was not reported	
	Overall assessment of bias due to reporting of results and statistical analysis		Low	Moderate	Moderat e
	Was power analysis performed?	Grandjea n	No	Yes	
	Is the reported effect (or lack thereof) likely due to inadequate power/ sample size?		No	No	

	Is the methods section detailed enough to identify which statistical techniques were used?		Yes	Yes	
	Are the statistical techniques used to assess impact of chemical on mass loss or glycaemic control appropriate?		Yes	Yes	
	Was repeated measures analysis used where appropriate?		Yes	Yes	
	Was the study protocol available and were all the study's pre-specified primary and secondary outcomes reported in the current manuscript?		No	No	
	If the study protocol was not available, was it clear that the published report included all expected outcomes (i.e., comparing methods and results section)?		Yes	No	
	Is the reported effect estimate likely to be selected from multiple outcome measurements?		No	Yes	
	Is the reported effect estimate likely to be		No	No	

	selected from multiple analyses of the exposure-outcome relationship?				
	Is the reported effect estimate likely to be selected from different subgroups?		No	Yes	
	What is the predicted direction of bias due to statistics and reporting?		Unsure	Unsure	
	reason for your answers		Complete reporting within paper but not in reference to other papers using the same study data. They have reported for everyone in the group by different strata. The data are stratified by weight loss so smaller people will fall into the lower absolute mass loss categories but may have lost a larger % of weight	Only mass is reported on but not BMI or other things that were measured	
	Overall assessment of bias due to reporting of results and statistical analysis		Low	Moderate	Moderat e
	Was power analysis performed?	Kahleova	No	No	
	Is the reported effect (or lack thereof) likely due to inadequate power/ sample size?		No	Unclear	

	Is the methods section detailed enough to identify which statistical techniques were used?		Yes	no	
	Are the statistical techniques used to assess impact of chemical on mass loss or glycaemic control appropriate?		Yes	Yes	
	Was repeated measures analysis used where appropriate?		Yes	Yes	
	Was the study protocol available and were all the study's pre-specified primary and secondary outcomes reported in the current manuscript?		Unclear	no	
	If the study protocol was not available, was it clear that the published report included all expected outcomes (i.e., comparing methods and results section)?		Yes	Unclear	
	Is the reported effect estimate likely to be selected from multiple outcome measurements?		No	Yes	
	Is the reported effect estimate likely to be		No	Unclear	

	selected from multiple analyses of the exposure-outcome relationship?				
	Is the reported effect estimate likely to be selected from different subgroups?		No	No	
	What is the predicted direction of bias due to statistics and reporting?		No overall effect	Leads to overestimate of effect	
	reason for your answers			Correlations are explored for glycaemic control but not weight loss parameters and adiposity, but these were measured. Good to see correlations not regressions but not clear why starting POPs were not used as explanatory variables rather than delta POPs in the investigation of effect of POPs on glycaemic control.	
	Overall assessment of bias due to reporting of results and statistical analysis		Low	Moderate	Moderat e
	Was power analysis performed?	Imbeault	No	No	
	Is the reported effect (or lack thereof) likely due to inadequate power/ sample size?		Unsure	Unsure	
	Is the methods section detailed enough to identify which statistical techniques were used?		Yes	Yes	
	Are the statistical techniques used to assess impact of chemical on mass loss or		Yes	Unsure	

	glycaemic control appropriate?				
	Was repeated measures analysis used where appropriate?		Yes	Yes	
	Was the study protocol available and were all the study's pre-specified primary and secondary outcomes reported in the current manuscript?		Unclear	No	
	If the study protocol was not available, was it clear that the published report included all expected outcomes (i.e. comparing methods and results section)?		Yes	No	
	Is the reported effect estimate likely to be selected from multiple outcome measurements?		No	Yes	
	Is the reported effect estimate likely to be selected from multiple analyses of the exposure-outcome relationship?		No	Unclear	
	Is the reported effect estimate likely to be selected from different sub groups?		No	Unclear	

	What is the predicted direction of bias due to statistics and reporting?		Unclear	Leads to overestimate of effect	
	reason for your answers			This is one of 3 companion papers that all use 37 people on the same diet reporting different outcomes. However, different POPs are reported across these papers so there may be selective reporting of the exposure for most dramatic outcome. In addition, Chevrier et al 2000 use 39 people and Tremblay et al 2004 and Pelletier et al 2002 use 15 and 16. It's not clear if all the studies use a subgroup of the Chevrier study and if so, why only selected participants were included in the analysis. This is hard to tell from the way the information is presented and there may be no overlap Temporality is an issue in terms of the chosen delta POP as the explanatory variable.	
	Overall assessment of bias due to reporting of results and statistical analysis		Low		

Table S14: Characteristics of 17 papers in which body mass or glycaemia control data were collected alongside contaminant data during diet-induced weight loss, but which did not directly explore effects of contaminant on weight loss or glycaemia control directly. Table summarises first author, year of publication, the hypothesis tested, what study the paper was an output from (if from a clinical trial or cluster of publications reporting on the same participants); sample size (n); age of participants; sex ratio (% female); location; diet intervention; diet duration; and outcomes that were both measured and reported (BMI – body mass index; WC= waist circumference; FG = fasting glucose; FI = fasting insulin; GTT = glucose tolerance test; HOMI-IR = homeostatic model assessment of insulin resistance; HOMA-β = homeostatic model assessment of beta cell function). Table indicates only those parameters that were reported in the paper, not those reported in previous publications. Additional measures for the same participants may be reported in other papers from the same study. Shading indicates where that outcome was reported.

author	year	Hypothesis	Study	n	Age	Sex (% F)	Location	Matrix	Diet intervention	Diet duration	Outcomes									
											mass	BMI	WC	% fat	FG	FI	GTT	HOMI-IR	HOMA-β	Hb1Ac
Imbeault [1]	2001	Increase in plasma organochlorine during weight loss will reduce adipocyte basal lipolysis	Laval University	37	36-50	54	Canada	Plasma	Unspecified non macronutrient specific diet of 700kcal/ day	15 weeks										
Imbeault [2]	2002	Increase in plasma organochlorine during weight loss will alter capacity for skeletal muscle oxidation		37	-	54	Canada	Plasma	Unspecified non macronutrient specific diet of 700kcal/ day	15 weeks										

Pelletier [3]	2002	Increase in plasma organochlorine during weight loss will reduce TH and RMR		16	-	0	Canada	Plasma	unspecified non macronutrient specific diet	15 weeks										
Tremblay [4]	2004	Increase in plasma organochlorine during weight loss will reduce thermogenesis/ SMR		15	43.9 ± 4.6	47	Canada	Plasma	Unspecified non macronutrient specific diet of -700kcal/ day	15 weeks										
He [5]	2017	A protein pacing / intermittent fasting calorie restricted diet will reduce oxidative stress and maintain or enhance PCB elimination compared to other typical weight maintenance diets	NCT02525419	40	47.6 ± 9.5	48	USA	Serum	6 days of protein paced calorie restriction and one day per week of intermittent fasting (330-430 kcal per day) during weight loss. High protein powder or bar replacement for 2 of 3 meals per day versus standard 'heart healthy' diet recommendations during weight maintenance	12 week weight loss followed by 52 weeks weight maintenance										

Müllerová [6]	2008	PCBs are associated with plasma adiponectin before or after a low calorie diet	Plzen study	27	21-74	100	Czech Republic	Plasma	1200kcal per day	3 months										
Geens [7]	2015	Chemicals are related to TH levels and anthropometric parameters; exposure sources to chemicals depends on weight loss method	ENDORUP (NCT01778868) KCT0003002	151 at start	18-84	70 at start	Belgium	Urine	Unclear from the paper what the diet was	Unclear. Weight loss occurred over 6 months and then stabilised for last 6 months of 12 month study										
Dirtu [8]	2013	Chemicals are related to anthropometric parameters; exposure sources to chemicals depends on weight loss method		152 at start	18-84	69 at start	Belgium	Urine	Unclear from the paper what the diet was	Unclear Weight loss occurred over 6 months and then stabilised for last 6 months of 12 month study (Geens et al 2015)										

Dirinck [9]	2015	Release of PCBs during weight loss differs between visceral and subcutaneous fat		20	26-60	100	Belgium	Serum	700 kcal protein-enriched diet (n= 18) or 1500 kcal diet (n = 2) increased after 6 weeks by 200k cal/ month	6 weeks initial diet with follow up at 6 months										
Dirtu [10]	2013	To describe the dynamics of PCBs during weight loss in obese patients; compare levels with those from previous work; compare obese and lean subjects		85	As in Geens et al, but not split by which subjects had the diet and which had surgery	?	Belgium	Serum	Conservative weight loss programme with dietary and lifestyle counselling. Details not provided	Unclear but patients followed up for 12 months										
Jung [11]	2020	‘detox’ diet will improve heavy metal elimination and promote weight loss more than a typical calorie restricted diet	Wellness Up	45	26 ± 4.6	100	South Korea	Hair	1215-1225 kcal either as a ‘detox’ diet or as ‘normal’ calorie restricted diet	4 weeks										

Müllerová [12]	2015	Plasma POPs will depend on success of weight loss 5 years after a dietary intervention	Plzen study	20	25-73	100	Czech Republic	Plasma	1200kcal per day consisting of 20% protein and 25-30% fat	3 months with 5 year follow up										
El Soud [13]	2011	Calorie restriction on high protein diet rich in fresh fruit and vegetables will reduce heavy metals	No linked papers	65 (at start) 30 by end	11-14	100	Egypt	Urine	1500k cal per day rich in protein and high in fresh fruit and vegetable (as means to increase antioxidants)	2 months										
Chevrier phase 1 [14]	2000	Body weight loss increases plasma and adipose levels of OCs	Laval University	39	43.4 ± 4.6	54	Canada	Plasma	Non macro-nutrient specific diet of 700kcal/day	15 weeks										
Chevrier phase 2 [14]	2000	Body weight loss increases plasma and adipose levels of OCs		17	43.3 ± 1.6 (M); 40.5 ± 1.6 (F)	42	Canada	Plasma	Low fat diet following phase 1 (30% fat; 17% protein; 53% carbohydrate) combined with physical activity regime	18 weeks after phase 1										
Arguin [15]	2010	Olestra can prevent the increase in plasma OC levels during weight loss	Ole study	37	21-60	0	USA	Plasma	33% fat; 25% fat or 25% fat with remaining fat made up by olestra	3 months (main study lasted 9 months (Bray et al 2002)										

Grundler [16]	2021	Exploration of effect of fasting on heavy metal and glyphosate excretion	DRKS000 16657	109	18-70	62	Germany	Urine Hair	Laxative followed by 250 kcal diet (organic juice, honey and vegetable soup) with fluids. Food reintroduced over 4 days	10 days and four week follow up										
Malarvannan [17]	2018	To describe the dynamics of PCBs during weight loss in obese adolescents		94	11-18	64	Belgium	Serum	Dietary and lifestyle counselling with physical activity and psychological support	5 months										

References:

1. Imbeault P, Chevrier J, Dewailly E et al. Increase in plasma pollutant levels in response to weight loss in humans is related to *in vitro* subcutaneous adipocyte basal lipolysis. *Int J Obesity Related Metabolic Dis* 2001. 25: 1585–1591. https://doi.org/10.1038/sj.ijo.0801817.

2. Imbeault P, Tremblay A, Simoneau JA, Joanisse DR, Weight loss-induced rise in plasma pollutant is associated with reduced skeletal muscle oxidative capacity. *Am J Physiol. Endocrinol Metab.* 2002. 282: E574–E579. https://doi.org/10.1152/ajpendo.00394.2001

3. Pelletier C, Doucet E, Imbeault, P, Tremblay A. Associations between weight loss-induced changes in plasma organochlorine concentrations, serum T(3) concentration, and resting metabolic rate. *Toxicol Sci.* 2002. 67: 46–51. https://doi.org/10.1093/toxsci/67.1.46.

4. Tremblay A, Pelletier C, Doucet E, Imbeault P. Thermogenesis and weight loss in obese individuals: a primary association with organochlorine pollution. *Int J Obesity Related Metab Dis* 2004. 28: 936–939. https://doi.org/10.1038/sj.ijo.0802527

5. He F, Zuo L, Ward E, Arciero PJ. Serum polychlorinated biphenyls increase and oxidative stress decreases with a protein-pacing caloric restriction diet in obese men and women. *Int J Environ Res Public Health.* 2017. 14: 59. https://doi.org/10.3390/ijerph1401005.

6. Müllerová D, Kopecky J, Matějková D et al. Negative association between plasma levels of adiponectin and polychlorinated biphenyl 153 in obese women under non-energy-restrictive regime. *Int J Obesity* 2008. 32: 1875–1878. https://doi.org/10.1038/ijo.2008.169

7. Geens T, Dirtu AC, Dirinck E. et al. Daily intake of bisphenol A and triclosan and their association with anthropometric data, thyroid hormones and weight loss in overweight and obese individuals. *Environ Int.* 2015. 76: 98–105. https://doi.org/10.1016/j.envint.2014.12.003

8. Dirtu AC, Geens T, Dirinck E et al. Phthalate metabolites in obese individuals undergoing weight loss: Urinary levels and estimation of the phthalates daily intake. *Environ Int* 2013.59: 344–353. https://doi.org/10.1016/j.envint.2013.06.023

9. Dirinck E, Dirtu AC, Geens T, Covaci A, Van Gaal L, Jorens PG. Urinary phthalate metabolites are associated with insulin resistance in obese subjects. *Environ. Res.* 2015. 137, 419–423. doi: 10.1016/j.envres.2015.01.010.

10. Dirtu AC, Dirinck E, Malarvannan G et al. Dynamics of organohalogenated contaminants in human serum from obese individuals during one year of weight loss treatment. *Environ Sci Technol.* 2013. 47: 12441–12449. https://doi.org/10.1021/es400657t

11. Jung SJ, Kim WL, Park BH, Lee SO, Chae SW. Effect of toxic trace element detoxification, body fat reduction following four-week intake of the Wellnessup diet: a three-arm, randomized clinical trial. *Nutr Metab* 2020. 17: 47. https://doi.org/10.1186/s12986-020-00465-99.

12. Müllerová D, Matějková D, Dvořáková J, Müller L, Rosmus J, Kovářová K. Persistent organichlorine pollutants in obese women after diet induced weight loss: five years follow up study. *Central European J Pub Health.* 2015. 23: 214–217. https://doi.org/10.21101/cejph.a4100

13. El Soud NHA, Mohsen MA, Joussef M, Kazem Y. Effect of a 2-Month program of antioxidants-micronutrient rich diet on concentrations of lead, cadmium and aluminum in obese Egyptian children. *Macedonian J Med Sci* 2011. 4:290-295. http://dx.doi.org/10.3889/MJMS.1857-5773.2011.0184.

14. Chevrier J, Dewailly E, Ayotte P, Mauriège P, Després, JP, Tremblay A. Body weight loss increases plasma and adipose tissue concentrations of potentially toxic pollutants in obese individuals. *Int J Obesity Related Metab Dis.* 2000. 24: 1272–1278. https://doi.org/10.1038/sj.ijo.0801380

15. Arguin H, Sánchez M, Bray GA et al. Impact of adopting a vegan diet or an olestra supplementation on plasma organochlorine concentrations: results from two pilot studies. *Brit J Nutr.* 2010. 103: 1433–1441. https://doi.org/10.1017/S000711450999331X

16. Grundler F, Séralini GE, Mesnage R, Peynet V, Wilhelmi de Toledo F. Excretion of heavy metals and glyphosate in urine and hair before and after long-term fasting in humans. *Front Nutr.* 2021. 8: 708069. https://doi.org/10.3389/fnut.2021.708069.

17. Malarvannan G, Van Hoorenbeeck K, Deguchtenaere A. et al. Dynamics of persistent organic pollutants in obese adolescents during weight loss. *Environ Int.* 2018.110: 80–87. https://doi.org/10.1016/j.envint.2017.10.009.
